# Living on the edge: Predicting songbird response to management and environmental changes across an ecotone

**DOI:** 10.1002/ece3.10648

**Published:** 2023-11-14

**Authors:** Nicholas J. Van Lanen, Adrian P. Monroe, Cameron L. Aldridge

**Affiliations:** ^1^ U.S. Geological Survey, Fort Collins Science Center Fort Collins Colorado USA; ^2^ Graduate Degree Program in Ecology, Colorado State University Fort Collins Colorado USA; ^3^ Bird Conservancy of the Rockies Brighton Colorado USA

**Keywords:** conifer removal, hierarchical abundance model, non‐target species, pinyon‐juniper, sagebrush, songbirds

## Abstract

Effective wildlife management requires robust information regarding population status, habitat requirements, and likely responses to changing resource conditions. Single‐species management may inadequately conserve communities and result in undesired effects to non‐target species. Thus, management can benefit from understanding habitat relationships for multiple species. Pinyon pine and juniper (*Pinus* spp. and *Juniperus* spp.) are expanding into sagebrush‐dominated (*Artemisia* spp.) ecosystems within North America and mechanical removal of these trees is frequently conducted to restore sagebrush ecosystems and recover Greater Sage‐grouse (*Centrocercus urophasianus*). However, pinyon‐juniper removal effects on non‐target species are poorly understood, and changing pinyon‐juniper woodland dynamics, climate, and anthropogenic development may obscure conservation priorities. To better predict responses to changing resource conditions, evaluate non‐target effects of pinyon‐juniper removal, prioritize species for conservation, and inform species recovery within pinyon‐juniper and sagebrush ecosystems, we modeled population trends and density‐habitat relationships for four sagebrush‐associated, four pinyon‐juniper‐associated, and three generalist songbird species with respect to these ecosystems. We fit hierarchical population models to point count data collected throughout the western United States from 2008 to 2020. We found regional population changes for 10 of 11 species investigated; 6 of which increased in the highest elevation region of our study. Our models indicate pinyon‐juniper removal will benefit Brewer's Sparrow (*Spizella breweri*), Green‐tailed Towhee (*Pipilo chlorurus*), and Sage Thrasher (*Oreoscoptes montanus*) densities. Conversely, we predict largest negative effects of pinyon‐juniper removal for species occupying early successional pinyon‐juniper woodlands: Bewick's Wren (*Thryomanes bewickii*), Black‐throated Gray Warblers (*Setophaga nigrescens*), Gray Flycatcher (*Empidonax wrightii*), and Juniper Titmouse (*Baeolophus ridgwayi*). Our results highlight the importance of considering effects to non‐target species before implementing large‐scale habitat manipulations. Our modeling framework can help prioritize species and regions for conservation action, infer effects of management interventions and a changing environment on wildlife, and help land managers balance habitat requirements across ecosystems.

## Graphical abstract

We used avian point count data (2008–2020) to model regional population trends and density as a function of environmental, climatic, and anthropogenic variables for 11 songbird species to inform future wildlife management within sagebrush and pinyon‐juniper woodland ecosystems. We found regional population changes for 10 of 11 species investigated; 6 of which increased in the highest elevation region of our study. Our results provide inference regarding how environmental change and resource conditions are likely to influence bird density, where species likely occur at high densities across the western United States, and highlight that consideration of non‐target species may enhance multi‐species management for species in this study.

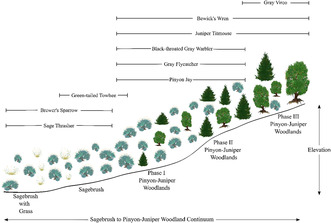

## 1 INTRODUCTION

Acquiring information on the distribution and abundance of animals across landscapes is a focal topic in ecology, biogeography, and conservation biology. Such information can provide insight into species niches, inter‐ and intra‐specific competition, population status, the location of biodiversity hotspots, and conservation planning (Guisan & Thuiller, 2005). Unfortunately, rapid alterations to resource conditions across the globe are occurring due to a changing climate, modified nutrient cycles (Kardol et al., 2010), altered fire regimes (Liu et al., 2010), land‐use change (Winkler et al., 2021), shifting vegetation communities (Lenoir et al., 2008), and broad‐scale habitat management (Kennedy et al., 2012; Monroe et al., 2017). These changing resource conditions limit our ability to predict current and future habitat for wildlife across landscapes, may alter the likelihood of species persistence, and obscure our ability to prioritize species for conservation action.

A changing climate and alterations to landcover represent two of the largest contributors to changing resource conditions and were implicated as major causes of population decline and threats to global biodiversity (Tilman et al., 2017). Efforts to understand resources required to support multiple species led to the use of umbrella, flagship, and keystone species as indicators of ecological integrity (Caro & O'Doherty, 1999; Wilcox, 1984). However, researchers and managers have long been concerned that managing for the needs of a single species, even if carefully chosen, may not address the needs of multiple co‐occurring species (see Roberge & Angelstam, 2004; Simberloff, 1998). Successful multi‐species management becomes even more complex when management actions prioritize one ecosystem over another. For instance, managing for closed canopy forests, rather than open canopy forests, in the Sierra Nevada mountain range to enhance spotted owl (*Strix occidentalis*) habitat is believed to result in decreased occupancy for 90% of the native species occurring in the region, some of which are uncommon. In these instances, management may benefit from considering effects on non‐target species to ensure viable populations of the full suite of species occurring within the region. Specifically, habitat requirements of multiple wildlife species can be evaluated to assess the effects of changing resource conditions upon the community. This evaluation is particularly critical when multiple species across ecosystems are of conservation concern and may be declining within all or portions of their range.

The resources found at the intersection of pinyon‐juniper (*Pinus* spp.; *Juniperus* spp.) woodland and sagebrush‐steppe (*Artemisia* spp.) ecosystems in the western United States support multiple declining wildlife species (Sauer et al., 2020). These ecosystems are experiencing rapid changes to environmental conditions due to anthropogenic development, spread of invasive annual grasses, and a changing climate (Schroeder et al., 2004). In addition, pinyon‐juniper woodlands have been undergoing regional expansion, contraction, and infilling due to changes in land‐use and climate (Amme et al., 2020; Falkowski et al., 2017), representing an additional threat to the sagebrush system. As a result, mechanical removal of pinyon pine (*Pinus edulis* and *Pinus monophylla*) and juniper (*Juniperus monosperma*, *J. osteosperma*, and *J. scopulorum*; hereafter, “pinyon‐juniper removal”) is currently being conducted to reduce the risk and severity of wildfire (Infrastructure Investment and Jobs Act, 2021; Vaillant & Reinhardt, 2017), enhance grazing productivity (Naugle et al., 2019), and restore sagebrush ecosystems (Miller et al., 2017). Numerous studies have identified population‐level benefits to Greater Sage‐Grouse (*Centrocercus urophasianus*; hereafter “sage‐grouse”), an at‐risk species which is closely associated with sagebrush ecosystems, following pinyon‐juniper removal (Baruch‐Mordo et al., 2013; Severson et al., 2017). However, effects of pinyon‐juniper removal on non‐target species occurring within the sagebrush and pinyon‐juniper ecotone are less understood. Recently, experimental studies evaluated wildlife responses to pinyon‐juniper removal, finding mixed effects for species associated with sagebrush and pinyon‐juniper ecosystems (Holmes et al., 2017; Magee et al., 2019), but transferability of these local‐scale findings to disparate regions supporting pinyon‐juniper woodlands is uncertain (Johnson & Sadoti, 2019). As a result, the expected overall response of bird populations within these communities to changing resource conditions associated with climate change, land‐use alterations, expanding pinyon‐juniper woodlands, and pinyon‐juniper removal remains unclear.

To address this need, we developed large‐scale density‐habitat models for 11 songbird species of conservation concern (Partners in Flight, 2021) which are associated with sagebrush (*n* = 4 species), pinyon‐juniper woodlands (*n* = 4), or are considered generalists (*n* = 3) within these ecosystems. We estimated regional population trends to identify which species are declining and in which regions. We assessed spatial scales at which avian density responded to resource conditions to generate robust estimates of density‐habitat relationships and population trends. We then mapped predicted density for each species across the landscape to guide conservation planning. Finally, we used the density‐habitat models to evaluate how a changing climate, pinyon‐juniper expansion and infilling, anthropogenic development, and on‐going management within the sagebrush and pinyon‐juniper ecotone may affect each species.

Our study provides conservation planners with spatially explicit predictions identifying regions capable of supporting high densities of songbirds, robust estimates of regional population trends which account for incomplete detection, and inferences regarding the magnitude and extent to which habitat for these species may change in the future. Our framework can be used to guide the protection of important regions for wildlife, prioritize conservation of species which could be at‐risk from current and future perturbations to the system, and provide land managers with information on expected species‐specific responses to large‐scale vegetation treatments.

## 2 METHODS

### 2.1 Study species

We investigated avian population trends and density‐habitat relationships for 11 songbird species occurring within pinyon‐juniper and sagebrush ecotones and which are of high to moderate conservation concern (Partners in Flight, 2021) at the continental scale: Bewick's Wren (*Thryomanes bewickii*), Brewer's Sparrow (*Spizella breweri*), Black‐throated Gray Warbler (*Setophaga nigrescens*), Gray Flycatcher (*Empidonax wrightii*), Gray Vireo (*Vireo vicinior*), Green‐tailed Towhee (*Pipilo chlorurus*), Juniper Titmouse (*Baeolophus ridgwayi*), Loggerhead Shrike (*Lanius ludovicianus*), Sagebrush Sparrow (*Artemisiospiza nevadensis*), Sage Thrasher (*Oreoscoptes montanus*), and Townsend's Solitaire (*Myadestes townsendi*). Of these species, Black‐throated Gray Warblers, Gray Flycatcher, Gray Vireo, and Juniper Titmouse breed in western forests in North America and are often associated with pinyon‐juniper ecosystems. Similarly, Brewer's Sparrow, Green‐tailed Towhee, Sagebrush Sparrow, and Sage Thrasher frequently breed in sagebrush ecosystems. Finally, Bewick's Wren, Loggerhead Shrike, and Townsend's Solitaire primarily use a mixture of vegetation communities and are best characterized as generalist species with regards to sagebrush and/or pinyon‐juniper ecosystems (Partners in Flight, 2021). Of these species, Breeding Bird Survey (BBS) trend estimates indicate Brewer's Sparrow, Loggerhead Shrike, and Sagebrush Sparrow have declined from 1966 to 2019 (Sauer et al., 2020). Additionally, trend estimates from a large‐scale avian monitoring program indicate Bewick's Wren, Black‐throated Gray Warbler, Gray Flycatcher, Juniper Titmouse, and Sage Thrasher (in addition to Brewer's Sparrow) are declining within portions of their range (Bird Conservancy of the Rockies, 2022).

### 2.2 Study area

**FIGURE 1:**
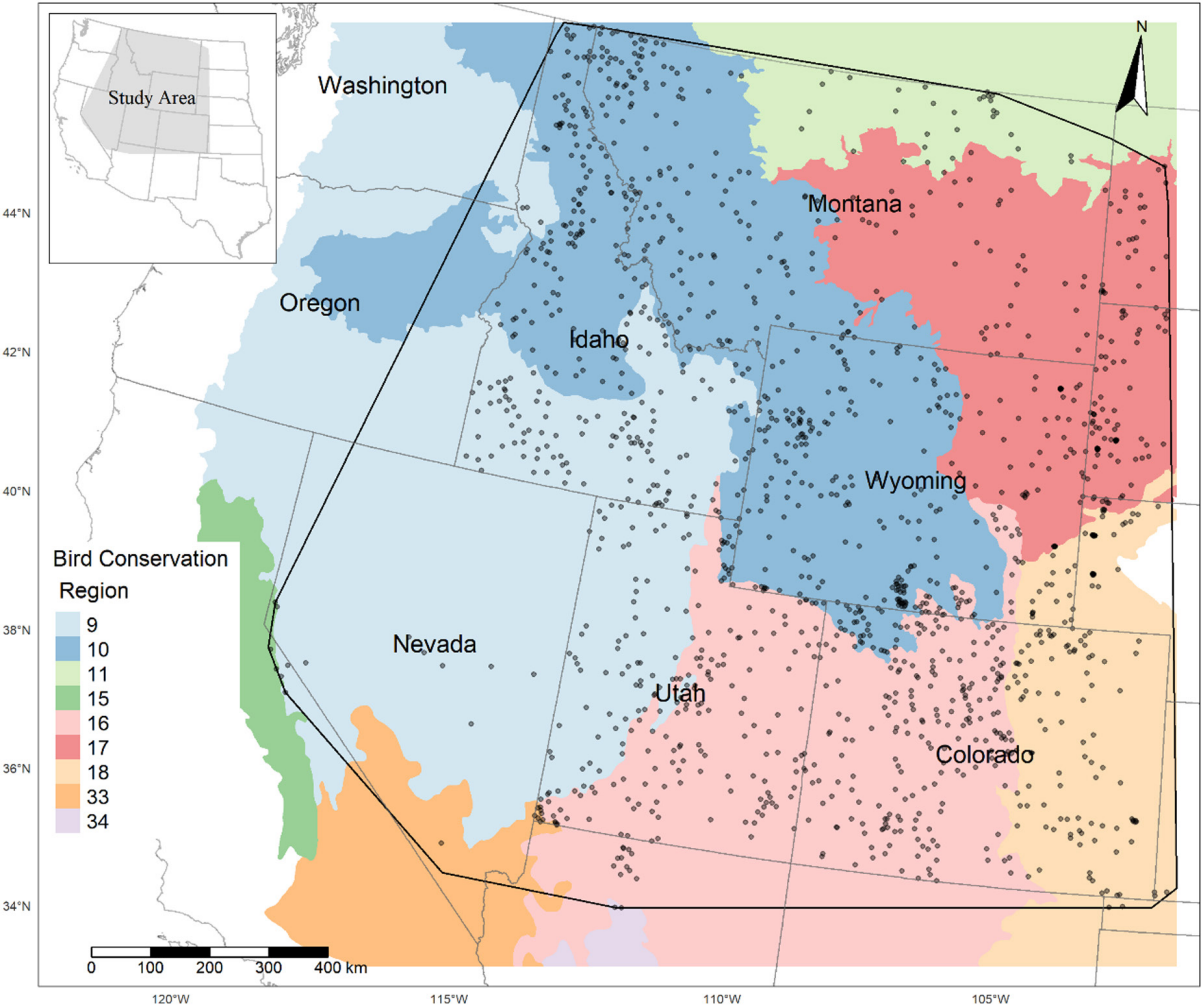
Study area and locations of Integrated Monitoring in Bird Conservation Region surveys included in the modeling of 11 songbird species, 2008–2020. Bird Conservation Regions 9 (Great Basin), 10 (Northern Rockies), 11 (Prairie Potholes), 15 (Sierra Nevada), 16 (Southern Rockies Colorado Plateau), 17 (Badlands and Prairies), 18 (Shortgrass Prairie), 33 (Sonoran and Mojave Deserts), and 34 (Chihuahuan Desert) are shown in color and clipped to the United States of America and figure extents. Bases modified from National Weather Service, 1:2,000,000, 1980 and from Bird Studies Canada and NABCI, 2014 digital data.

Our modeling efforts spanned portions of 13 states in the western United States which support sagebrush and pinyon‐juniper ecotones, where sagebrush and/or pinyon‐juniper management is ongoing, and where robust point count data had been collected (Figure 1): Arizona, California, Colorado, Idaho, Kansas, Montana, North Dakota, Nebraska, Nevada, Oklahoma, South Dakota, Utah, and Wyoming. Our region of interest included portions of nine Bird Conservation Regions (BCRs) representing ecologically distinct regions with similar bird and vegetation communities (United States North American Bird Conservation Initiative Committee, 2000). Bird Conservation Regions represented within our study area included: Great Basin (BCR9), Northern Rockies (BCR10), Prairie Potholes (BCR11), Sierra Nevada (BCR15), Southern Rockies/Colorado Plateau (BCR16), Badlands and Prairies (BCR17), Shortgrass Prairie (BCR18), Sonoran and Mohave Deserts (BCR33), and the Sierra Madre Occidental (BCR34; Figure 1).

### 2.3 Data inputs

#### 2.3.1 Bird data

We used breeding season point count data from the Integrated Monitoring in Bird Conservation Regions (IMBCR) program (Pavlacky Jr. et al., 2017), between 2008 and 2020, to model avian density. The IMBCR program employs a stratified design for sample selection in which IMBCR partners primarily defined strata using land ownership and/or management boundaries. Less frequently, IMBCR partners defined strata using fixed geographic features such as elevation, watersheds, or riverways. Partners with the IMBCR program selected sample units (hereafter, “grids” or “grid cells”), represented by 1‐km^2^ grid cells, using a spatially balanced random algorithm (GRTS: Stevens & Olsen, 2004) and populated each grid cell with 16 point count stations, uniformly arranged and spaced 250 m apart.

Bird surveyors attempted to conduct variable radius point counts for five (2008 and 2009) or six (all subsequent years) minutes at all 16‐point count stations within an IMBCR grid in a single morning (collectively, a “survey”). Surveyors conducted surveys when visibility was good, there was no precipitation, and under low wind conditions (<18 km per hour). Surveyors were frequently unable to complete all avian point counts within the grid due to steep terrain, reduced bird activity, inclement weather, and/or a lack of private landowner permission to survey the property. Surveys began at first light, when birds could be visibly identified, and generally concluded by approximately 5 h after local sunrise. Surveyors recorded the start time of each point count following the 2011 breeding season.

Surveyors recorded the species, horizontal distance (estimated with a laser rangefinder), minute of the count, sex, whether the individual was flying over and not using the habitat, and if the individual might be migrating for each independent detection. When surveyors could not identify an individual, they recorded the species as “unknown.” We removed detections of “unknown” species, potentially migrating individuals, fly‐overs, and observations with missing data from the data set.

We included point count data from all IMBCR grids which were surveyed at least 3 years, were surveyed at least 67% of the years between the first survey year and the last survey year, and which fell within the geographic extent of remotely sensed raster layers used as predictor variables in our analyses (see below, *n* = 1782 grids, Figure 1). For each species, we removed all survey data collected within each BCR in which the species was not detected, resulting in a variable number of grids within the data set for each species. We summarized the number of grids surveyed, number of surveys (grid and year combination), and number of detections within each BCR.

#### 2.3.2 Site visit data

We included two variables in our detection model to account for heterogeneity in daily and seasonal singing rates for each species of interest: “minutes since sunrise” and “ordinal date.” We derived “minutes since sunrise” based on local sunrise time with the “maptools” package in R (Bivand & Lewin‐Koh, 2020) and the average start time of point counts during each survey. Additionally, we developed a binary variable, “observer experience,” to account for any differences in observer skill level. We considered surveyors “experienced” if they conducted IMBCR surveys in a prior breeding season and “inexperienced” if they had not.

#### 2.3.3 Predictor variables

We characterized a variety of landcover, topographic, anthropogenic, and climatic variables in our modeling efforts (Table 1) to improve inferences regarding trends in avian density and the density‐habitat relationships. We used 30‐m resolution Rangeland Condition, Monitoring, Assessment, and Projection (RCMAP) Time Series raster products (Rigge et al., 2021) representing percent cover of annual herbaceous (annual grass and forbs; primarily invasive), herbaceous (primarily perennial grasses, forbs, and cacti), litter (dead plant material), and sagebrush (Table 1). These layers provided annual estimates of each variable and were available for 2008–2011 and 2013–2020. We derived 2012 values by averaging pixel values from 2011 to 2013. Following Van Lanen et al. (2023a), we reclassified 30‐m resolution LANDFIRE existing vegetation type (EVT) raster layers to develop a binary pinyon‐juniper landcover raster layer (Table 1). We associated the binary pinyon‐juniper cover layer derived from LANDFIRE (2008) with 2008–2009, LANDFIRE (2010) with 2010–2011, LANDFIRE (2012) with 2012–2013, LANDFIRE (2014) with 2014–2015, and LANDFIRE (2016) with 2016–2020. We developed a raster layer representing agricultural lands by reclassifying CropScape Cropland Data Layers (United States Department of Agriculture 2008–2020) as a binary variable for each year of our study following procedures detailed by Van Lanen et al. (2023a) (Table 1).

**TABLE 1:** Spatial data used as predictive covariates in density‐habitat relationship modeling of 11 songbird species across much of the western United States; 2008–2020.

Covariate	Source data	Source resolution	Covariate description	Scale of covariate summarization
Annual Herbaceous Cover	RCMAP^a^	30‐m	% cover of annual grasses and forbs (primarily invasive)	Mean values at 101 buffer extents (0 m–10,000 m) from IMBCR grid centroid
Herbaceous Cover	RCMAP^a^	30‐m	% cover of perennial forbs	Mean values at 101 buffer extents (0 m–10,000 m) from IMBCR grid centroid
Litter Cover	RCMAP^a^	30‐m	% cover of dead/residual plant material	Mean values at 101 buffer extents (0 m–10,000 m) from IMBCR grid centroid
Sagebrush Cover	RCMAP^a^	30‐m	% cover of sagebrush canopy	Mean values at 101 buffer extents (0 m–10,000 m) from IMBCR grid centroid
Pinyon‐juniper Cover	Reclassified from LANDFIRE^b^	30‐m	Proportion of raster cells within buffer extents representing pinyon‐juniper woodland	Proportion of pinyon‐juniper cover at 101 buffer extents (0 m–10,000 m) from IMBCR grid centroid
Cropland Cover	Reclassified from CropScape^c^	30‐m	Proportion of raster cells within buffer extents representing planted row crops and orchards	Proportion of cropland cover at 101 buffer extents (0 m–10,000 m) from IMBCR grid centroid
Elevation	USDA 2007^d^	30‐m	Mean elevation in m	564‐m radii from grid cell centroid
Vector Ruggedness Measure	O'Donnell et al. (2019)^e^	30‐m	Mean terrain ruggedness	564‐m radii from grid cell centroid
Linear Disturbance	BLM (unpublished)	30‐m	Proportion of raster cells within 564 m radii which contain at least a single linear disturbance feature	Mean values at 101 buffer extents (0 m–10,000 m) from IMBCR grid centroid
Point Disturbance	BLM (unpublished)	30‐m	Proportion of raster cells within 564 m radii which contain at least a single point disturbance feature	Mean values at 101 buffer extents (0 m–10,000 m) from IMBCR grid centroid
Normalized Difference Vegetation Index	MOD13Q1 MODIS^f^	30‐m	Standardized measure of vegetation	Mean of each pixel's maximum NDVI value from May to July at 101 buffer extents (0 m–10,000 m) from IMBCR grid centroid
Palmer Drought Severity Index	Historical Drought Severity Index^g^	Climate Division	Measure of drought severity calculated using temperature and precipitation	Values associated with climate division extracted at IMBCR grid centroid

*Note*: The covariate name (Covariate) used in the manuscript, data source (Source data), spatial resolution of source data layers (Source resolution), description of covariate after processing (Covariate description), and spatial scales at which covariates were summarized (Scale of covariate summarization) are shown.

^a^ Rigge et al. (2021).

^b^ LANDFIRE (2008, 2010, 2012, 2014, 2016).

^c^ United States Department of Agriculture (2008–2020).

^d^ United States Department of Agriculture (USDA) Natural Resources Conservation Service (2007).

^e^ O'Donnell et al. (2019).

^f^ Didan (2015).

^g^ National Centers for Environmental Information (2020).

We included two topographic predictor variables we suspected could influence bird abundance directly (e.g., preference for flat, open regions) or indirectly (e.g., by influencing temperature): elevation and vector ruggedness measure (vrm) (Table 1). To characterize elevation within IMBCR grids, we obtained a 30‐m digital elevation model (DEM) from National Elevation Dataset (United States Department of Agriculture (USDA) Natural Resources Conservation Service, 2007). We obtained a vector ruggedness measure (vrm) layer representing a measure of elevation difference, aspect, and slope (Sappington et al., 2007) developed by O'Donnell et al. (2019) (Table 1).

We acquired two 30‐m resolution raster layers representing point and linear features relating to anthropogenic disturbance on the landscape (Table 1), as road and energy development may influence occupancy and abundance of some sagebrush‐associated songbirds (Gilbert & Chalfoun, 2011; Latif et al., 2023; Mutter et al., 2015). These anthropogenic disturbance layers were created by the Wildlife Habitat Spatial Analysis Lab of the Bureau of Land Management's (BLM) National Operations Center (Table 1; Data Contact: AFMSS Program Manager Michael Mulder). The pixel values for these disturbance layers represent the proportion of pixels within a 564‐m radius of each raster pixel centroid which includes at least one anthropogenic feature. The point disturbance raster was developed by the BLM via a point density analysis on 12 disturbance point feature classes. These feature classes included power plant locations identified via S&G Platt, digital obstacle data within Federal Aviation Administration Regions, resource extraction well locations (both active and inactive) reported by the Automated Fluid Minerals Support System, and communication tower locations provided by the Federal Communications Commission prior to October of 2016. The BLM created the linear disturbance raster layer using a line density analysis derived from a layer which combined eight feature classes: highways, major roads, and city streets from the ESRI Street Maps Premium ArcGIS dataset; railroads from the Federal Railroad Administration Rail Network database; and four layers representing above‐ground transmission lines (of varying voltages) from the S&P Platt Transmission Line geospatial layer (unpublished).

We included two predictive layers to describe climatic conditions and account for indirect effects (e.g., food availability) of climate on avian abundance (Table 1). We developed a raster layer for each year representing potential vegetation productivity (Monroe et al., 2017) by calculating the maximum Normalized Difference Vegetation Index (NDVI) observed between 1 May and 31 July from MOD13Q1 MODIS data (Didan, 2015). We also calculated annual mean summertime (May, June, and July) Palmer Drought Severity Index (PDSI) values (with higher values indicating cooler and wetter weather) for each climate division polygon within our study area (Table 1; National Centers for Environmental Information, 2020).

We extracted PDSI values from the climate division encompassing the grid centroid for each grid cell. We extracted mean elevation and vrm values using a 564‐m radius surrounding the grid centroid, which was sufficient to include all 16 point count stations in the grid (Table 1). The scale at which resource conditions influence species‐environment relationships can vary by both resource type and species (Frishkoff et al., 2019; Stuber & Fontaine, 2019). To address this, we summarized mean values from the RCMAP, pinyon‐juniper, cropland, NDVI, and BLM disturbance raster layers within variable buffer sizes. For the RCMAP, pinyon‐juniper, cropland, NDVI, and BLM disturbance covariates, we extracted mean values at 100‐m intervals between 0 m (at the grid centroid) and 10 km from the grid centroid (*n* = 101 buffer sizes; Table 1), to facilitate interpolation between scales and generate a continuous scale parameter (Frishkoff et al., 2019), consistent with similar work (Monroe et al., 2021; Van Lanen et al., 2023a). We note, doing so effectively converted the binary cropland and pinyon‐juniper layers into continuous variables representing the proportion of cells within each buffer distance represented by these landcover types (hereafter, “proportion pinyon‐juniper” and “proportion cropland”). We centered and scaled all extracted covariate values to facilitate model fitting.

We calculated Pearson's pairwise correlations (*r*) among our covariates to limit the inclusion of highly correlated variables in our models. Due to the large number of buffer distances considered, assessing possible correlations for every buffer distance and covariate combination was impractical. Instead, we selected values at 200‐m, 5000‐m, and 10,000‐m buffer extents for covariates for which we assessed spatial scales of effect, to assess correlations among covariates. For correlation tests, we used the centroid (PDSI) or 564‐m buffer values (elevation and vrm) for covariates for which we did not estimate a scale of effect. We removed a covariate from the model when *r* ≥ 0.6 between two variables for two of the three spatial scales assessed. We also calculated variance inflation factors (VIF) using the “car” package (Fox & Weisberg, 2019) for each species' model to ensure VIF < 3.0 (Zuur et al., 2010) and reduce potential multicollinearity issues.

### 2.4 Population model and model fitting

To model avian density‐habitat relationships, we developed a modified hierarchical abundance model combining distance sampling and removal modeling procedures (Amundson et al., 2014) for each species. This model accounts for some individuals that may never sing or perch in a visible location during a count (availability) and assumes the probability of a surveyor detecting an available bird will decline with increasing distance (detectability). For each species, we modeled the summed number of independent detections, d, at grid *g*, in year *t* as

(1) $$d_{\mathrm{gt}}\sim \text{Binomial}\;\left(N_{\mathrm{gt}},{\text{pmarg}}_{\mathrm{gt}}\right)$$

where $N_{\mathrm{gt}}$ represents the true (latent) number of individuals present and ${\text{pmarg}}_{\mathrm{gt}}$ represents the joint probability of an individual being detected, given it was available for detection.

We modeled the number of individuals present in a grid as a zero‐inflated Poisson process influenced by the expected number of individuals, ${\text{lambda}}_{\mathrm{gt}}$, and the probability the grid is within a region where the species was likely to occur, ${\text{Active}}_{g}$.

(2) $$N_{\mathrm{gt}}\sim \text{Poisson}\left({\text{lambda}}_{\mathrm{gt}}*{\text{Active}}_{g}\right)$$

We allowed the mean expected number of individuals ($\mathrm{mu}.{\text{lambda}}_{\mathrm{gt}}$) to vary with a survey‐level (combination of grid and year) random error term, sd.survey:

(3) $${\text{lambda}}_{\mathrm{gt}}\sim \text{Normal}\left(\mathrm{mu}.{\text{lambda}}_{\mathrm{gt}},\mathrm{sd}.\text{survey}\right)$$

Specifically, we modeled $\mathrm{mu}.{\text{lambda}}_{\mathrm{gt}}$ as a function of grid and year‐specific resource conditions using a generalized linear model:

(4) $$\log\left(\mathrm{mu}.{\text{lambda}}_{\mathrm{gt}}\right)=\beta_{0_{r}}+\mathrm{\beta x}+\Gamma *w+\mu_{r}*\left(t-2008\right)+{\text{offset}}_{\mathrm{gt}}$$

Here, $\beta_{0r}$ represents the random intercept for a given BCR, *r*; β represents linear effects of vrm, PDSI, point disturbance features, and both linear and quadratic effects of elevation for a given species; and x represents the covariate values. We included the quadratic term on elevation expecting an upper elevational limit for our species of interest. The Γ parameters corresponded to linear effects of the covariates for which we estimated spatial scale selection parameters: pinyon‐juniper, cropland, annual herbaceous, herbaceous, litter, sagebrush, NDVI, point disturbance, and linear disturbance cover amounts. We note, the spatial scale selection procedures allow for interpolation between the buffer distances to generate continuous scale parameters (see Table 1). We also included a parameter for a quadratic effect of NDVI, based upon the expectation species abundance would decline with high values of NDVI associated with wetlands and dense forests. We multiplied Γ parameters by w, a vector of covariate values corresponding to a buffer extent for each covariate, grid, and year combination which was estimated using procedures developed by Frishkoff et al. (2019). To both estimate and account for regional population trends of each species, which are not accounted for by our time‐varying covariates, we fit a linear trend for years since 2008 for each BCR, $\mu_{r}$. Finally, we included an offset $\left({\text{offset}}_{\mathrm{gt}}\right)$ representing the natural log of the number of point counts conducted during the survey, to account for variable sampling effort across grids and years.

To allow for possible correlations between our random intercept, $\beta_{0r}$, and our random population trend slopes, $\mu_{r}$, we applied a multivariate normal distribution and an inverse Wishart model for the covariance matrix (Σw):

(5) $$\begin{array}{c} \beta_{0_{r}}\sim \text{Multivariate}\;\text{Normal}\;\left(\mathrm{mu}.{\text{wish}}_{r,1},\text{Σ}\text{w}\right) \end{array}$$

(6) $$\begin{array}{c} \mu_{r}\sim \text{Multivariate}\;\text{Normal}\;\left(\mathrm{mu}.{\text{wish}}_{r,2},\mathrm{\Sigma w}\right) \end{array}$$

(7) $$\mathrm{mu}.{\text{wish}}_{r,1:2}\sim \text{Normal}\;\left(0,10,000\right)$$

(8) $$\mathrm{\Sigma w}=\left[\begin{array}{cc} \sigma_{\beta_{0r}}^{2} & \mathrm{cov}\;\left(\beta_{0r},\mu_{r}\right)\\ \mathrm{cov}\;\left(\beta_{0r},\mu_{r}\right) & \sigma_{\mu_{r}}^{2} \end{array}\right]$$

(9) $${\mathrm{\Sigma w}}^{-1}\sim \text{Wishart}\;\left(R,\mathrm{df}\right)$$

where Σw consisted of the variance among BCR‐specific intercepts for each species $\left(\sigma_{\beta_{0r}}^{2}\right)$, variance among the BCR and species‐specific population trends ($\sigma_{\mu_{r}}^{2}$), and the covariance among the intercepts and trend slopes ($\mathrm{cov}\;\left(\beta_{0r},\mu_{r}\right)$). The parameters associated with the inverse Wishart distribution included a scale matrix, *R*, and the degrees of freedom (df; Kéry & Schaub, 2012). We used three degrees of freedom and assigned a prior to *R*, equal to:

$$\left[\begin{array}{cc} 5 & 0\\ 0 & 1 \end{array}\right]$$

We suspected some locations within our large study area may lie outside a particular species' range. To account for this, we incorporated a zero‐inflation component to our model, ${\text{Active}}_{g}.$

(10) $${\text{Active}}_{g}\sim \text{Bernoulli}\;\left(\psi_{g}\right)$$

We followed methods described by Wood (2016) and Monroe et al. (2021) to develop a two‐dimensional thin plate spline. We used the jagam function in the “mgcv” package (Wood, 2016). Doing so allowed the probability of a grid being suitable for the species of interest, $\psi_{g}$, to vary spatially and according to a basis function, $g_{k}$, with *K*−1 dimensions and a smoothing parameter, ω. We used *K* = 100 basis dimensions for all but one species. We used *K* = 150 for Black‐throated Gray Warbler to facilitate chain convergence. We supplied easting and northing coordinates which corresponded to the coordinates of each grid centroid in the basis function.

(11) $$f\left({\text{easting}}_{g},{\text{northing}}_{g}\right)=\sum_{k=1}^{K-1}g_{k}\left({\text{easting}}_{g},{\text{northing}}_{g}\right)\omega$$

(12) $$\text{logit}\left(\psi_{g}\right)=a_{0i}+f\left({\text{easting}}_{g},{\text{northing}}_{g}\right)$$

To account for individuals present, but not detected, we estimated an overall detection probability, ${\text{pmarg}}_{\mathrm{gt}}$ for each species, grid, and year combination. We estimated ${\text{pmarg}}_{\mathrm{gt}}$ as the product of the probability an individual was available to be detected, $p_{a_{\mathrm{gt}}}$, during the point count and the probability the observer would detect the individual, $p_{d_{\mathrm{gt}}}$, provided it was available to be detected during the count (Amundson et al., 2014). We assigned each independent detection, *i*, to a minute interval, ${\text{tint}}_{i}$, and distance band, ${\text{dclass}}_{i}$, and expressed cell probabilities π as a categorical distribution for ${\text{tint}}_{i}$ and ${\text{dclass}}_{i}$.

(13) $${\text{tint}}_{i}\sim \text{Categorical}\;\left(\pi_{a}^{c}\right)$$

(14) $${\text{dclass}}_{i}\sim \text{Categorical}\;\left(\pi_{d}^{c}\right)$$

We used previously developed removal modeling procedures (Farnsworth et al., 2002), informed by the minute interval, *m*, in which each individual was detected to estimate $p_{a_{\mathrm{gy}}}$:

(15) $$\pi_{a_{\mathrm{mgt}}}^{c}=\frac{\pi_{a_{\mathrm{mgt}}}}{p_{a_{\mathrm{gt}}}}$$

Here, $\pi_{a_{\mathrm{mgt}}}^{c}$ represented the probability an individual was available during minute interval *m*, and was calculated as:

(16) $$\pi_{a_{\mathrm{mgt}}}^{c}=a_{\mathrm{gt}}{\left(1-a_{\mathrm{gt}}\right)}^{m-1}$$

where $a_{\mathrm{gt}}$ represented the probability an individual was available during each 1‐min interval. We then calculated the probability an individual would be available for detection during at least 1 min interval by summing the availability probabilities across all minute intervals ($M_{t}$ = 5 in 2008–2009, $M_{t}$ = 6 thereafter).

(17) $$p_{a_{\mathrm{gt}}}=\sum_{m=1}^{M_{y}}\pi_{a_{\mathrm{mgt}}}$$

We suspected the probability an individual would be available for detection during at least a single minute interval of the count to vary seasonally, with individuals being more available early in the summer (when advertising territories and building nests) and again late in the summer (when feeding young). We therefore modeled species availability as

(18) $$\text{logit}\;\left(p_{a_{\mathrm{gt}}}\right)=\alpha +A_{\text{OrdDate}}E_{\mathrm{gt}}$$

influenced by linear and quadratic effects, $A_{x}$, of ordinal date, $E_{\mathrm{gt}}$.

To account for birds which were available for detection but were not detected by the observer, we modeled detectability as a function of distance from the bird to the observer, the observers' experience, and mean start time of all point counts for that survey. To do so, we calculated conditional multinomial cell probabilities, $\pi_{d_{\mathrm{bgt}}}^{c}$, as:

(19) $$\pi_{d_{\mathrm{bgt}}}^{c}=\frac{\pi_{d_{\mathrm{bgt}}}}{\left(p_{d_{\mathrm{gt}}}\right)}$$

where $\pi_{d_{\mathrm{bgt}}}^{c}$ represented the probability an individual bird was detected in distance band *b*, at grid *g*, in year *t*. We estimated $\pi_{d_{\mathrm{bgt}}}^{c}$ using the rectangular rule of approximating the integral and 10 evenly‐spaced distance bins (Kéry & Royle, 2016b). We approximated the integral where the probability distance, *r*, is within a particular bin with bounds, $r_{\mathrm{bg}}$, and bin width, δ.

(20) $$\pi_{r_{\mathrm{bg}}}=\mathrm{Pr}\left(r_{\mathrm{bg}}-\frac{\delta_{p}}{2}\leq r\leq r_{\mathrm{bg}}+\frac{\delta_{p}}{2}\right)\approx g{\left(r\right)}_{\mathrm{bgt}}f{\left(r\right)}_{b}$$

We used the half‐normal distance function, $g{\left(r\right)}_{\mathrm{bgt}}$, to estimate detection as a function of distance:

(21) $$g{\left(r\right)}_{\mathrm{bgt}}=\exp\left(-\frac{r_{b}^{2}}{{2\sigma }_{\mathrm{gt}}^{2}}\right)$$

where $r_{b}$ represented the midpoint of distance bin *b* and $\sigma_{\mathrm{gy}}$ represented the scale factor for the rate at which detection decayed with increasing distance from the observer. We then calculated the probability density function of radial distances from the observer out to the maximum truncation distance, $\max_{d}$.

(22) $$f{\left(r\right)}_{b}=\frac{2r_{b}\delta_{b}}{\mathrm{ma}x_{d}^{2}}$$

We truncated the furthest 10% of all detections for each species to improve estimation of $\sigma_{\mathrm{gt}}$ (Buckland et al., 2001). We modeled heterogeneity of the scale factor using a log‐linear function influenced by observer experience and the mean minutes since sunrise of all counts conducted within grid *g* in year *t*: log ($\sigma_{\mathrm{gt}}$) = τz. We specified priors for model parameters as: β ~ N (0, 10,000), Γ ~ N (0, 10,000), ${Α}_{x}$ ~ N (0, 10,000), τ ~ N (0, 10,000), log (ω) ~ Unif (−12, 12), and *sd.survey* ~ Unif (0, 5).

We conducted our analyses using JAGS 4.3.0 (Plummer, 2003) and the “rjags” package (Plummer, 2019) using four parallel Markov Chain Monte Carlo (MCMC) chains. We ran each species model for 5000 iterations to adapt the MCMC sampler and sampled an additional 120,000 to 650,000 iterations (dependent upon the species). Following initial runs of our full model, we noted poor mixing of the chains associated with the point disturbance covariate for all species. To facilitate chain convergence, we removed the spatial scale selection parameter for this covariate and replaced the covariate values with mean values of point disturbance calculated within a 564‐m radius surrounding each grid centroid, providing point disturbance values which best approximated the 1‐km^2^ grid cell. For several species, we noted poor chain convergence after running 500,000 or more iterations. In these cases, we simplified the model in one of two ways, depending upon the parameters demonstrating poor chain convergence. If the poorly converging parameter was a covariate for which we considered spatial scale selection and/or was the spatial scale selection parameter, we removed the spatial scale selection parameter associated with that covariate and input mean values of the covariate associated with a 564‐m radius surrounding the grid centroid, which again was sufficient to include all point count stations within the grid. If the poorly converging parameter was associated with the random intercept or slope associated with the Bird Conservation Region, we removed all data for BCRs with fewer than 10 detections and re‐ran the model.

When running the final model for each species, we discarded between 10,000 and 500,000 iterations as “burn‐in” and thinned remaining samples sufficient to reserve 500 samples from each of the four chains (total of 2000 samples) for inference regarding chain convergence, model fit, and density‐habitat relationships. We assessed convergence of MCMC chains through visual inspection of traceplots and ensured Gelman‐Rubin potential scale reduction factors (R‐hat; Gelman & Rubin, 1992) were < 1.1 for all parameters. We evaluated model fit using a chi‐square discrepancy posterior predictive check (i.e., Bayesian *p*‐value) (Kéry & Royle, 2016a).

We present the mean and 95% credible intervals (95% CrI) for model parameter estimates of biological interest corresponding to covariates on abundance without scale parameters (*β*), covariates on abundance with scale parameters (Γ), BCR population trends (*μ_r_*), availability covariates (*A*), and covariates on detection (*τ*).

### 2.5 Generating inference and predicted density maps

We evaluated regional trends in songbird populations by calculating the proportion of posterior parameter samples, $\mu_{r}$, which corresponded to a ≥1% annual increase or decrease for each species. We considered regional populations to be increasing or decreasing if ≥90% of samples corresponded to trend values ≥+1% or ≤−1% annual rates of change, respectively. We used the mean parameter estimates associated with covariate‐density relationships to assess expected effects of anthropogenic disturbance (point and linear disturbance) and climate (NDVI and PDSI).

Pinyon‐juniper woodlands are often classified as Phase I (early successional), II (intermediate), and III (mature) woodlands (Miller et al., 2019). Prior research has identified that herbaceous cover, perennial shrub cover, and pinyon‐juniper canopy cover vary predictably with succession from sagebrush to mature pinyon‐juniper woodlands (Miller et al., 2019; Roundy et al., 2014), with sagebrush and herbaceous cover decreasing and pinyon‐juniper cover increasing as communities transition from Phase I to Phase III woodlands. Therefore, we used our modeled covariate relationships for herbaceous, sagebrush, and proportion pinyon‐juniper cover to evaluate which species are most likely to occur within the successional phases occurring within sagebrush and pinyon‐juniper ecotones. We note our pinyon‐juniper cover predictor variable in the model represents the proportion of the surrounding area classified as pinyon‐juniper woodlands, not the percent of pinyon‐juniper canopy cover as is used to classify late successional woodlands. Therefore, our approach assumes large, continuous patches of pinyon‐juniper woodland have higher canopy cover (later successional stages); a pattern we feel is generally appropriate but may not be consistent with conditions on the ground in all cases. Unfortunately, large‐scale raster layers representing pinyon‐juniper woodland phase do not currently exist, prohibiting a direct assessment regarding the influence of successional phase on avian density. We included habitat relationships developed for the Pinyon Jay using the same methodology (Van Lanen et al., 2023a) in our evaluation, as this species is associated with pinyon‐juniper woodlands and of increasing conservation concern.

Based upon the modeled density‐habitat relationships and site characteristics of sagebrush and pinyon‐juniper successional stages (Miller et al., 2019, Roundy et al., 2014), we expected species positively associated with sagebrush cover and negatively associated with the proportion of pinyon‐juniper woodlands to occur at highest densities within sagebrush ecosystems. We expected species positively associated with both the proportion of pinyon‐juniper cover and sagebrush cover to occur at highest densities within early successional pinyon‐juniper woodlands (Phase I and Phase II). Finally, we expect species positively associated with the proportion of pinyon‐juniper cover and negatively associated with herbaceous cover and/or sagebrush cover to occur at highest densities within late successional pinyon‐juniper woodlands (Phase III). As pinyon‐juniper removal for sage‐grouse has largely targeted Phase I and II pinyon‐juniper woodlands (Coates et al., 2017), we expected species occurring at high densities within Phase I and phase II woodlands would be most negatively affected by pinyon‐juniper removal efforts to restore sagebrush. In contrast, species occurring at high densities within sagebrush ecosystems would likely benefit most from pinyon‐juniper removal efforts.

We generated maps of predicted density for each species using layers which best approximated 2020 conditions for each covariate. We resampled raster input layers to 30‐m resolution using bilinear interpolation, as necessary, using the “gdalwarp” package (gdalwarp, 2021) in the OSGeo4W64 Shell software (OSGeo4W, 2021). Again using “gdalwarp,” we reprojected each raster layer into Albers Conical Equal Area projection. We generated moving window rasters using Python (PyCharm Community Edition, 2020), representing mean raster values for each covariate with radii equal to the mode of the posterior distribution for each spatial scale selection parameter, or a 564‐m radius when we did not allow the spatial scale to vary.

We calculated median predicted species‐specific densities (number of birds per 1 km^2^) by dividing the number of predicted individuals by *ᴨ* * *r* ^2^, where r represents the 90% truncated detection distance for each species (Buckland et al., 2001). We calculated median predicted densities for each pixel using the 2000 saved posterior samples and the above‐described moving window raster layers. We masked predicted density maps where values of the point disturbance layer exceeded values used to fit the model (some raster pixel values exceeded 130 standard deviations above the mean model inputs). We also developed a quality assurance layer in which we masked out pixels in our predicted density raster layers whenever one or more of the moving window raster values fell outside of the 2.5 and 97.5% quantiles of values used to train the model. We provide predicted density maps and masking layers generated during this study as a USGS data release (Van Lanen et al., 2023b).

## 3 RESULTS

The highest Pearson's pairwise correlation value among covariates ranged from 0.496 to 0.605 for our species and no species had correlation values above 0.6 for more than a single tested scale. We therefore retained all covariates when modeling each species' abundance. Multicollinearity was not an issue in our data set, with the highest VIFs for our species <3.0 (range: 2.055–2.395). Results of our chi‐square discrepancy posterior predictive checks generally indicated adequate fit of our models, with Bayesian *p*‐values ranging from .091 to 0.381. We estimated Bayesian *p*‐values <.15 for Brewer's Sparrow (*p* = .09) and Sage Thrasher (*p* = .13).

The number of grids and surveys included in the data sets varied by species and ranged from 1101 to 1434 and from 7390 to 9682, respectively (Table 2). Brewer's Sparrow (*n* = 45,805 independent detections), Green‐tailed Towhee (*n* = 21,203), and Sage Thrasher (*n* = 13,209) were the most detected species in our data set, while Gray Vireo (*n* = 1391), Juniper Titmouse (*n* = 1386), and Loggerhead Shrike (*n* = 749) were the least detected (Table 2).

**TABLE 2:** Number of grids, surveys, and species' detections by Bird Conservation Region used in the modeling of avian abundance across the InterMountain West, 2008–2020.

Bird Conservation Region	Species	# Detections
9	# Grids = 201	# Surveys = 855
	Bewick's Wren	131
	Black‐throated Gray Warbler	592
	Brewer's Sparrow	10,629
	Gray Flycatcher	896
	Gray Vireo	194
	Green‐tailed Towhee	1730
	Juniper Titmouse	245
	Loggerhead Shrike	192
	Sage Thrasher	2533
	Sagebrush Sparrow	1327
	Townsend's Solitaire	48
10	# Grids = 525	# Surveys = 4029
	Bewick's Wren	1025
	Black‐throated Gray Warbler	218
	Brewer's Sparrow	26,059
	Gray Flycatcher	703
	Gray Vireo	30
	Green‐tailed Towhee	11,231
	Juniper Titmouse	132
	Loggerhead Shrike	186
	Sage Thrasher	9876
	Sagebrush Sparrow	7526
	Townsend's Solitaire	2586
11	# Grids = 32	# Surveys = 230
	Brewer's Sparrow	812
	Loggerhead Shrike	22
	Sage Thrasher	22
15	# Grids = 5	# Surveys = 17
	Bewick's Wren	34
	Brewer's Sparrow	15
	Green‐tailed Towhee	110
	Townsend's Solitaire	16
16	# Grids = 375	# Surveys = 2506
	Bewick's Wren	1152
	Black‐throated Gray Warbler	3667
	Brewer's Sparrow	3397
	Gray Flycatcher	1666
	Gray Vireo	1134
	Green‐tailed Towhee	8025
	Juniper Titmouse	918
	Loggerhead Shrike	80
	Sage Thrasher	600
	Sagebrush Sparrow	609
	Townsend's Solitaire	1966
17	# Grids = 194	# Surveys = 1268
	Brewer's Sparrow	4539
	Gray Flycatcher	17
	Green‐tailed Towhee	81
	Loggerhead Shrike	179
	Sage Thrasher	169
	Townsend's Solitaire	2779
18	# Grids = 89	# Surveys = 721
	Bewick's Wren	86
	Brewer's Sparrow	336
	Gray Flycatcher	1
	Gray Vireo	1
	Green‐tailed Towhee	26
	Juniper Titmouse	19
	Loggerhead Shrike	65
	Sage Thrasher	2
	Townsend's Solitaire	1
33	# Grids = 13	# Surveys = 56
	Bewick's Wren	52
	Black‐throated Gray Warbler	40
	Brewer's Sparrow	18
	Gray Flycatcher	10
	Gray Vireo	30
	Juniper Titmouse	20
	Loggerhead Shrike	25
	Sage Thrasher	7
34	# Grids = 2	# Surveys = 11
	Bewick's Wren	50
	Black‐throated Gray Warbler	91
	Gray Flycatcher	70
	Gray Vireo	2
	Juniper Titmouse	52

### 3.1 Influence of detectability and availability

**FIGURE 2:**
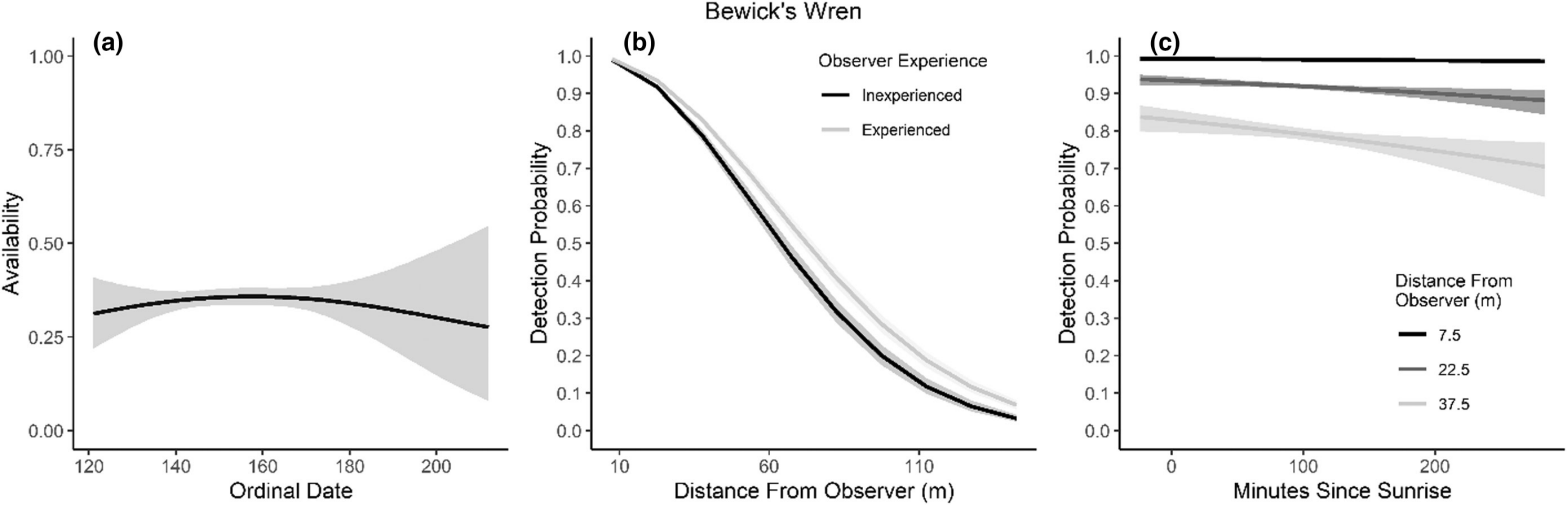
Predicted influence of ordinal date on availability (a), observer experience on detection probability (b), and mean minutes since sunrise on detection probability (c) for Bewick's Wren during breeding season point counts, 2008–2020.

**FIGURE 3:**
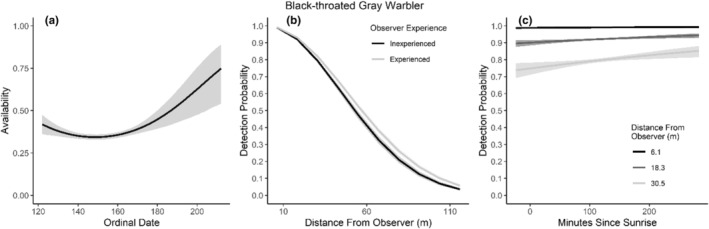
Modeled influence of ordinal date on availability (a), observer experience on detection probability (b), and mean minutes since sunrise on detection probability (c) for Black‐throated Gray Warbler during breeding season point counts, 2008–2020.

**FIGURE 4:**
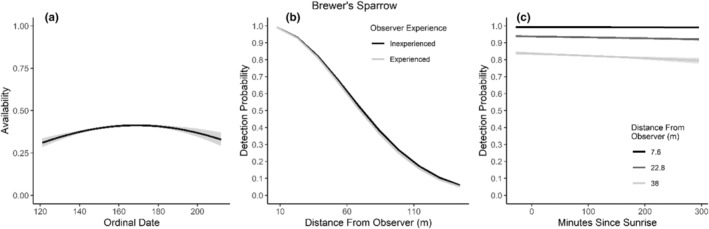
Modeled influence of ordinal date on availability (a), observer experience on detection probability (b), and mean minutes since sunrise on detection probability (c) for Brewer's Sparrow during breeding season point counts, 2008–2020.

**FIGURE 5:**
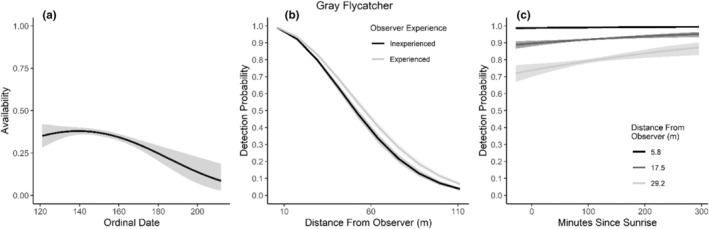
Modeled influence of ordinal date on availability (a), observer experience on detection probability (b), and mean minutes since sunrise on detection probability (c) for Gray Flycatcher during breeding season point counts, 2008–2020.

**FIGURE 6:**
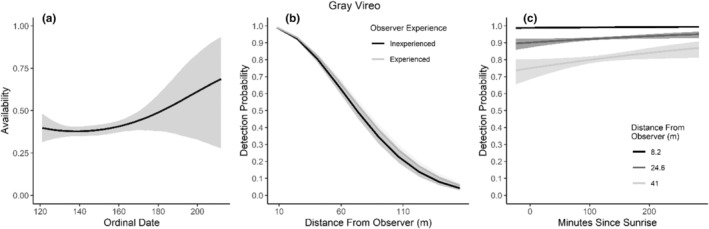
Modeled influence of ordinal date on availability (a), observer experience on detection probability (b), and mean minutes since sunrise on detection probability (c) for Gray Vireo during breeding season point counts, 2008–2020.

**FIGURE 7:**
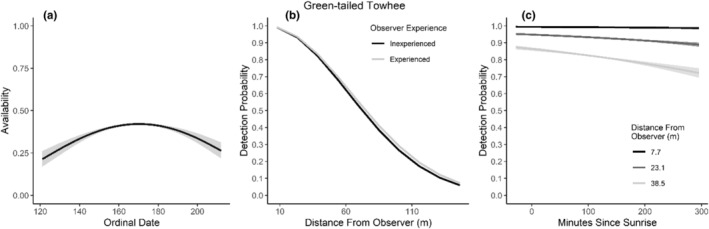
Modeled influence of ordinal date on availability (a), observer experience on detection probability (b), and mean minutes since sunrise on detection probability (c) for Green‐tailed Towhee during breeding season point counts, 2008–2020.

**FIGURE 8:**
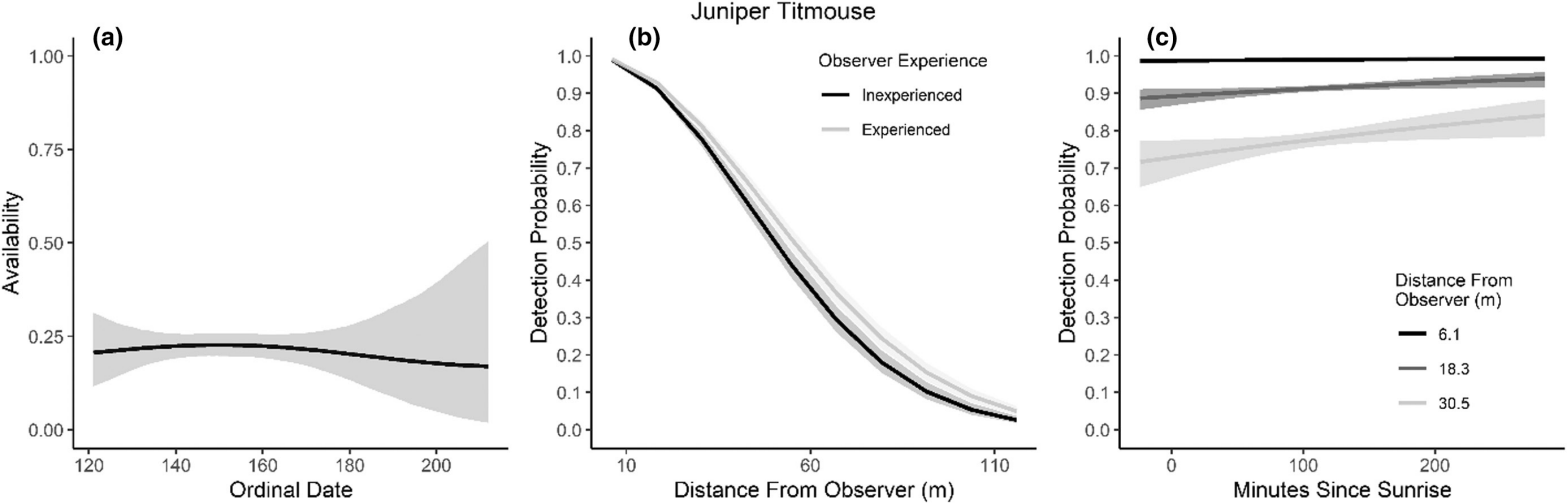
Modeled influence of ordinal date on availability (a), observer experience on detection probability (b), and mean minutes since sunrise on detection probability (c) for Juniper Titmouse during breeding season point counts, 2008–2020.

**FIGURE 9:**
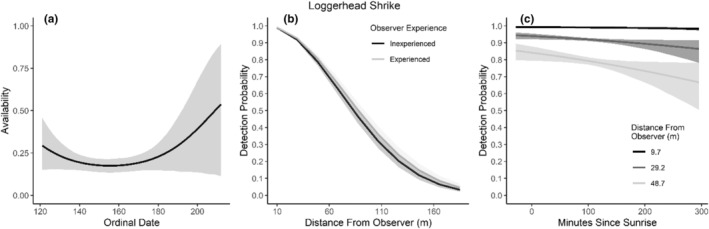
Modeled influence of ordinal date on availability (a), observer experience on detection probability (b), and mean minutes since sunrise on detection probability (c) for Loggerhead Shrike during breeding season point counts, 2008–2020.

**FIGURE 10:**
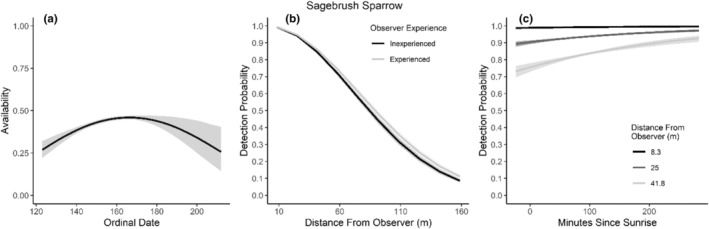
Modeled influence of ordinal date on availability (a), observer experience on detection probability (b), and mean minutes since sunrise on detection probability (c) for Sagebrush Sparrow during breeding season point counts, 2008–2020.

**FIGURE 11:**
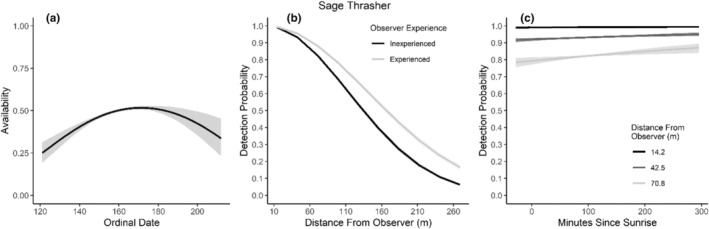
Modeled influence of ordinal date on availability (a), observer experience on detection probability (b), and mean minutes since sunrise on detection probability (c) for Sage Thrasher during breeding season point counts, 2008–2020.

**FIGURE 12:**
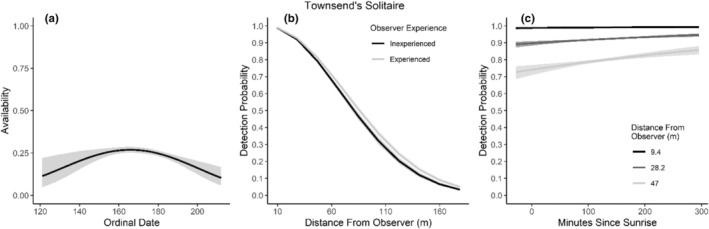
Modeled influence of ordinal date on availability (a), observer experience on detection probability (b), and mean minutes since sunrise on detection probability (c) for Townsend's Solitaire during breeding season point counts, 2008–2020.

We found availability for detection varied throughout the summer breeding season for seven species (Table 3; Figures 2, 3, 4, 5, 6, 7, 8, 9, 10, 11, 12). Black‐throated Gray Warbler availability increased while Gray Flycatcher decreased during the season. Availability was greatest at intermediate dates for Brewer's Sparrow, Green‐tailed Towhee, Sagebrush Sparrow, Sage Thrasher, and Townsend's Solitaire (Table 3; Figures 2, 3, 4, 5, 6, 7, 8, 9, 10, 11, 12).

**TABLE 3:** Mean parameter estimates and associated 95% lower (LCrI) and upper (UCrI) credible intervals for predictive variable influences on availability and detectability for songbird species in the InterMountain West.

Species	Observation process	Parameter	Mean	LCrI	UCrI
Bewick's Wren	Availability	Ordinal Date	−0.048	−0.250	0.137
		Ordinal Date^2^	−0.047	−0.171	0.066
	Detection	Observer Experience	**0.123**	**0.072**	**0.173**
		Mean Minutes Since Sunrise	**−0.043**	**−0.074**	**−0.011**
Black‐throated Gray Warbler	Availability	Ordinal Date	**0.287**	**0.124**	**0.444**
		Ordinal Date^2^	**0.122**	**0.045**	**0.194**
	Detection	Observer Experience	**0.075**	**0.038**	**0.111**
		Mean Minutes Since Sunrise	**0.040**	**0.013**	**0.065**
Brewer's Sparrow	Availability	Ordinal Date	0.020	−0.002	0.042
		Ordinal Date^2^	**−0.056**	**−0.077**	**−0.034**
	Detection	Observer Experience	**−0.025**	**−0.038**	**−0.013**
		Mean Minutes Since Sunrise	**−0.017**	**−0.026**	**−0.007**
Gray Flycatcher	Availability	Ordinal Date	**−0.341**	**−0.508**	**−0.182**
		Ordinal Date^2^	**−0.110**	**−0.196**	**−0.025**
	Detection	Observer Experience	**0.097**	**0.054**	**0.139**
		Mean Minutes Since Sunrise	**0.053**	**0.021**	**0.082**
Gray Vireo	Availability	Ordinal Date	0.250	−0.057	0.543
		Ordinal Date^2^	0.076	−0.064	0.207
	Detection	Observer Experience	0.061	−0.009	0.129
		Mean Minutes Since Sunrise	**0.049**	**0.005**	**0.091**
Green‐tailed Towhee	Availability	Ordinal Date	**0.057**	**0.020**	**0.093**
		Ordinal Date^2^	**−0.117**	**−0.153**	**−0.082**
	Detection	Observer Experience	**0.035**	**0.017**	**0.053**
		Mean Minutes Since Sunrise	**−0.054**	**−0.066**	**−0.040**
Juniper Titmouse	Availability	Ordinal Date	−0.098	−0.418	0.205
		Ordinal Date^2^	−0.051	−0.219	0.112
	Detection	Observer Experience	**0.098**	**0.036**	**0.155**
		Mean Minutes Since Sunrise	**0.041**	**0.006**	**0.077**
Loggerhead Shrike	Availability	Ordinal Date	0.190	−0.147	0.488
		Ordinal Date^2^	0.156	−0.037	0.336
	Detection	Observer Experience	0.056	−0.019	0.132
		Mean Minutes Since Sunrise	**−0.058**	**−0.109**	**−0.008**
Sagebrush Sparrow	Availability	Ordinal Date	−0.029	−0.130	0.074
		Ordinal Date^2^	**−0.119**	**−0.178**	**−0.060**
	Detection	Observer Experience	**0.055**	**0.024**	**0.086**
		Mean Minutes Since Sunrise	**0.088**	**0.064**	**0.112**
Sage Thrasher	Availability	Ordinal Date	**0.087**	**0.033**	**0.143**
		Ordinal Date^2^	**−0.132**	**−0.190**	**−0.075**
	Detection	Observer Experience	**0.215**	**0.188**	**0.241**
		Mean Minutes Since Sunrise	**0.034**	**0.011**	**0.056**
Townsend's Solitaire	Availability	Ordinal Date	−0.005	−0.110	0.106
		Ordinal Date^2^	**−0.160**	**−0.262**	**−0.059**
	Detection	Observer Experience	**0.061**	**0.028**	**0.093**
		Mean Minutes Since Sunrise	**0.045**	**0.025**	**0.066**

*Note*: Parameter values estimated from a Bayesian hierarchical population model informed by point count data collected from 2008 to 2020. Parameters for which the credible intervals do not overlap zero are shown in bold.

Detectability was influenced by mean minutes since sunrise when point counts were conducted for all 11 of the species we investigated (Table 3; Figures 2, 3, 4, 5, 6, 7, 8, 9, 10, 11, 12). Detectability within a given distance bin declined later in the morning for five species (Bewick's Wren, Brewer's Sparrow, Green‐tailed Towhee, Juniper Titmouse, and Loggerhead Shrike) and increased for six of the species investigated (Black‐throated Gray Warbler, Gray Flycatcher, Gray Vireo, Sagebrush Sparrow, and Sage Thrasher). Experienced observers were more likely to detect eight species within a given distance bin compared to inexperienced observers (Bewick's Wren, Black‐throated Gray Warbler, Gray Flycatcher, Green‐tailed Towhee, Juniper Titmouse, Sagebrush Sparrow, Sage Thrasher, and Townsend's Solitaire). Inexperienced observers detected Brewer's Sparrow more frequently within a given distance bin than experienced observers (Table 3; Figures 2, 3, 4, 5, 6, 7, 8, 9, 10, 11, 12).

### 3.2 Population trends

**FIGURE 13:**
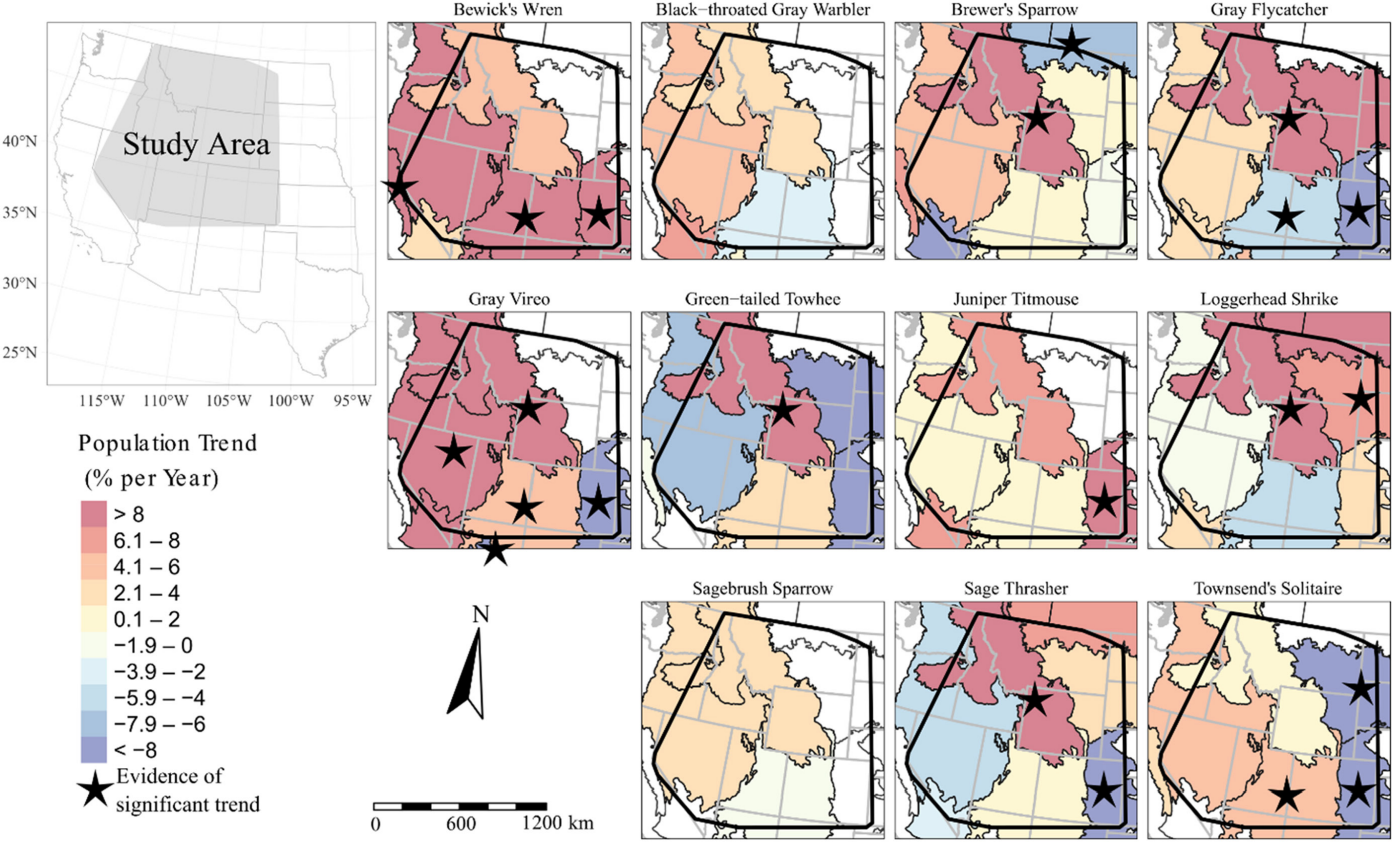
Annual population trends (% change per year) within Bird Conservation Regions for 12 songbird species based upon hierarchical Bayesian density‐habitat relationship models within the western United States of America; 2008–2020. Stars on the map indicate there was ≥90% probability the population is increasing or decreasing by ≥1% within the Bird Conservation Region. We omitted displaying trend significance within BCR15 (extreme western portion of study area) for Bewick's Wren and BCR34 for Gray Vireo from the figure for clarity. Population trends for Pinyon Jay are from Van Lanen et al. (2023a). Bases modified from National Weather Service, 1:2,000,000, 1980 and from Bird Studies Canada and NABCI, 2014 digital data.

We found strong evidence (≥90% probability) of annual population trends exceeding ±1% per year for 22 species‐BCR combinations from 2008 to 2020 (Figure 13; Table 4). Of these species‐BCR combinations, 8 indicated declining populations while 14 indicated increasing populations. All but two species, Black‐throated Gray Warbler and Sagebrush Sparrow, increased or decreased within ≥1 BCR during the timeframe of our study. Gray Vireo populations increased within the Great Basin region (BCR9). We estimated six species increasing within the Northern Rockies region (BCR10; Brewer's Sparrow, Gray Flycatcher, Gray Vireo, Green‐tailed Towhee, Loggerhead Shrike, and Sage Thrasher) and found no evidence of decreasing trends within this region. Brewer's Sparrow populations declined within the Prairie Potholes region (BCR11) and Bewick's Wren populations increased in the Sierra Nevada region (BCR15). We estimated three species increasing within the Southern Rockies (BCR16) region (Bewick's Wren, Gray Vireo, and Townsend's Solitaire) whereas Gray Flycatcher densities declined. Loggerhead Shrike populations increased while Townsend's Solitaire decreased within the Badlands and Prairies (BCR17) region. We estimated population declines for Gray Flycatcher, Gray Vireo, Sage Thrasher, and Townsend's Solitaire while Bewick's Wren and Juniper Titmouse increased within the Shortgrass Prairie (BCR18) region. Lastly, Gray Vireo populations declined within the Chihuahuan Desert (BCR34) region (Figure 13; Table 4).

**TABLE 4:** Mean parameter estimates and associated 95% lower (LCrI) and upper (UCrI) credible intervals for intercept and trend parameters.

Species	Parameter	BCR	Mean	LCrI	UCrI	Pr (1% Δ)
Bewick's Wren	Intercept	9	−6.372	−10.037	−3.684	
		10	−3.057	−3.917	−2.257	
		15	−4.777	−9.438	−1.367	
		16	−4.346	−5.123	−3.621	
		18	−3.365	−4.963	−1.815	
		33	−3.075	−6.264	0.996	
		34	−3.989	−6.974	−1.146	
	Trend	9	0.154	−0.108	0.486	82.5%
		10	0.048	−0.031	0.127	84.3%
		**15**	**0.322**	**−0.015**	**0.765**	**96.8%**
		**16**	**0.083**	**0.021**	**0.146**	**99.2%**
		**18**	**0.242**	**0.054**	**0.429**	**99.3%**
		33	0.027	−0.353	0.355	56.9%
		34	0.098	−0.347	0.537	65.6%
Black‐throated Gray Warbler	Intercept	9	−2.428	−3.623	−1.328	
		10	−2.505	−3.246	−1.836	
		16	−1.449	−1.787	−1.106	
		33	−2.448	−5.419	−0.039	
		34	−1.727	−3.618	0.177	
	Trend	9	0.045	−0.073	0.177	70.8%
		10	0.029	−0.052	0.113	66.1%
		16	−0.027	−0.059	0.004	85.8%
		33	0.059	−0.21	0.369	62.8%
		34	0.008	−0.317	0.339	49.7%
Brewer's Sparrow	Intercept	9	−0.473	−1.136	0.168	
		10	−1.009	−1.243	−0.774	
		11	0.726	0.027	1.478	
		15	−0.972	−3.699	1.703	
		16	−1.053	−1.413	−0.691	
		17	−0.293	−0.698	0.1	
		18	−2.818	−3.381	−2.286	
		33	−1.071	−3.719	1.528	
	Trend	9	0.042	−0.022	0.107	83.4%
		**10**	**0.107**	**0.084**	**0.13**	**100.0%**
		**11**	**−0.079**	**−0.168**	**0.001**	**95.2%**
		15	0.059	−0.211	0.331	64.3%
		16	0.006	−0.033	0.045	44.3%
		17	0.017	−0.027	0.059	63.1%
		18	−0.003	−0.071	0.066	42.1%
		33	−0.1	−0.364	0.159	76.0%
Gray Flycatcher	Intercept	9	−3.295	−4.632	−1.958	
		10	−4.239	−5.039	−3.48	
		16	−2.658	−3.257	−2.101	
		17	−4.128	−6.114	−2.309	
		18	−4.697	−6.871	−2.706	
		33	−4.059	−6.95	−1.509	
		34	−3.236	−5.464	−0.99	
	Trend	9	0.037	−0.094	0.165	65.7%
		**10**	**0.112**	**0.038**	**0.188**	**99.6%**
		**16**	**−0.049**	**−0.093**	**−0.001**	**94.5%**
		17	0.137	−0.091	0.387	85.7%
		**18**	**−0.632**	**−1.61**	**−0.058**	**98.5%**
		33	0.028	−0.229	0.323	53.1%
		34	0.051	−0.292	0.362	60.3%
Gray Vireo	Intercept	9	−5.371	−7.913	−3.025	
		10	−7.031	−9.504	−4.811	
		16	−4.307	−5.154	−3.49	
		18	−5.377	−8.132	−3.021	
		33	−5.173	−8.32	−1.943	
		34	−4.511	−7.711	−0.206	
	Trend	**9**	**0.173**	**−0.044**	**0.403**	**93.8%**
		**10**	**0.295**	**0.069**	**0.54**	**99.4%**
		**16**	**0.051**	**−0.002**	**0.108**	**93.3%**
		**18**	**−0.533**	**−1.486**	**0.071**	**94.8%**
		33	0.137	−0.17	0.44	81.3%
		**34**	**−0.491**	**−1.408**	**0.129**	**92.1%**
Green‐tailed Towhee	Intercept	9	−0.914	−2.086	0.334	
		10	−2.344	−2.673	−2.015	
		15	−0.522	−3.602	2.519	
		16	−1.36	−1.711	−1.036	
		17	1.262	−0.862	3.577	
		18	−0.656	−2.007	0.672	
	Trend	9	−0.083	−0.208	0.036	88.7%
		**10**	**0.084**	**0.055**	**0.115**	**100.0%**
		15	−0.003	−0.317	0.293	48.2%
		16	0.021	−0.009	0.052	75.9%
		17	−0.155	−0.409	0.095	87.1%
		18	−0.097	−0.27	0.075	83.3%
Juniper Titmouse	Intercept	9	−3.177	−4.797	−1.495	
		10	−3.735	−4.732	−2.777	
		16	−3.276	−3.851	−2.725	
		18	−3.741	−5.313	−2.293	
		33	−3.838	−6.585	−1.355	
		34	−3.161	−5.18	−1.012	
	Trend	9	0.002	−0.158	0.157	46.6%
		10	0.06	−0.042	0.164	84.3%
		16	0	−0.038	0.04	31.5%
		**18**	**0.171**	**−0.034**	**0.382**	**93.8%**
		33	0.067	−0.17	0.337	68.4%
		34	0.017	−0.294	0.312	53.5%
Loggerhead Shrike	Intercept	9	−3.845	−5.028	−2.673	
		10	−4.341	−5.258	−3.491	
		11	−4.511	−6.146	−2.898	
		16	−3.397	−4.428	−2.406	
		17	−4.054	−4.89	−3.267	
		18	−5.052	−6.011	−4.113	
		33	−3.959	−6.152	−1.787	
	Trend	9	−0.015	−0.121	0.087	52.7%
		**10**	**0.079**	**0.005**	**0.147**	**96.8%**
		11	0.093	−0.076	0.258	84.2%
		16	−0.053	−0.158	0.058	79.2%
		**17**	**0.061**	**−0.014**	**0.136**	**90.9%**
		18	0.034	−0.054	0.124	70.2%
		33	0.027	−0.184	0.251	55.5%
Sagebrush Sparrow	Intercept	9	−4.151	−5.41	−2.93	
		10	−3.865	−4.472	−3.246	
		16	−4.447	−5.269	−3.646	
	Trend	9	0.029	−0.092	0.164	60.6%
		10	0.033	−0.009	0.076	85.3%
		16	−0.007	−0.096	0.078	47.0%
Sage Thrasher	Intercept	9	−1.964	−2.814	−1.123	
		10	−3.05	−3.426	−2.669	
		11	−3.559	−5.171	−2.036	
		16	−3.125	−3.775	−2.511	
		17	−4.485	−5.32	−3.667	
		18	−3.758	−6.086	−1.383	
		33	−4.049	−7.194	−1.623	
	Trend	9	−0.051	−0.134	0.026	84.4%
		**10**	**0.088**	**0.057**	**0.117**	**100.0%**
		11	0.068	−0.109	0.251	72.9%
		16	0.002	−0.067	0.071	41.1%
		17	0.027	−0.065	0.121	63.6%
		**18**	**−0.758**	**−1.572**	**−0.21**	**99.8%**
		33	−0.027	−0.282	0.276	58.2%
Townsend's Solitaire	Intercept	9	−3.096	−4.896	−1.517	
		10	−1.928	−2.165	−1.69	
		15	−1.891	−4.463	1.107	
		16	−2.293	−2.618	−1.975	
		17	−1.938	−2.573	−1.286	
		18	−4.339	−6.928	−2.191	
	Trend	9	0.053	−0.104	0.229	68.6%
		10	0.015	−0.008	0.04	68.3%
		15	0.029	−0.262	0.288	57.4%
		**16**	**0.04**	**0.01**	**0.069**	**97.7%**
		**17**	**−0.13**	**−0.205**	**−0.057**	**99.9%**
		**18**	**−0.487**	**−1.016**	**−0.047**	**98.3%**

*Note*: The probability of a 1% or more annual change in population (Pr[1% Δ]) is provided for trend estimates. Probabilities of annual change provided correspond to increases when the Mean is positive and decreases when the Mean is negative. Results are shown by Bird Conservation Region (BCR) from 2008 to 2020.

Trend parameters for which the credible intervals do not overlap zero are shown in bold.

### 3.3 Density‐habitat relationships

**FIGURE 14:**
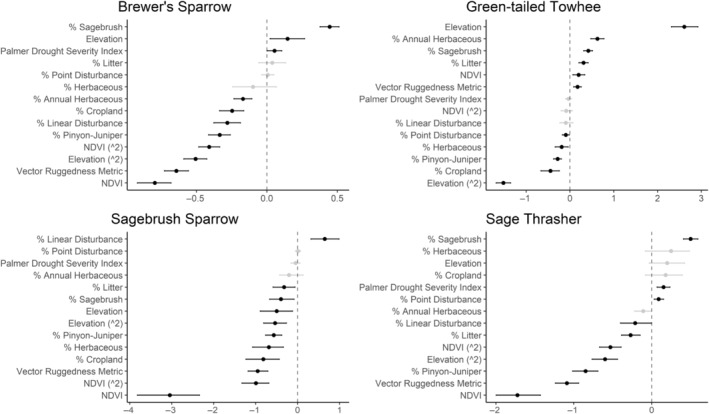
Point estimates (dots) and associated 95% credible intervals (whiskers) for Bayesian hierarchical model parameters influencing avian density of four sagebrush‐associated songbird species in the western United States of America; 2008–2020. Whisker coloration indicates if the 95% credible interval for each parameter estimate does (gray) or does not (black) overlap zero.

**FIGURE 15:**
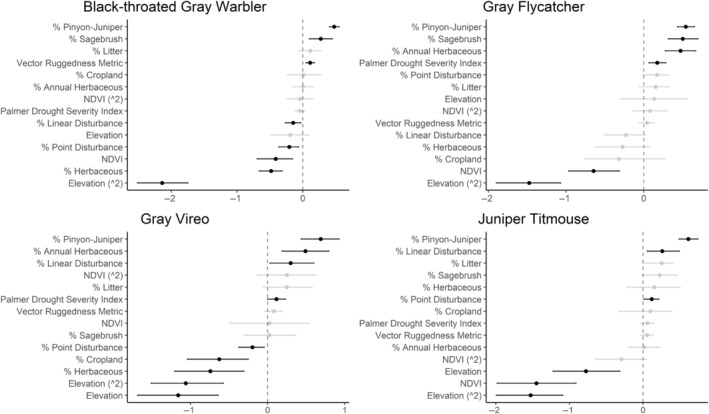
Point estimates (dots) and associated 95% credible intervals (whiskers) for Bayesian hierarchical model parameters influencing avian density of four pinyon‐juniper associated songbird species in the western United States of America; 2008–2020. Whisker coloration indicates if the 95% credible interval for each parameter estimate does (gray) or does not (black) overlap zero.

**FIGURE 16:**
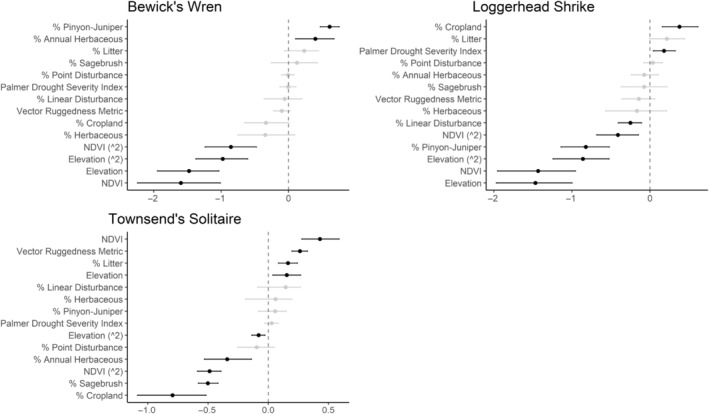
Point estimates (dots) and associated 95% credible intervals (whiskers) for Bayesian hierarchical model parameters influencing avian density of three generalist songbird species in the western United States of America; 2008–2020. Whisker coloration indicates if the 95% credible interval for each parameter estimate does (gray) or does not (black) overlap zero.

We successfully estimated spatial scales at which species respond to predictor variables for all 11 species, however, we were unable to estimate scales of effect for all combinations of species and predictor variables. Table 5 provides the estimated scales of effect for each species and covariate combination and indicates which covariates we were unable to estimate a scale of effect (Table 5). Landcover variables resulted in varied effects for the generalist, sagebrush, and pinyon‐juniper associated species in our study (Figures 14, 15, 16; Table 6). We found support for associations between avian density and annual herbaceous cover (*n* = 6 species), proportion of cropland (*n* = 6), herbaceous cover (*n* = 4), litter cover (*n* = 4), proportion of pinyon‐juniper (*n* = 10), and sagebrush cover (*n* = 7) (Table 6). Herbaceous cover was not positively associated with songbird densities for any of the 11 species we investigated, but we identified four species with negative associations, including 2 sagebrush‐associated and 2 pinyon‐juniper‐associated species. Black‐throated Gray Warbler and Sagebrush Sparrow demonstrated strong negative associations while Gray Vireo and Green‐tailed Towhee densities were only slightly and negatively associated with herbaceous cover. Both sagebrush‐ and pinyon‐juniper‐associated species were positively associated with sagebrush cover. Brewer's Sparrow, Black‐throated Gray Warblers, Gray Flycatcher, and Sage Thrasher were strongly and positively associated with increasing sagebrush cover while Green‐tailed Towhee demonstrated a modest positive association with sagebrush (Figures 14, 15, 16; Table 6). We estimated a strong negative association between sagebrush cover and density for one generalist species (Townsend's Solitaire) and a weak but negative a with one sagebrush associated species (Sagebrush Sparrow). Our model results indicated the proportion of pinyon‐juniper cover was associated with densities of 10 of the 11 species (Figures 14, 15, 16; Table 6). Pinyon‐juniper cover was strongly and positively associated with one generalist (Bewick's Wren) and three pinyon‐juniper associated species (Black‐throated Gray Warbler, Gray Flycatcher, and Juniper Titmouse). Pinyon‐juniper cover was weakly and positively associated with another pinyon‐juniper‐associated species (Gray Vireo). Conversely, pinyon‐juniper cover was strongly and negatively associated with two species characterized as sagebrush associates: Brewer's Sparrow and Sage Thrasher. One generalist species (Loggerhead Shrike) and two sagebrush‐associated species (Green‐tailed Towhee and Sagebrush Sparrow) demonstrated weakly negative associations with pinyon‐juniper cover (Figures 14, 15, 16; Table 6).

**TABLE 5:** Spatial scales in meters, based upon the mode of the posterior distribution, corresponding to parameter estimates for density‐habitat relationship models for 11 songbird species occurring within sagebrush and pinyon‐juniper ecotones; 2008–2020.

Species	A. Herb^a^	Crop^b^	Herb^a^	Litter^a^	PJ^c^	Sage^a^	NDVI^d^	Lin. Dist^e^	BCRs^f^
Bewick's Wren	9350	313	3731	136	625	651	93	7158	9, 10, 15, 16, 18, 33, 34
Black‐throated Gray Warblers	564*	564*	239	254	600	9509	6313	3367	9, 10, 16, 33, 34
Brewer's Sparrow	637	1128	948	8826	591	564*	141	3232	9, 10, 11, 15, 16, 17, 18, 33
Gray Flycatcher	6812	208	3513	257	645	4768	4280	6857	9, 10, 16, 17, 18, 33, 34
Gray Vireo	6585	2097	564*	2252	9775	6899	9124	2016	9, 10, 16, 18, 33, 34
Green‐tailed Towhee	9784	325	9341	564*	1248	2913	1820	3549	9, 10, 15, 16, 17, 18
Juniper Titmouse	8832	6215	1637	17	589	2342	3143	1749	9, 10, 16, 18, 33, 34
Loggerhead Shrike	2600	9520	3151	6090	1027	9099	2105	286	9, 10, 11, 16, 17, 18, 33
Sagebrush Sparrow	694	697	9245	9659	498	9746	1044	9548	9, 10, 16
Sage Thrasher	4699	9352	9582	3272	1246	1026	582	3169	9, 10, 11, 16, 17, 18, 33
Townsend's Solitaire	5207	2074	9247	400	709	523	800	9557	9, 10, 15, 16, 17, 18

*Note*: Spatial scales demarcated with a (*) were not estimable and were therefore calculated at 564‐m radius surrounding grid centroids.

^a^ Annual herbaceous cover (A. Herb), herbaceous cover (Herb), litter cover (Litter), and sagebrush cover (Sage) derived from Rangeland condition and monitoring assessment and projection products (Rigge et al., 2021).

^b^ The proportion of pixels representing cropland cover, derived from a binary raster layer developed from reclassifying National Cropscape data (United States Department of Agriculture, 2008–2020).

^c^ The proportion of pixels representing pinyon‐juniper cover, derived from binary raster layers developed by reclassifying LANDFIRE existing vegetation types (LANDFIRE, 2008, 2010, 2012, 2014, 2016).

^d^ Normalized Difference Vegetation Index (NDVI; Didan, 2015), derived by calculating maximum NDVI during summer months.

^e^ Proportion of cells containing at least one linear disturbance feature, derived from unpublished data (Bureau of Land Management).

^f^ Bird Conservation Regions for which population trends were estimated (BCR; United States North American Bird Conservation Initiative Committee, 2000).

**TABLE 6:** Mean, 95% lower credible interval (LCrI), and 95% upper credible interval (UCrI) estimates for parameters influencing modeled hierarchical habitat‐density relationships for 11 songbird species occurring in the sagebrush and pinyon‐juniper ecotone; USA 2008–2020.

Species	Parameter	Mean	LCrI	UCrI
Bewick's Wren	Annual Herbaceous Cover^a^	**0.398**	**0.105**	**0.677**
	Cropland Cover^b^	−0.335	−0.655	0.007
	Elevation^c^	**−1.477**	**−1.945**	**−1.033**
	Elevation (^2)^c^	**−0.977**	**−1.375**	**−0.605**
	Herbaceous Cover^a^	−0.345	−0.752	0.092
	Proportion Linear Disturbance^d^	−0.057	−0.364	0.199
	Litter Cover^a^	0.233	−0.058	0.444
	NDVI^e^	**−1.597**	**−2.242**	**−1.012**
	NDVI (^2)^e^	**−0.856**	**−1.238**	**−0.477**
	Proportion Point Disturbance^d^	−0.007	−0.102	0.086
	PDSI^f^	−0.007	−0.127	0.111
	Proportion of Pinyon‐Juniper Cover^g^	**0.610**	**0.471**	**0.752**
	Sagebrush Cover^a^	0.125	−0.254	0.428
	Vector Ruggedness^h^	−0.100	−0.220	0.022
Black‐throated Gray Warbler	Annual Herbaceous Cover^a^	0.004	−0.156	0.169
	Cropland Cover^b^	0.011	−0.233	0.273
	Elevation^c^	−0.192	−0.481	0.090
	Elevation (^2)^c^	**−2.138**	**−2.513**	**−1.747**
	Herbaceous Cover^a^	**−0.482**	**−0.664**	**−0.311**
	Proportion Linear Disturbance^d^	**−0.146**	**−0.266**	**−0.027**
	Litter Cover^a^	0.117	−0.064	0.277
	NDVI^e^	**−0.412**	**−0.695**	**−0.155**
	NDVI (^2)^e^	−0.042	−0.236	0.151
	Proportion Point Disturbance^d^	**−0.206**	**−0.365**	**−0.064**
	PDSI^f^	−0.045	−0.118	0.027
	Proportion of Pinyon‐Juniper Cover^g^	**0.476**	**0.404**	**0.552**
	Sagebrush Cover^a^	**0.273**	**0.101**	**0.448**
	Vector Ruggedness^h^	**0.111**	**0.046**	**0.176**
Brewer's Sparrow	Annual Herbaceous Cover^a^	**−0.170**	**−0.234**	**−0.107**
	Cropland Cover^b^	**−0.247**	**−0.335**	**−0.164**
	Elevation^c^	**0.146**	**0.023**	**0.268**
	Elevation (^2)^c^	**−0.506**	**−0.589**	**−0.426**
	Herbaceous Cover^a^	−0.099	−0.242	0.068
	Proportion Linear Disturbance^d^	**−0.281**	**−0.377**	**−0.188**
	Litter Cover^a^	0.038	−0.057	0.133
	NDVI^e^	**−0.795**	**−0.919**	**−0.681**
	NDVI (^2)^e^	**−0.410**	**−0.484**	**−0.335**
	Proportion Point Disturbance^d^	0.006	−0.037	0.049
	PDSI^f^	**0.054**	**0.001**	**0.105**
	Proportion of Pinyon‐Juniper Cover^g^	**−0.335**	**−0.414**	**−0.261**
	Sagebrush Cover^a^	**0.445**	**0.377**	**0.512**
	Vector Ruggedness^h^	**−0.643**	**−0.728**	**−0.557**
Gray Flycatcher	Annual Herbaceous Cover^a^	**0.474**	**0.277**	**0.670**
	Cropland Cover^b^	−0.319	−0.753	0.272
	Elevation^c^	0.135	−0.298	0.562
	Elevation (^2)^c^	**−1.475**	**−1.899**	**−1.070**
	Herbaceous Cover^a^	−0.272	−0.624	0.073
	Proportion Linear Disturbance^d^	−0.230	−0.509	0.022
	Litter Cover^a^	0.155	−0.054	0.330
	NDVI^e^	**−0.646**	**−0.967**	**−0.313**
	NDVI (^2)^e^	0.081	−0.135	0.295
	Proportion Point Disturbance^d^	0.171	−0.007	0.318
	PDSI^f^	**0.174**	**0.066**	**0.285**
	Proportion of Pinyon‐Juniper Cover^g^	**0.541**	**0.435**	**0.653**
	Sagebrush Cover^a^	**0.502**	**0.317**	**0.698**
	Vector Ruggedness^h^	0.041	−0.057	0.138
Gray Vireo	Annual Herbaceous Cover^a^	**0.491**	**0.188**	**0.794**
	Cropland Cover^b^	**−0.626**	**−1.039**	**−0.249**
	Elevation^c^	**−1.158**	**−1.685**	**−0.639**
	Elevation (^2)^c^	**−1.059**	**−1.506**	**−0.572**
	Herbaceous Cover^a^	**−0.742**	**−1.203**	**−0.306**
	Proportion Linear Disturbance^d^	**0.300**	**0.032**	**0.600**
	Litter Cover^a^	0.249	−0.052	0.578
	NDVI^e^	0.025	−0.489	0.543
	NDVI (^2)^e^	0.252	−0.132	0.624
	Proportion Point Disturbance^d^	**−0.196**	**−0.373**	**−0.044**
	PDSI^f^	**0.114**	**0.001**	**0.233**
	Proportion of Pinyon‐Juniper Cover^g^	**0.689**	**0.434**	**0.928**
	Sagebrush Cover^a^	0.025	−0.305	0.368
	Vector Ruggedness^h^	0.079	−0.035	0.192
Green‐tailed Towhee	Annual Herbaceous Cover^a^	**0.626**	**0.467**	**0.783**
	Cropland Cover^b^	**−0.445**	**−0.658**	**−0.241**
	Elevation^c^	**2.612**	**2.313**	**2.931**
	Elevation (^2)^c^	**−1.522**	**−1.688**	**−1.357**
	Herbaceous Cover^a^	**−0.190**	**−0.334**	**−0.040**
	Proportion Linear Disturbance^d^	−0.094	−0.229	0.072
	Litter Cover^a^	**0.310**	**0.205**	**0.423**
	NDVI^e^	**0.199**	**0.071**	**0.343**
	NDVI (^2)^e^	−0.086	−0.199	0.054
	Proportion Point Disturbance^d^	**−0.095**	**−0.171**	**−0.014**
	PDSI^f^	−0.034	−0.096	0.030
	Proportion of Pinyon‐Juniper Cover^g^	**−0.282**	**−0.375**	**−0.196**
	Sagebrush Cover^a^	**0.418**	**0.319**	**0.514**
	Vector Ruggedness^h^	**0.174**	**0.091**	**0.258**
Juniper Titmouse	Annual Herbaceous Cover^a^	0.013	−0.196	0.229
	Cropland Cover^b^	0.098	−0.326	0.394
	Elevation^c^	**−0.776**	**−1.223**	**−0.317**
	Elevation (^2)^c^	**−1.527**	**−1.995**	**−1.088**
	Herbaceous Cover^a^	0.153	−0.218	0.505
	Proportion Linear Disturbance^d^	**0.261**	**0.059**	**0.494**
	Litter Cover^a^	0.254	−0.006	0.415
	NDVI^e^	**−1.448**	**−1.981**	**−0.910**
	NDVI (^2)^e^	−0.293	−0.646	0.055
	Proportion Point Disturbance^d^	**0.118**	**0.010**	**0.218**
	PDSI^f^	0.059	−0.027	0.150
	Proportion of Pinyon‐Juniper Cover^g^	**0.616**	**0.488**	**0.746**
	Sagebrush Cover^a^	0.226	−0.010	0.472
	Vector Ruggedness^h^	0.054	−0.028	0.134
Loggerhead Shrike	Annual Herbaceous Cover^a^	−0.074	−0.243	0.108
	Cropland Cover^b^	**0.376**	**0.158**	**0.614**
	Elevation^c^	**−1.468**	**−1.969**	**−0.998**
	Elevation (^2)^c^	**−0.859**	**−1.243**	**−0.523**
	Herbaceous Cover^a^	−0.167	−0.568	0.214
	Proportion Linear Disturbance^d^	**−0.251**	**−0.405**	**−0.108**
	Litter Cover^a^	0.215	−0.007	0.445
	NDVI^e^	**−1.433**	**−1.953**	**−0.954**
	NDVI (^2)^e^	**−0.411**	**−0.682**	**−0.146**
	Proportion Point Disturbance^d^	0.036	−0.081	0.160
	PDSI^f^	**0.179**	**0.042**	**0.323**
	Proportion of Pinyon‐Juniper Cover^g^	**−0.822**	**−1.139**	**−0.521**
	Sagebrush Cover^a^	−0.076	−0.368	0.221
	Vector Ruggedness^h^	−0.144	−0.359	0.064
Sagebrush Sparrow	Annual Herbaceous Cover^a^	−0.207	−0.425	0.130
	Cropland Cover^b^	**−0.818**	**−1.233**	**−0.436**
	Elevation^c^	**−0.498**	**−0.890**	**−0.119**
	Elevation (^2)^c^	**−0.537**	**−0.810**	**−0.266**
	Herbaceous Cover^a^	**−0.685**	**−1.075**	**−0.341**
	Proportion Linear Disturbance^d^	**0.646**	**0.317**	**0.989**
	Litter Cover^a^	**−0.323**	**−0.583**	**−0.061**
	NDVI^e^	**−3.048**	**−3.819**	**−2.339**
	NDVI (^2)^e^	**−0.993**	**−1.327**	**−0.686**
	Proportion Point Disturbance^d^	0.010	−0.054	0.070
	PDSI^f^	−0.049	−0.153	0.056
	Proportion of Pinyon‐Juniper Cover^g^	**−0.568**	**−0.764**	**−0.377**
	Sagebrush Cover^a^	**−0.397**	**−0.666**	**−0.084**
	Vector Ruggedness^h^	**−0.953**	**−1.180**	**−0.709**
Sage Thrasher	Annual Herbaceous Cover^a^	−0.108	−0.218	0.000
	Cropland Cover^b^	0.180	−0.078	0.394
	Elevation^c^	0.198	−0.031	0.424
	Elevation (^2)^c^	**−0.598**	**−0.764**	**−0.438**
	Herbaceous Cover^a^	0.249	−0.082	0.485
	Proportion Linear Disturbance^d^	**−0.211**	**−0.398**	**−0.002**
	Litter Cover^a^	**−0.271**	**−0.387**	**−0.147**
	NDVI^e^	**−1.723**	**−1.995**	**−1.428**
	NDVI (^2)^e^	**−0.530**	**−0.668**	**−0.391**
	Proportion Point Disturbance^d^	**0.090**	**0.034**	**0.153**
	PDSI^f^	**0.153**	**0.071**	**0.235**
	Proportion of Pinyon‐Juniper Cover^g^	**−0.848**	**−1.014**	**−0.689**
	Sagebrush Cover^a^	**0.500**	**0.408**	**0.594**
	Vector Ruggedness^h^	**−1.089**	**−1.236**	**−0.936**
Townsend's Solitaire	Annual Herbaceous Cover^a^	**−0.341**	**−0.530**	**−0.140**
	Cropland Cover^b^	**−0.796**	**−1.086**	**−0.516**
	Elevation^c^	**0.154**	**0.038**	**0.269**
	Elevation (^2)^c^	**−0.080**	**−0.137**	**−0.026**
	Herbaceous Cover^a^	0.061	−0.190	0.197
	Proportion Linear Disturbance^d^	0.144	−0.088	0.268
	Litter Cover^a^	**0.164**	**0.084**	**0.243**
	NDVI^e^	**0.429**	**0.279**	**0.589**
	NDVI (^2)^e^	**−0.487**	**−0.589**	**−0.391**
	Proportion Point Disturbance^d^	−0.096	−0.254	0.050
	PDSI^f^	0.028	−0.031	0.084
	Proportion of Pinyon‐Juniper Cover^g^	0.058	−0.080	0.151
	Sagebrush Cover^a^	**−0.501**	**−0.581**	**−0.417**
	Vector Ruggedness^h^	**0.263**	**0.196**	**0.327**

*Note*: Parameter values in which the credible interval does not overlap zero are bolded.

^a^ Derived from Rangeland condition and monitoring assessment and projection products (Rigge et al., 2021).

^b^ Derived from a binary raster layer developed from reclassifying National Cropscape (United States Department of Agriculture, 2008–2020).

^c^ United States Department of Agriculture (USDA) Natural Resources Conservation Service (2007).

^d^ Unpublished data (Bureau of Land Management).

^e^ Derived by calculating maximum normalized difference vegetation index during summer months (Didan, 2015).

^f^ National Centers for Environmental Information (2020).

^g^ Derived from binary rasters layer developed by reclassifying LANDFIRE existing vegetation types (LANDFIRE, 2008, 2010, 2012, 2014, 2016).

^h^ O'Donnell et al. (2019).

**FIGURE 17:**
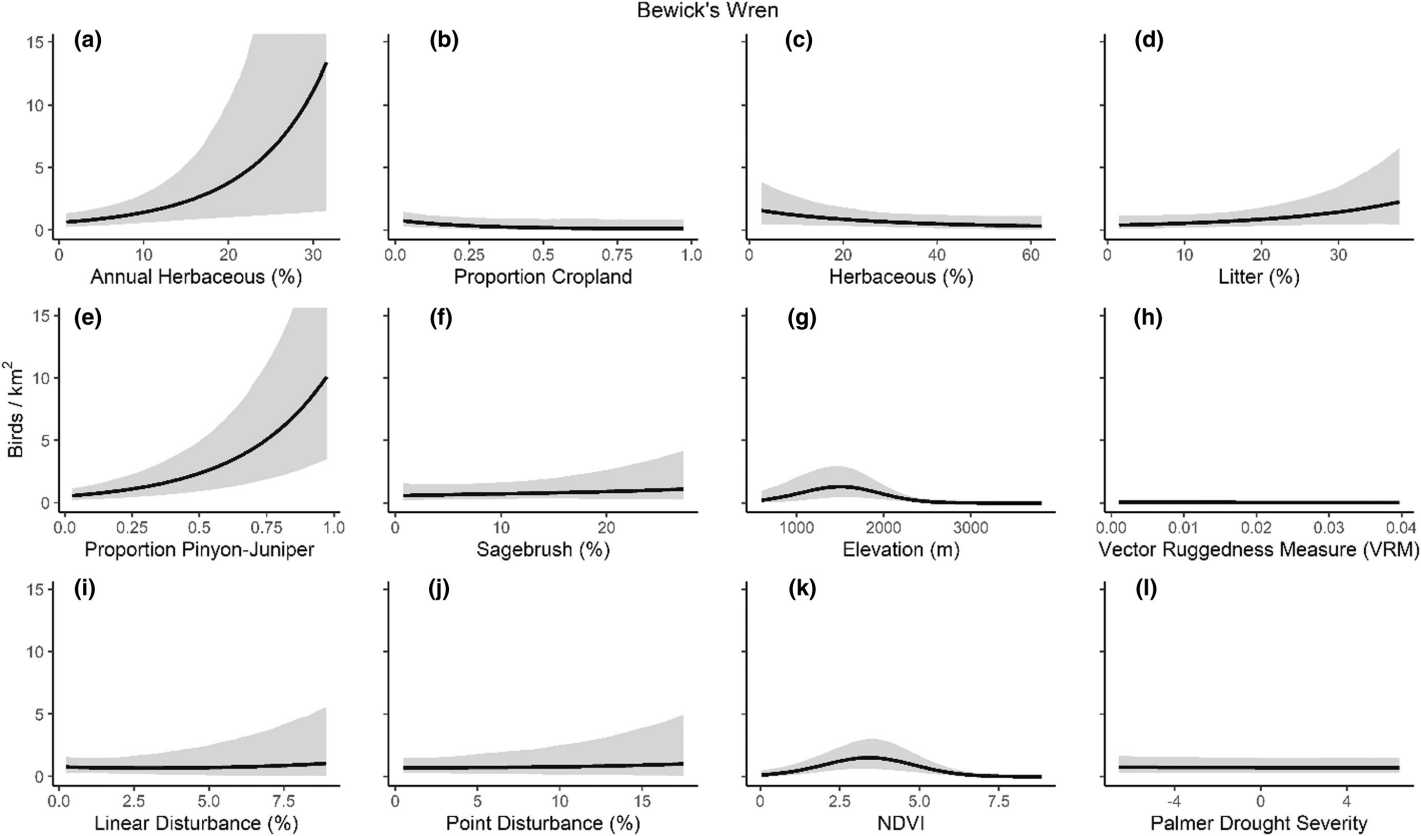
Bewick's Wren mean density (birds/km^2^; line) and 95% credible intervals (ribbon) predicted as a function of covariate values within Bird Conservation Region 10. Relationships were modeled with a Bayesian hierarchical density‐habitat relationship model using point count data collected in the western United States; 2008–2020. Density was calculated by varying each covariate of interest while inputting mean values of all other covariates. Covariate values in panels a, c, d, and f were summarized using RCMAP data (Rigge et al., 2021). Cropland values (panel b) were derived from a binary raster layer developed using reclassified National Cropscape data (United States Department of Agriculture 2008–2020). Pinyon‐juniper cover values (panel e) were derived from binary rasters layer developed by reclassifying LANDFIRE existing vegetation types (LANDFIRE, 2008, 2010, 2012, 2014, 2016). Elevation values (panel g) were extracted at sample grid centroids from a national elevation data set (United States Department of Agriculture (USDA) Natural Resources Conservation Service, 2007). Vector Ruggedness Measures (panel h) were summarized from a product developed by O'Donnell et al. (2019). Linear and Point Disturbance values (panels i and j) were extracted from products developed by the Bureau of Land Management (Bureau of Land Management, Unpublished data). NDVI values (panel k) were derived by calculating means of maximum normalized difference vegetation index values for each pixel during the summer months (Didan, 2015). Palmer Drought Severity Index values (panel l; National Centers for Environmental Information, 2020) were extracted at the grid centroid.

**FIGURE 18:**
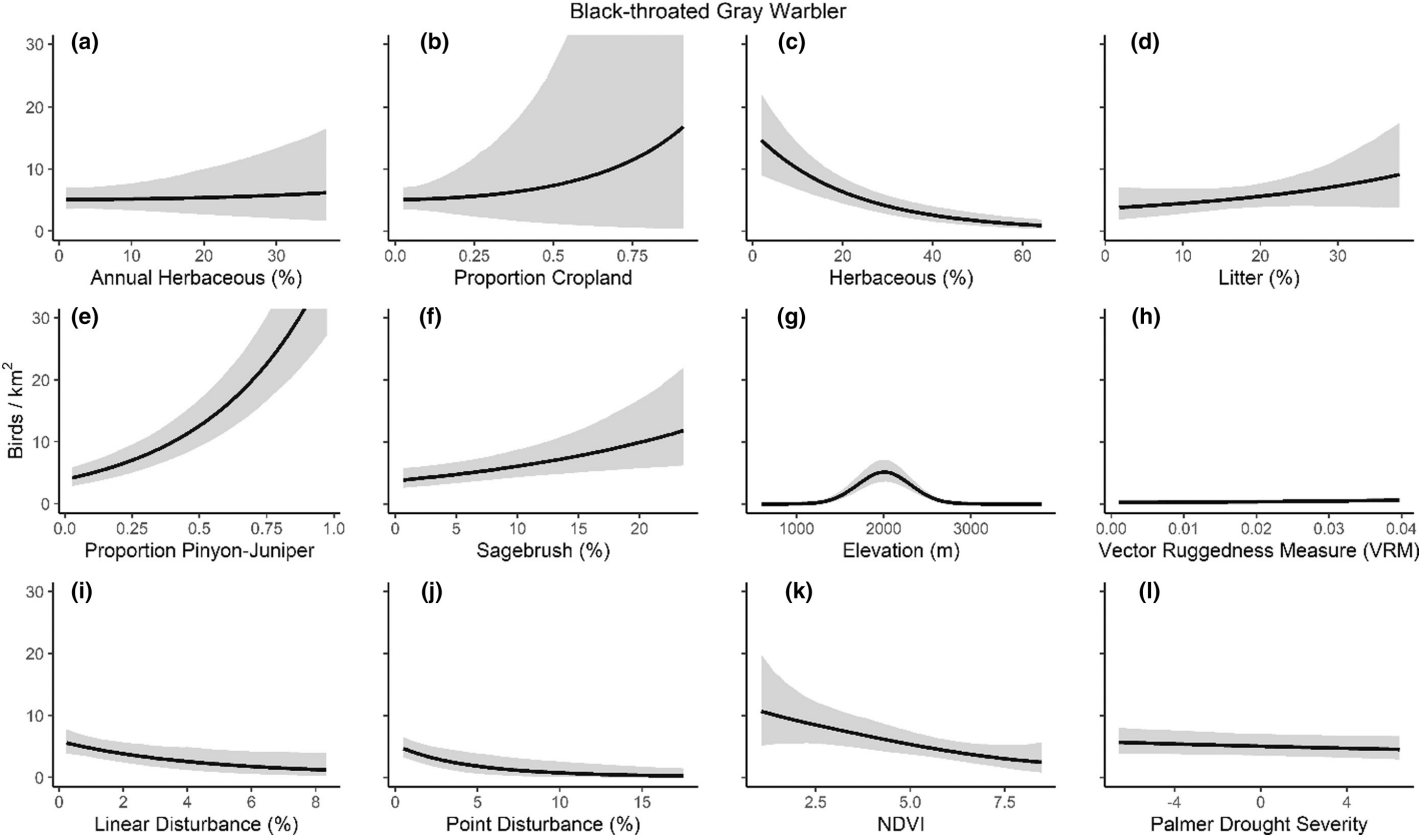
Black‐throated Gray Warbler mean density (birds/km^2^; line) and 95% credible intervals (ribbon) predicted as a function of covariate values within Bird Conservation Region 16. Relationships were modeled with a Bayesian hierarchical density‐habitat relationship model using point count data collected in the western United States; 2008–2020. Density was calculated by varying each covariate of interest while inputting mean values of all other covariates. Covariate values in panels a, c, d, and f were summarized using RCMAP data (Rigge et al., 2021). Cropland values (panel b) were derived from a binary raster layer developed using reclassified National Cropscape data (United States Department of Agriculture 2008–2020). Pinyon‐juniper cover values (panel e) were derived from binary rasters layer developed by reclassifying LANDFIRE existing vegetation types (LANDFIRE, 2008, 2010, 2012, 2014, 2016). Elevation values (panel g) were extracted at sample grid centroids from a national elevation data set (United States Department of Agriculture (USDA) Natural Resources Conservation Service, 2007). Vector Ruggedness Measures (panel h) were summarized from a product developed by O'Donnell et al. (2019). Linear and Point Disturbance values (panels i and j) were extracted from products developed by the Bureau of Land Management (Bureau of Land Management, Unpublished data). NDVI values (panel k) were derived by calculating means of maximum normalized difference vegetation index values for each pixel during the summer months (Didan, 2015). Palmer Drought Severity Index values (panel l; National Centers for Environmental Information, 2020) were extracted at the grid centroid.

**FIGURE 19:**
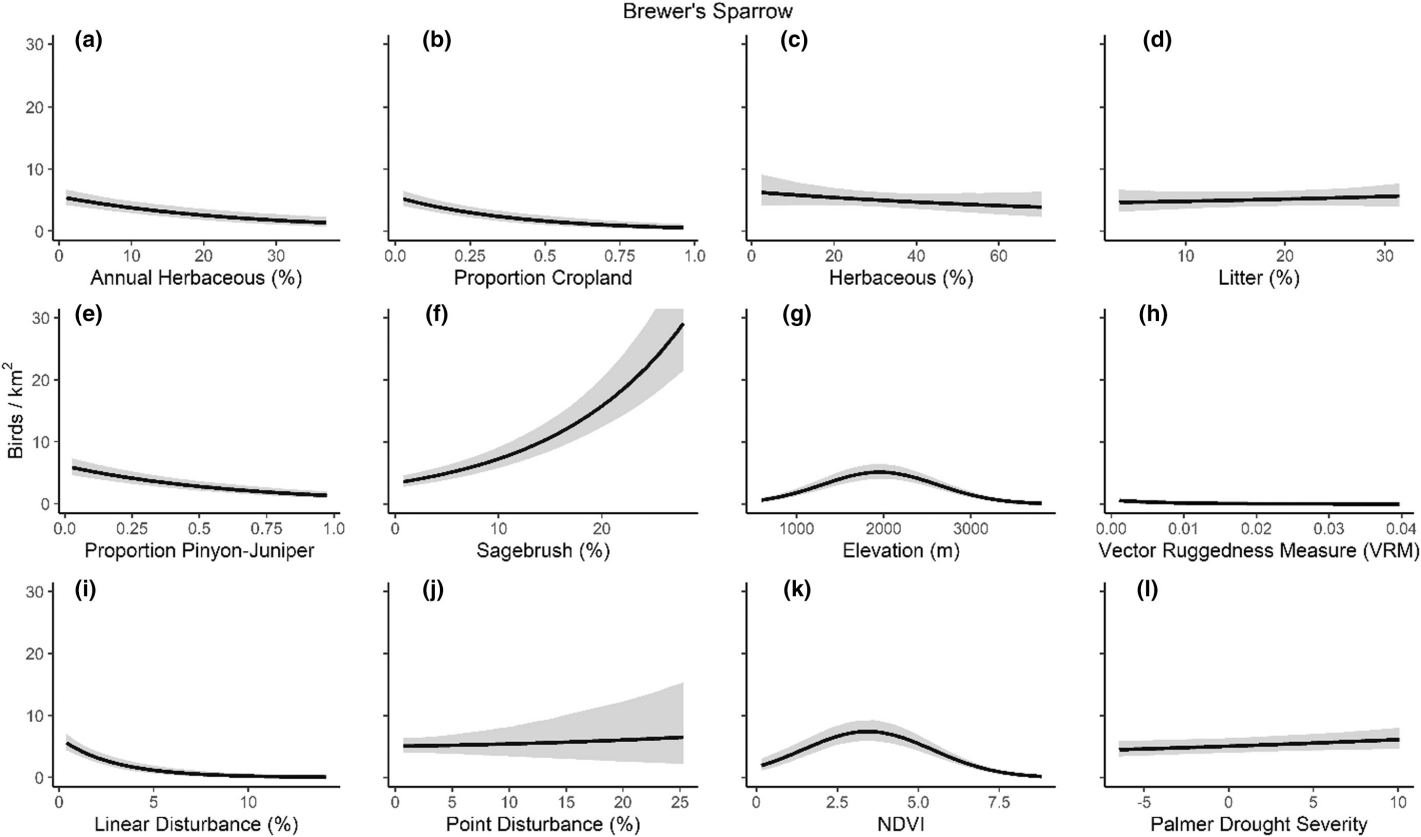
Brewer's Sparrow mean density (birds/km^2^; line) and 95% credible intervals (ribbon) predicted as a function of covariate values within Bird Conservation Region 10. Relationships were modeled with a Bayesian hierarchical density‐habitat relationship model using point count data collected in the western United States; 2008–2020. Density was calculated by varying each covariate of interest while inputting mean values of all other covariates. Covariate values in panels a, c, d, and f were summarized using RCMAP data (Rigge et al., 2021). Cropland values (panel b) were derived from a binary raster layer developed using reclassified National Cropscape data (United States Department of Agriculture, 2008–2020). Pinyon‐juniper cover values (panel e) were derived from binary rasters layer developed by reclassifying LANDFIRE existing vegetation types (LANDFIRE, 2008, 2010, 2012, 2014, 2016). Elevation values (panel g) were extracted at sample grid centroids from a national elevation data set (United States Department of Agriculture (USDA) Natural Resources Conservation Service, 2007). Vector Ruggedness Measures (panel h) were summarized from a product developed by O'Donnell et al. (2019). Linear and Point Disturbance values (panels i and j) were extracted from products developed by the Bureau of Land Management (Bureau of Land Management, Unpublished data). NDVI values (panel k) were derived by calculating means of maximum normalized difference vegetation index values for each pixel during the summer months (Didan, 2015). Palmer Drought Severity Index values (panel l; National Centers for Environmental Information, 2020) were extracted at the grid centroid.

**FIGURE 20:**
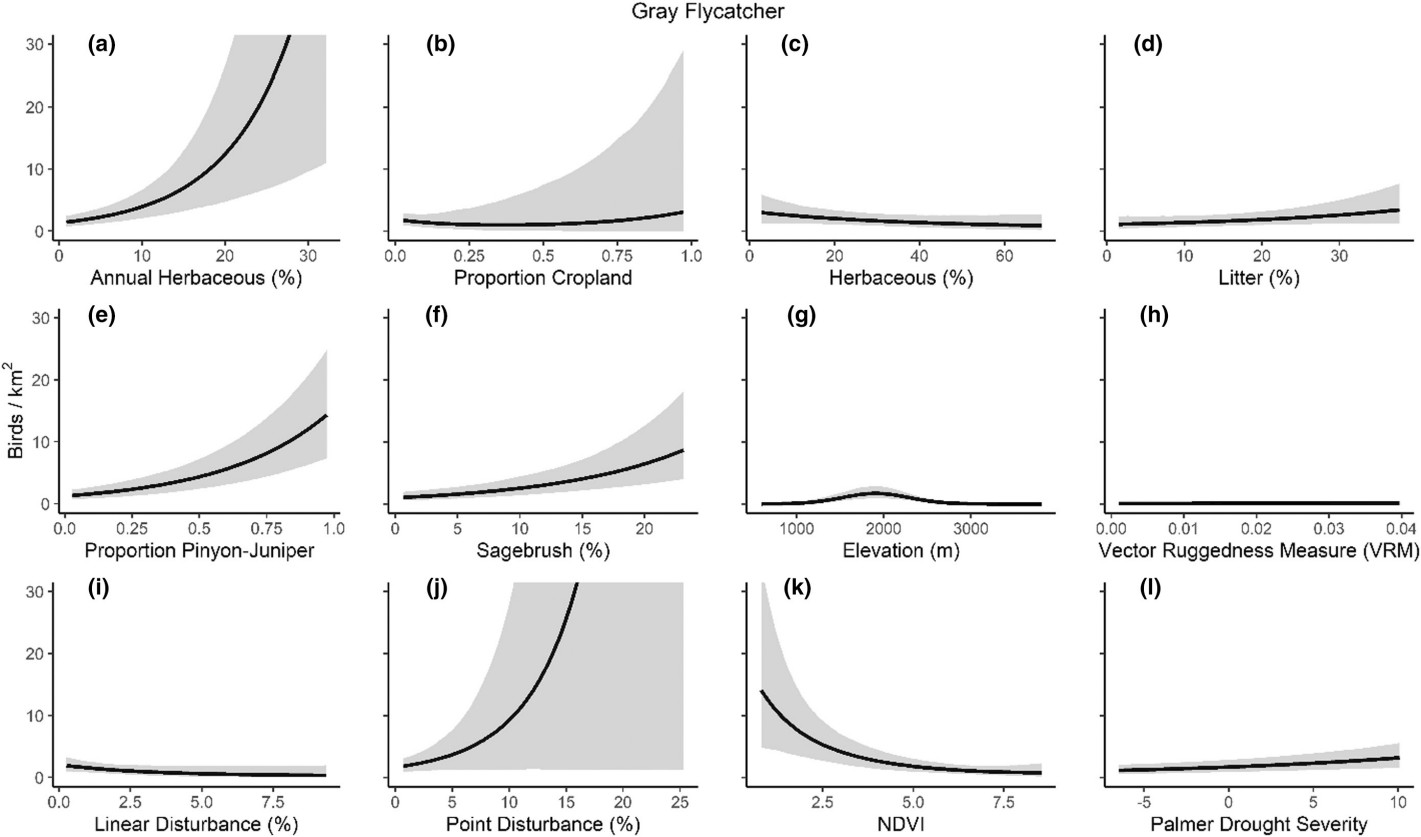
Gray Flycatcher mean density (birds/km^2^; line) and 95% credible intervals (ribbon) predicted as a function of covariate values within Bird Conservation Region 16. Relationships were modeled with a Bayesian hierarchical density‐habitat relationship model using point count data collected in the western United States; 2008–2020. Density was calculated by varying each covariate of interest while inputting mean values of all other covariates. Covariate values in panels a, c, d, and f were summarized using RCMAP data (Rigge et al., 2021). Cropland values (panel b) were derived from a binary raster layer developed using reclassified National Cropscape data (United States Department of Agriculture, 2008–2020). Pinyon‐juniper cover values (panel e) were derived from binary rasters layer developed by reclassifying LANDFIRE existing vegetation types (LANDFIRE, 2008, 2010, 2012, 2014, 2016). Elevation values (panel g) were extracted at sample grid centroids from a national elevation data set (United States Department of Agriculture (USDA) Natural Resources Conservation Service, 2007). Vector Ruggedness Measures (panel h) were summarized from a product developed by O'Donnell et al. (2019). Linear and Point Disturbance values (panels i and j) were extracted from products developed by the Bureau of Land Management (Bureau of Land Management, Unpublished data). NDVI values (panel k) were derived by calculating means of maximum normalized difference vegetation index values for each pixel during the summer months (Didan, 2015). Palmer Drought Severity Index values (panel l; National Centers for Environmental Information, 2020) were extracted at the grid centroid.

**FIGURE 21:**
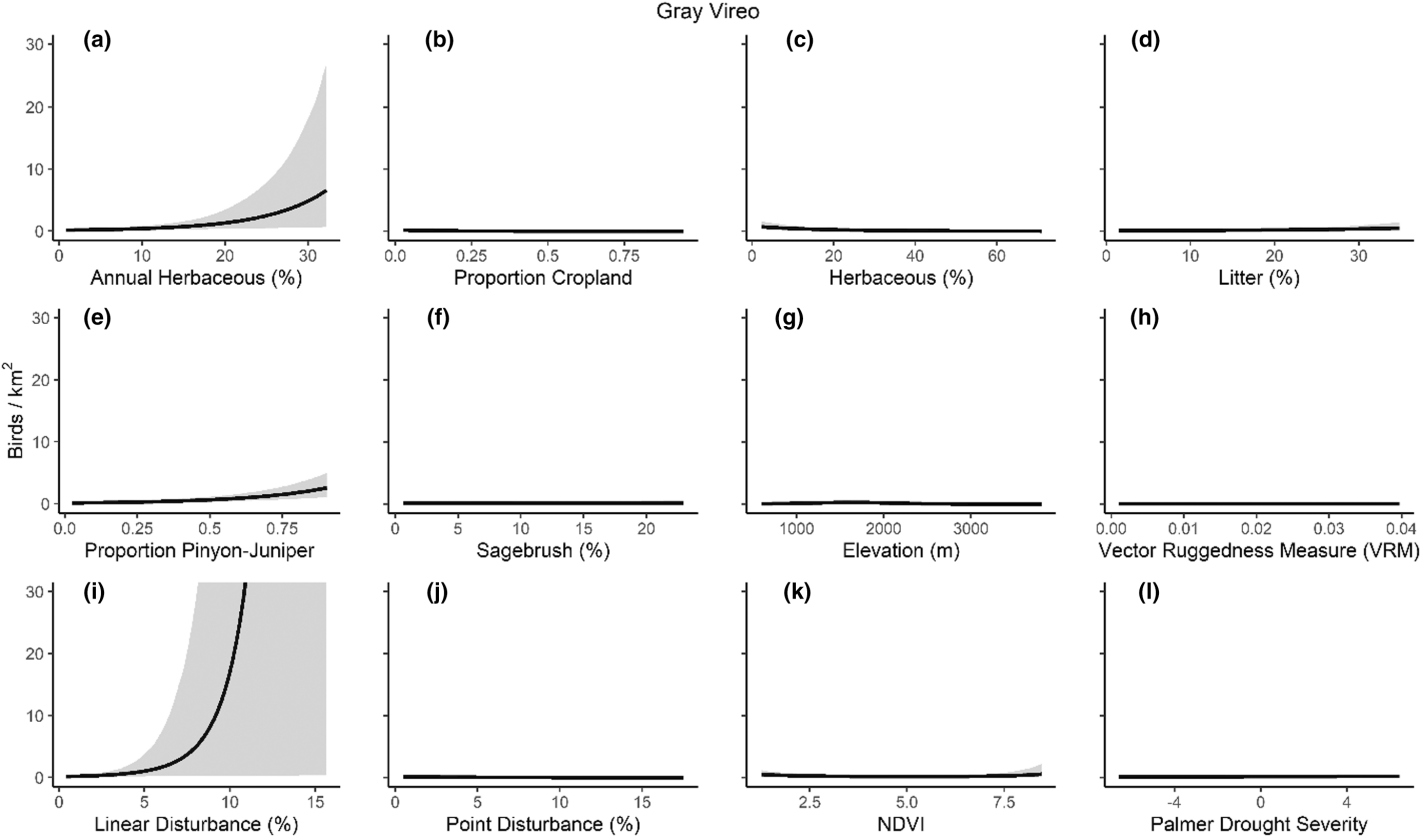
Gray Vireo mean density (birds/km^2^; line) and 95% credible intervals (ribbon) predicted as a function of covariate values within Bird Conservation Region 16. Relationships were modeled with a Bayesian hierarchical density‐habitat relationship model using point count data collected in the western United States; 2008–2020. Density was calculated by varying each covariate of interest while inputting mean values of all other covariates. Covariate values in panels a, c, d, and f were summarized using RCMAP data (Rigge et al., 2021). Cropland values (panel b) were derived from a binary raster layer developed using reclassified National Cropscape data (United States Department of Agriculture 2008–2020). Pinyon‐juniper cover values (panel e) were derived from binary rasters layer developed by reclassifying LANDFIRE existing vegetation types (LANDFIRE, 2008, 2010, 2012, 2014, 2016). Elevation values (panel g) were extracted at sample grid centroids from a national elevation data set (United States Department of Agriculture (USDA) Natural Resources Conservation Service, 2007). Vector Ruggedness Measures (panel h) were summarized from a product developed by O'Donnell et al. (2019). Linear and Point Disturbance values (panels i and j) were extracted from products developed by the Bureau of Land Management (Bureau of Land Management, Unpublished data). NDVI values (panel k) were derived by calculating means of maximum normalized difference vegetation index values for each pixel during the summer months (Didan, 2015). Palmer Drought Severity Index values (panel l; National Centers for Environmental Information, 2020) were extracted at the grid centroid.

**FIGURE 22:**
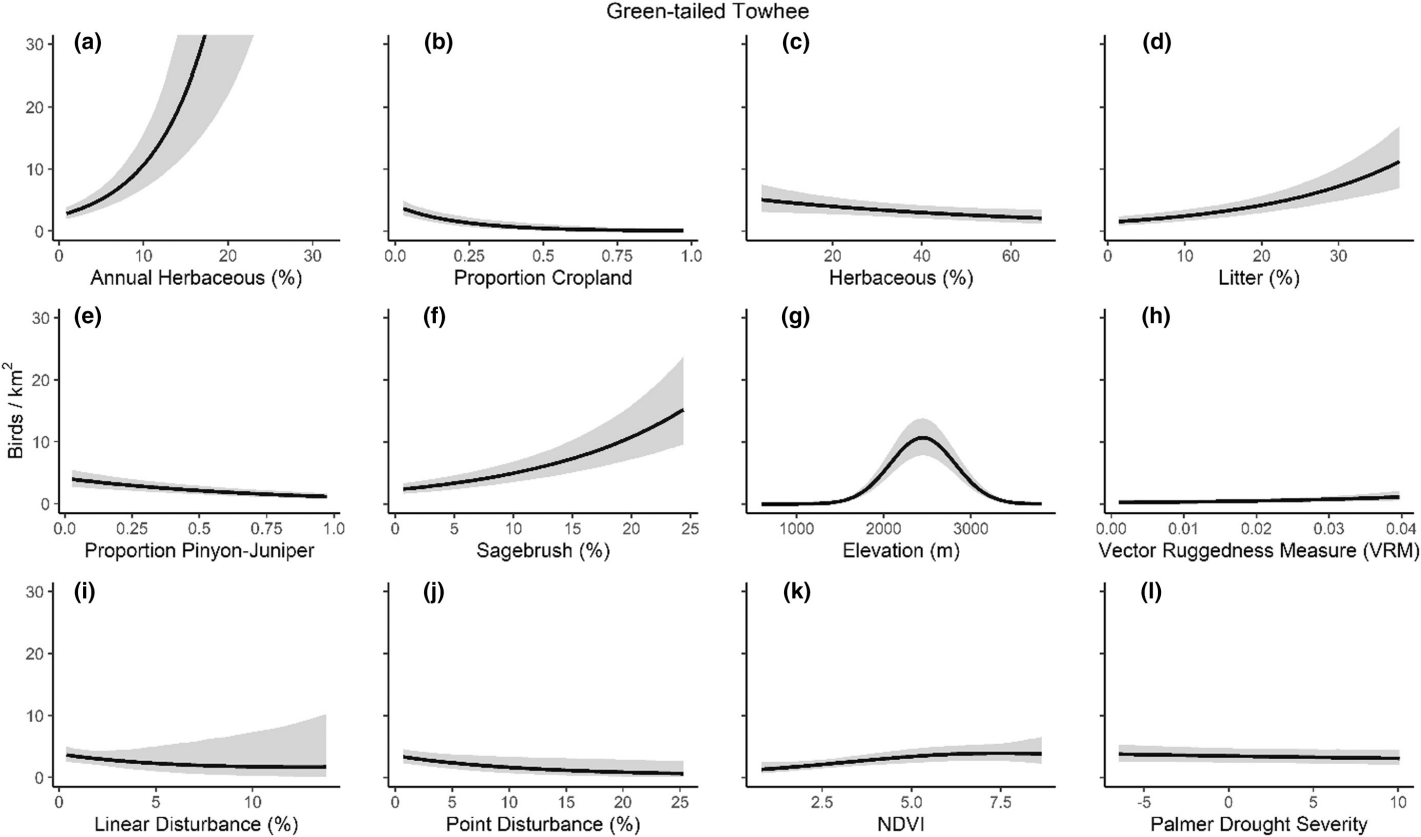
Green‐tailed Towhee mean density (birds/km^2^; line) and 95% credible intervals (ribbon) predicted as a function of covariate values within Bird Conservation Region 16. Relationships were modeled with a Bayesian hierarchical density‐habitat relationship model using point count data collected in the western United States; 2008–2020. Density was calculated by varying each covariate of interest while inputting mean values of all other covariates. Covariate values in panels a, c, d, and f were summarized using RCMAP data (Rigge et al., 2021). Cropland values (panel b) were derived from a binary raster layer developed using reclassified National Cropscape data (United States Department of Agriculture 2008–2020). Pinyon‐juniper cover values (panel e) were derived from binary rasters layer developed by reclassifying LANDFIRE existing vegetation types (LANDFIRE, 2008, 2010, 2012, 2014, 2016). Elevation values (panel g) were extracted at sample grid centroids from a national elevation data set (United States Department of Agriculture (USDA) Natural Resources Conservation Service, 2007). Vector Ruggedness Measures (panel h) were summarized from a product developed by O'Donnell et al. (2019). Linear and Point Disturbance values (panels i and j) were extracted from products developed by the Bureau of Land Management (Bureau of Land Management, Unpublished data). NDVI values (panel k) were derived by calculating means of maximum normalized difference vegetation index values for each pixel during the summer months (Didan, 2015). Palmer Drought Severity Index values (panel l; National Centers for Environmental Information, 2020) were extracted at the grid centroid.

**FIGURE 23:**
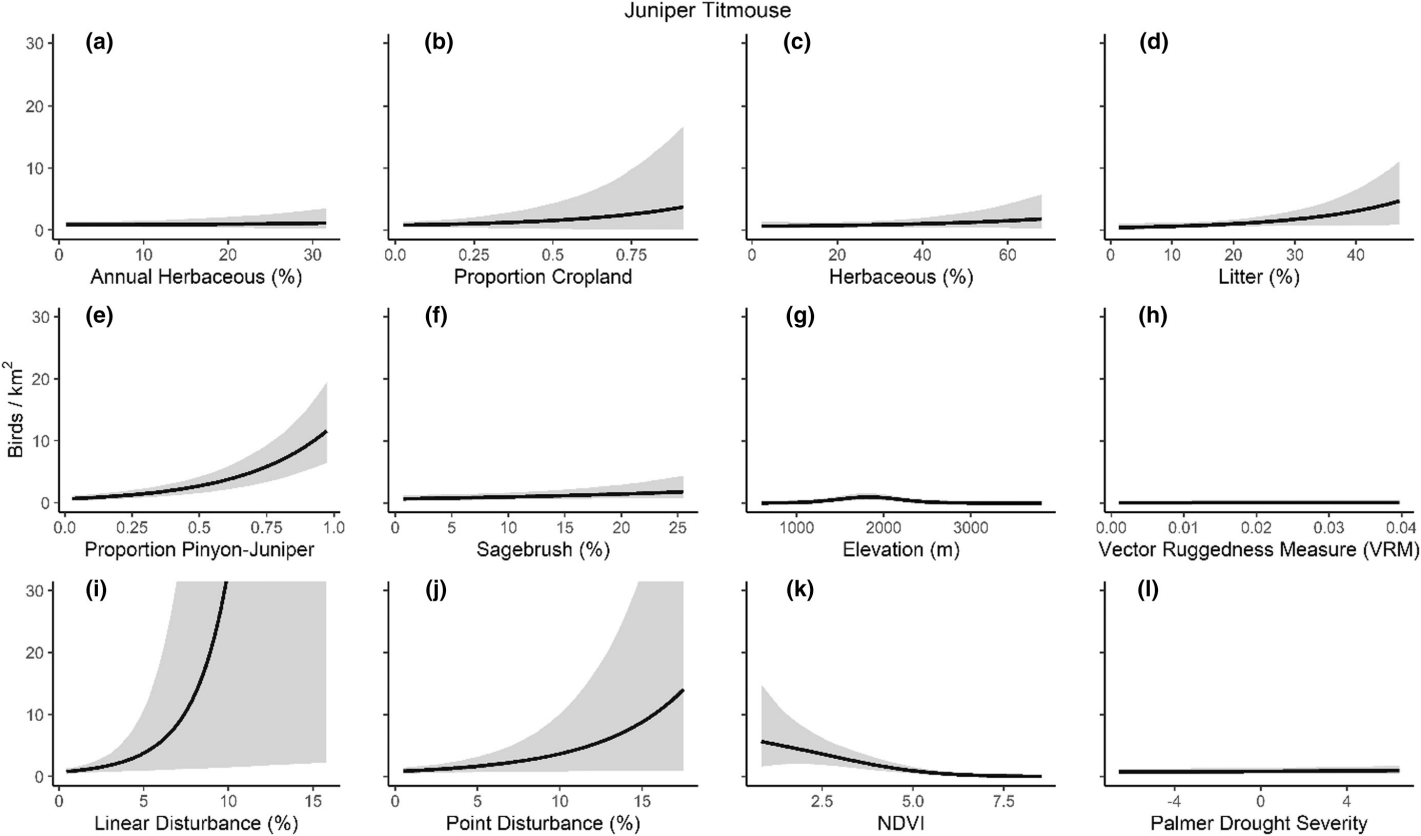
Juniper Titmouse mean density (birds/km^2^; line) and 95% credible intervals (ribbon) predicted as a function of covariate values within Bird Conservation Region 16. Relationships were modeled with a Bayesian hierarchical density‐habitat relationship model using point count data collected in the western United States; 2008–2020. Density was calculated by varying each covariate of interest while inputting mean values of all other covariates. Covariate values in panels a, c, d, and f were summarized using RCMAP data (Rigge et al., 2021). Cropland values (panel b) were derived from a binary raster layer developed using reclassified National Cropscape data (United States Department of Agriculture 2008–2020). Pinyon‐juniper cover values (panel e) were derived from binary rasters layer developed by reclassifying LANDFIRE existing vegetation types (LANDFIRE, 2008, 2010, 2012, 2014, 2016). Elevation values (panel g) were extracted at sample grid centroids from a national elevation data set (United States Department of Agriculture (USDA) Natural Resources Conservation Service, 2007). Vector Ruggedness Measures (panel h) were summarized from a product developed by O'Donnell et al. (2019). Linear and Point Disturbance values (panels i and j) were extracted from products developed by the Bureau of Land Management (Bureau of Land Management, Unpublished data). NDVI values (panel k) were derived by calculating means of maximum normalized difference vegetation index values for each pixel during the summer months (Didan, 2015). Palmer Drought Severity Index values (panel l; National Centers for Environmental Information, 2020) were extracted at the grid centroid.

**FIGURE 24:**
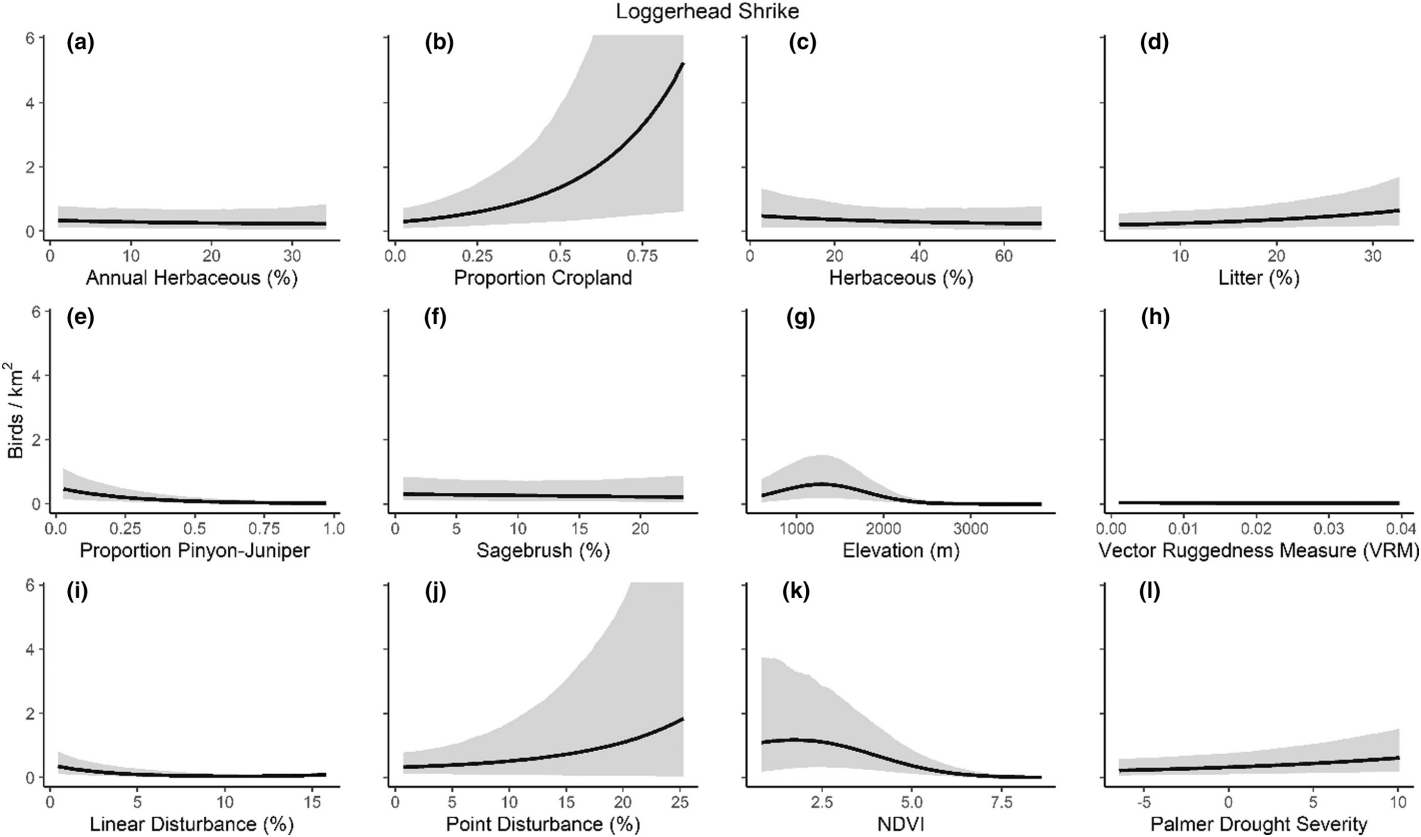
Loggerhead Shrike mean density (birds/km^2^; line) and 95% credible intervals (ribbon) predicted as a function of covariate values within Bird Conservation Region 16. Relationships were modeled with a Bayesian hierarchical density‐habitat relationship model using point count data collected in the western United States; 2008–2020. Density was calculated by varying each covariate of interest while inputting mean values of all other covariates. Covariate values in panels a, c, d, and f were summarized using RCMAP data (Rigge et al., 2021). Cropland values (panel b) were derived from a binary raster layer developed using reclassified National Cropscape data (United States Department of Agriculture 2008–2020). Pinyon‐juniper cover values (panel e) were derived from binary rasters layer developed by reclassifying LANDFIRE existing vegetation types (LANDFIRE, 2008, 2010, 2012, 2014, 2016). Elevation values (panel g) were extracted at sample grid centroids from a national elevation data set (United States Department of Agriculture (USDA) Natural Resources Conservation Service, 2007). Vector Ruggedness Measures (panel h) were summarized from a product developed by O'Donnell et al. (2019). Linear and Point Disturbance values (panels i and j) were extracted from products developed by the Bureau of Land Management (Bureau of Land Management, Unpublished data). NDVI values (panel k) were derived by calculating means of maximum normalized difference vegetation index values for each pixel during the summer months (Didan, 2015). Palmer Drought Severity Index values (panel l; National Centers for Environmental Information, 2020) were extracted at the grid centroid.

**FIGURE 25:**
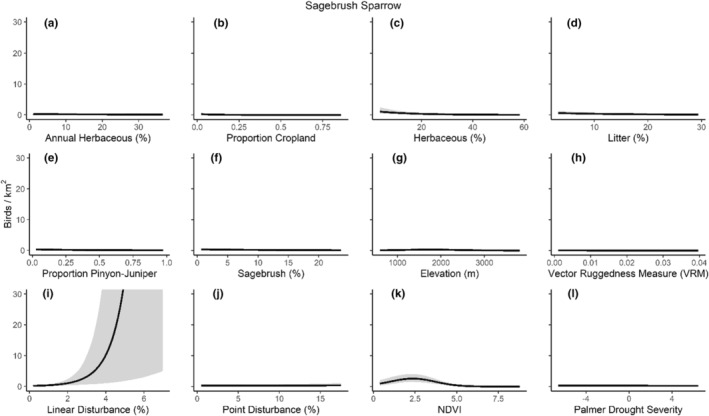
Sagebrush Sparrow mean density (birds/km^2^; line) and 95% credible intervals (ribbon) predicted as a function of covariate values within Bird Conservation Region 10. Relationships were modeled with a Bayesian hierarchical density‐habitat relationship model using point count data collected in the western United States; 2008–2020. Density was calculated by varying each covariate of interest while inputting mean values of all other covariates. Covariate values in panels a, c, d, and f were summarized using RCMAP data (Rigge et al., 2021). Cropland values (panel b) were derived from a binary raster layer developed using reclassified National Cropscape data (United States Department of Agriculture 2008–2020). Pinyon‐juniper cover values (panel e) were derived from binary rasters layer developed by reclassifying LANDFIRE existing vegetation types (LANDFIRE, 2008, 2010, 2012, 2014, 2016). Elevation values (panel g) were extracted at sample grid centroids from a national elevation data set (United States Department of Agriculture (USDA) Natural Resources Conservation Service, 2007). Vector Ruggedness Measures (panel h) were summarized from a product developed by O'Donnell et al. (2019). Linear and Point Disturbance values (panels i and j) were extracted from products developed by the Bureau of Land Management (Bureau of Land Management, Unpublished data). NDVI values (panel k) were derived by calculating means of maximum normalized difference vegetation index values for each pixel during the summer months (Didan, 2015). Palmer Drought Severity Index values (panel l; National Centers for Environmental Information, 2020) were extracted at the grid centroid.

**FIGURE 26:**
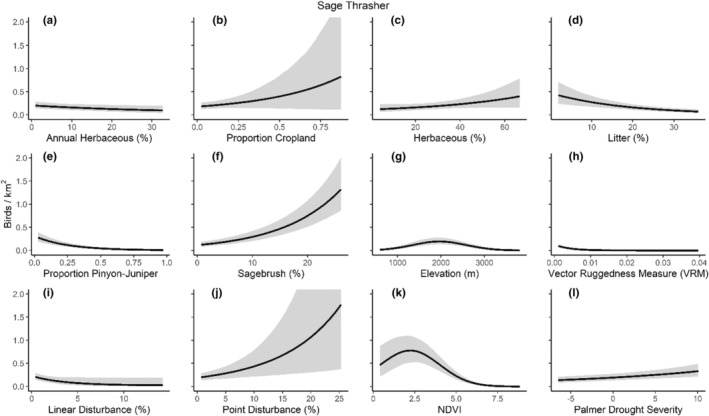
Sage Thrasher mean density (birds/km^2^; line) and 95% credible intervals (ribbon) predicted as a function of covariate values within Bird Conservation Region 10. Relationships were modeled with a Bayesian hierarchical density‐habitat relationship model using point count data collected in the western United States; 2008–2020. Density was calculated by varying each covariate of interest while inputting mean values of all other covariates. Covariate values in panels a, c, d, and f were summarized using RCMAP data (Rigge et al., 2021). Cropland values (panel b) were derived from a binary raster layer developed using reclassified National Cropscape data (United States Department of Agriculture 2008–2020). Pinyon‐juniper cover values (panel e) were derived from binary rasters layer developed by reclassifying LANDFIRE existing vegetation types (LANDFIRE, 2008, 2010, 2012, 2014, 2016). Elevation values (panel g) were extracted at sample grid centroids from a national elevation data set (United States Department of Agriculture (USDA) Natural Resources Conservation Service, 2007). Vector Ruggedness Measures (panel h) were summarized from a product developed by O'Donnell et al. (2019). Linear and Point Disturbance values (panels i and j) were extracted from products developed by the Bureau of Land Management (Bureau of Land Management, Unpublished data). NDVI values (panel k) were derived by calculating means of maximum normalized difference vegetation index values for each pixel during the summer months (Didan, 2015). Palmer Drought Severity Index values (panel l; National Centers for Environmental Information, 2020) were extracted at the grid centroid.

**FIGURE 27:**
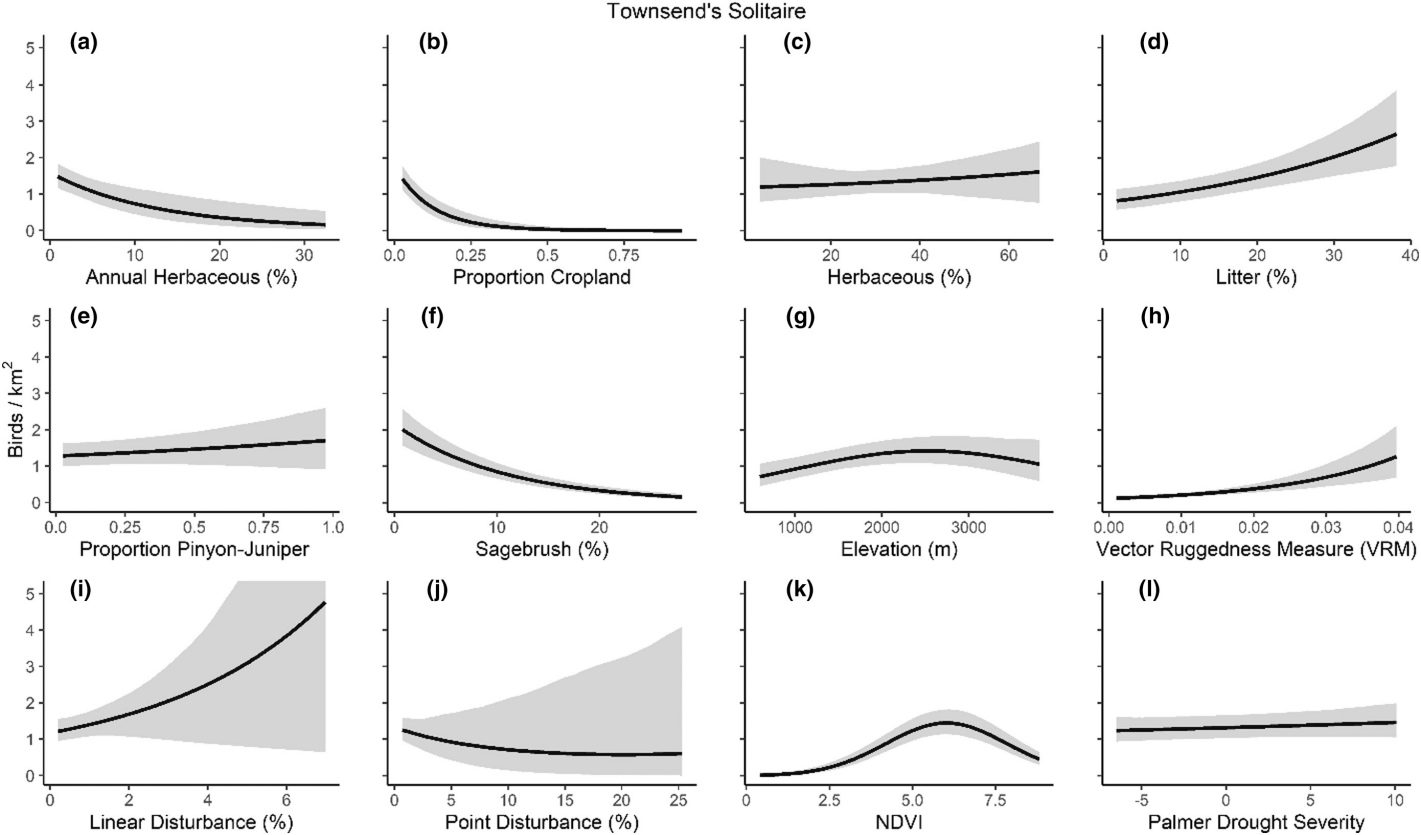
Townsend's Solitaire mean density (birds/km^2^; line) and 95% credible intervals (ribbon) predicted as a function of covariate values within Bird Conservation Region 10. Relationships were modeled with a Bayesian hierarchical density‐habitat relationship model using point count data collected in the western United States; 2008–2020. Density was calculated by varying each covariate of interest while inputting mean values of all other covariates. Covariate values in panels a, c, d, and f were summarized using RCMAP data (Rigge et al., 2021). Cropland values (panel b) were derived from a binary raster layer developed using reclassified National Cropscape data (United States Department of Agriculture 2008–2020). Pinyon‐juniper cover values (panel e) were derived from binary rasters layer developed by reclassifying LANDFIRE existing vegetation types (LANDFIRE, 2008, 2010, 2012, 2014, 2016). Elevation values (panel g) were extracted at sample grid centroids from a national elevation data set (United States Department of Agriculture (USDA) Natural Resources Conservation Service, 2007). Vector Ruggedness Measures (panel h) were summarized from a product developed by O'Donnell et al. (2019). Linear and Point Disturbance values (panels i and j) were extracted from products developed by the Bureau of Land Management (Bureau of Land Management, Unpublished data). NDVI values (panel k) were derived by calculating means of maximum normalized difference vegetation index values for each pixel during the summer months (Didan, 2015). Palmer Drought Severity Index values (panel l; National Centers for Environmental Information, 2020) were extracted at the grid centroid.

Anthropogenic disturbance was associated with densities of 8 of the 11 species for which we modeled abundance (Figures 14, 15, 16; Table 6). We found support for relationships between songbird densities and linear anthropogenic disturbance for seven species. One sagebrush‐associated (Sagebrush Sparrow) and two pinyon‐juniper‐associated species (Juniper Titmouse and Gray Vireo) demonstrated strong positive associations with increasing linear disturbance; however, there was substantial uncertainty regarding these relationships. In contrast, we found densities of one pinyon‐juniper‐associated species (Black‐throated Gray Warbler), two sagebrush‐associated species (Brewer's Sparrow and Sage Thrasher), and one generalist species (Loggerhead Shrike) were moderately and negatively associated with linear disturbance features (Figures 14, 15, 16; Table 6). One sagebrush‐associated species (Sage Thrasher) and one pinyon‐juniper‐associated species (Juniper Titmouse) demonstrated strong positive density associations with increasing amounts of point disturbance; however, as with the linear disturbance relationships, there was substantial uncertainty associated with these relationships (Figures 14, 15, 16; Table 6). We estimated weak to moderate negative relationships between two pinyon‐juniper‐associated species (Black‐throated Gray Warbler and Gray Vireo) and one sagebrush‐associated species (Green‐tailed Towhee) densities and point disturbance (Figures 14, 15, 16; Table 6). We provide figures of the density‐covariate relationships for each species between the 2.5% and 97.5% quantiles associated with the covariate data used to inform the model for each species (Figures 17, 18, 19, 20, 21, 22, 23, 24, 25, 26, 27).

**FIGURE 28:**
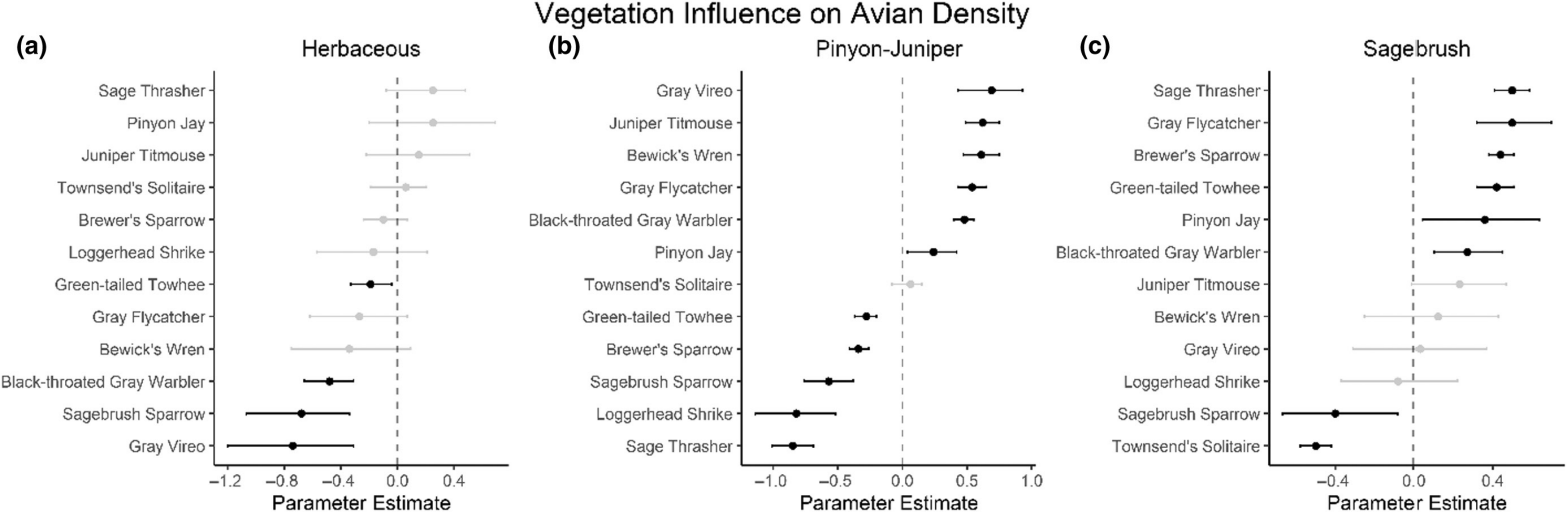
Point estimates (dots) and associated 95% credible intervals (whiskers) for hierarchical population model parameters associated with the influence of herbaceous (a), pinyon‐juniper (b), and sagebrush (c) cover on songbird densities in the western United States of America; 2008–2020. Density‐habitat relationships for Pinyon Jay are from Van Lanen et al. (2023a). Black whiskers indicate the credible interval does not overlap zero.

**FIGURE 29:**
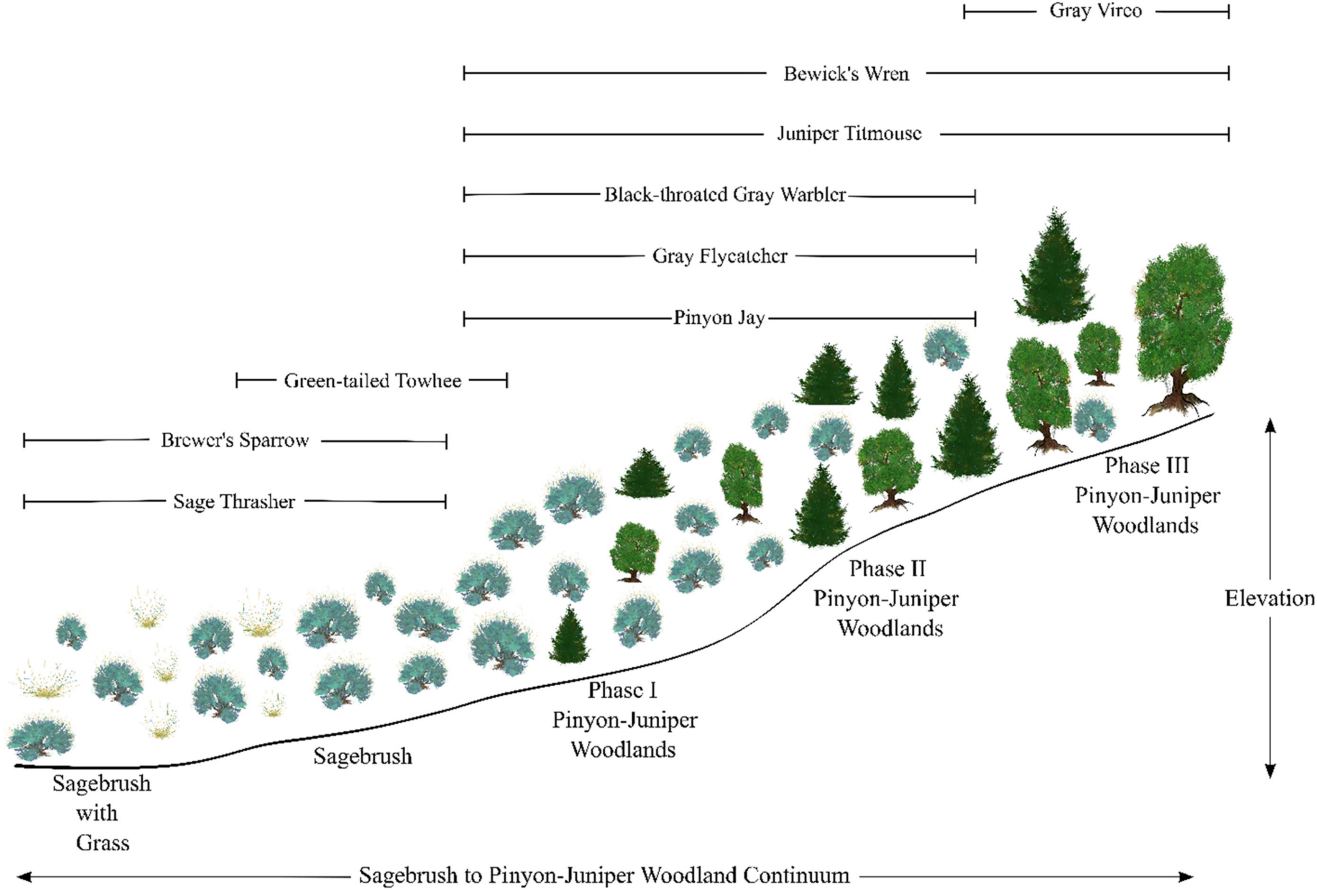
Communities predicted to support highest songbird densities for nine species positively associated with sagebrush and/or pinyon‐juniper cover within the sagebrush and pinyon‐juniper woodland continuum. Results shown are based upon bird density and herbaceous, sagebrush, and pinyon‐juniper relationships developed using a hierarchical Bayesian density‐habitat model. Density‐habitat relationships for Pinyon Jay are from Van Lanen et al. (2023a). Modeled relationships were informed via point count data collected across the western United States of America; 2008–2020. The elevation pattern shown is a general representation of vegetation community distribution within the study area and does not reflect modeled songbird density‐elevation relationships explicitly. Three species included in this study (Loggerhead Shrike, Sagebrush Sparrow, and Townsend's Solitaire) were not positively associated with either sagebrush or pinyon‐juniper cover and are therefore not represented in this figure.

Given the positive association with sagebrush cover and negative association with the proportion of pinyon‐juniper (Figure 28), we expect Brewer's Sparrow, Green‐tailed Towhee, and Sage Thrasher will occur at highest densities within sagebrush ecosystems along sagebrush and pinyon‐juniper ecotones (Figure 29). Due to the positive association with sagebrush cover and negative relationship with herbaceous cover, we expect Green‐tailed Towhee will occur at maximum densities within sagebrush ecosystems lacking significant grass cover. Our findings suggest Black‐throated Gray Warbler and Gray Flycatcher will occur at highest densities in Phase I and Phase II pinyon‐juniper woodlands (Miller et al., 2019), given their positive associations with both sagebrush and proportion of pinyon‐juniper. Similarly, we expect Bewick's Wren and Juniper Titmouse will occur at highest densities within all three pinyon‐juniper woodland phases due to their positive association with the proportion of pinyon‐juniper and limited associations with sagebrush and herbaceous cover. The positive density‐habitat association with proportion of pinyon‐juniper and negative association with herbaceous cover indicate Gray Vireo may occur at highest densities within Phase III pinyon‐juniper woodlands (Figures 28 and 29). Finally, Loggerhead Shrike, Sagebrush Sparrow, and Townsend's Solitaire were not positively associated with sagebrush or the proportion of pinyon‐juniper (Figure 28). As a result, we do not expect these species to occur at high densities within any successional phases of sagebrush and pinyon‐juniper ecotones and therefore excluded them from our conceptual diagram (Figure 29).

We found support for 15 species‐covariate relationships relevant to a changing climate. Normalized difference vegetation index (NDVI) values were moderately to strongly associated with densities for 10 species (Figures 14, 15, 16; Table 6). One pinyon‐juniper associate (Gray Flycatcher) was strongly and negatively associated with NDVI, while one sagebrush associate (Sage Thrasher) and one generalist (Townsend's Solitaire) were strongly associated with intermediate NDVI values. Our NDVI index of vegetation potential was moderately associated with densities for eight species. One sagebrush associate (Green‐tailed Towhee) was moderately and positively associated while two pinyon‐juniper associates (Black‐throated Gray Warbler and Juniper Titmouse) and one generalist (Loggerhead Shrike) were moderately and negatively associated with NDVI values. One generalist (Bewick's Wren) and two sagebrush associates (Brewer's Sparrow and Sagebrush Sparrow) were moderately correlated with intermediate NDVI values (Table 6). Palmer Drought Severity Index (PDSI) values were modestly and positively associated with densities of one generalist (Loggerhead Shrike) and one sagebrush associate (Sage Thrasher) and weakly and positively associated with one sagebrush associate (Brewer's Sparrow) and two pinyon‐juniper associates (Gray Flycatcher and Gray Vireo) (Figures 14, 15, 16; Table 6).

### 3.4 Mapped predicted densities in 2020

**FIGURE 30:**
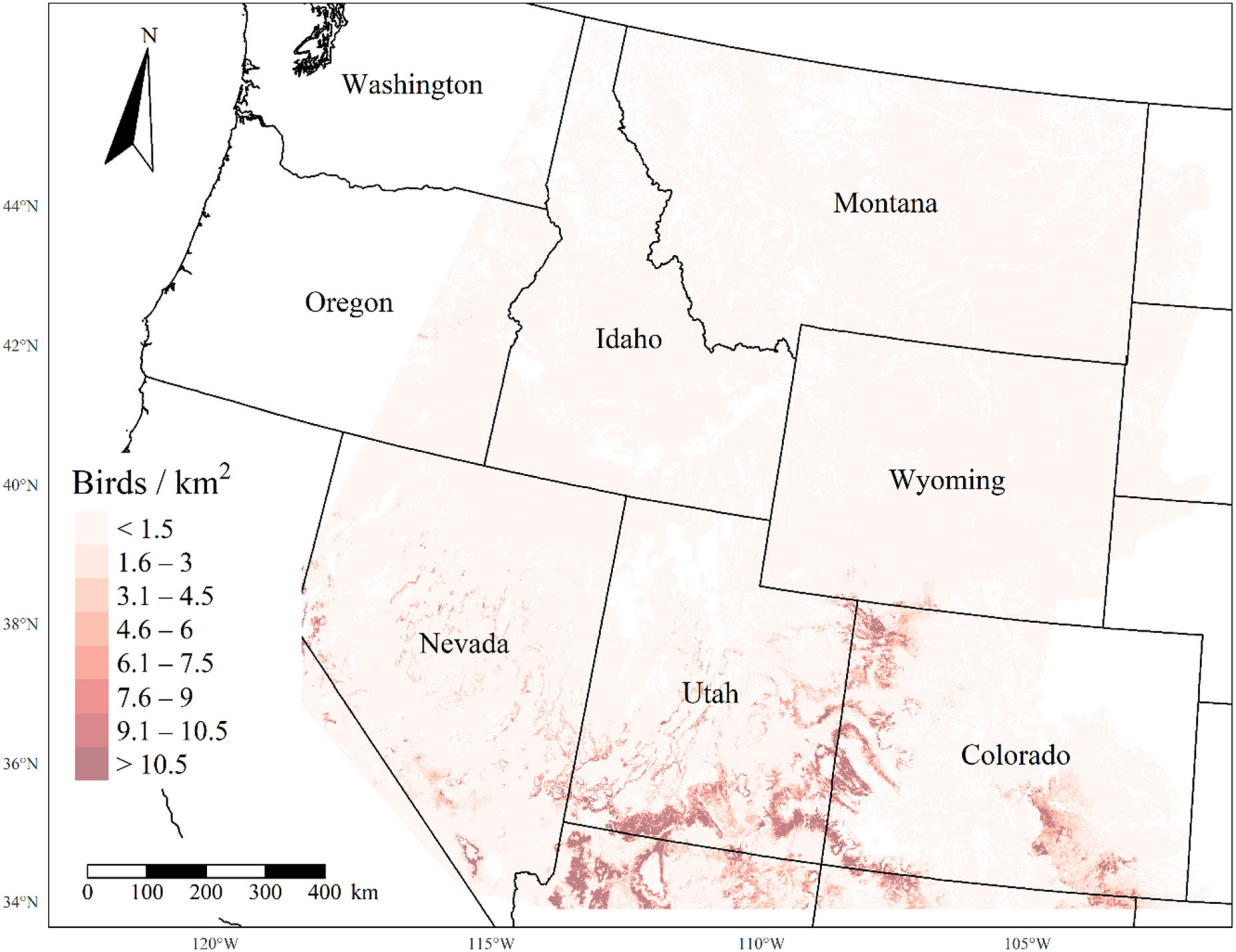
Predicted median density of Bewick's Wren (*Thryomanes bewickii*) in May–July 2020, based upon hierarchical Bayesian density‐habitat relationships, throughout the InterMountain West region of the USA. Density was not predicted for regions in white. Bases modified from National Weather Service, 1:2,000,000, 1980.

**FIGURE 31:**
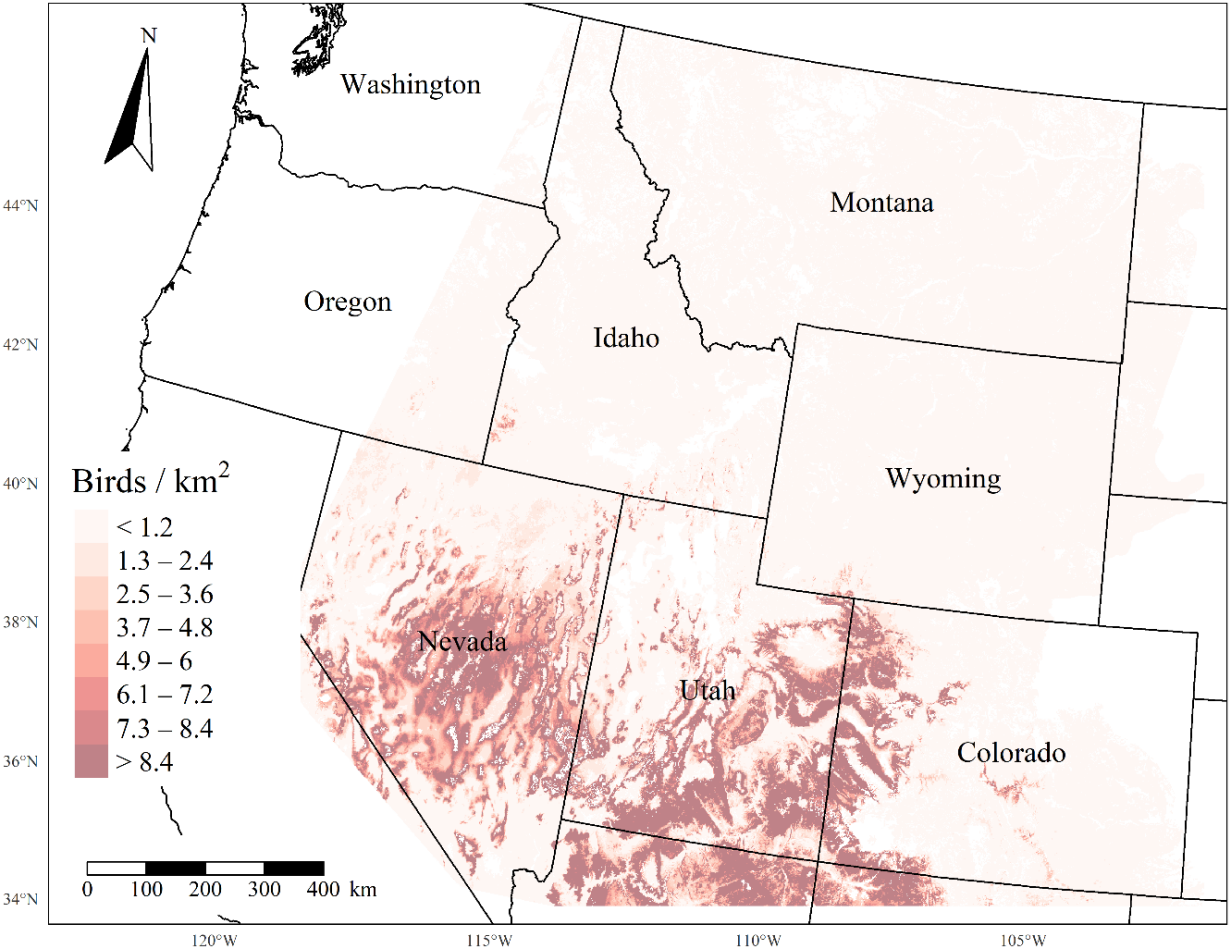
Predicted median density of Black‐throated Gray Warbler (*Setophaga nigrescens*) in May–July 2020, based upon hierarchical Bayesian density‐habitat relationships, throughout the InterMountain West region of the USA. Density was not predicted for regions in white. Bases modified from National Weather Service, 1:2,000,000, 1980.

**FIGURE 32:**
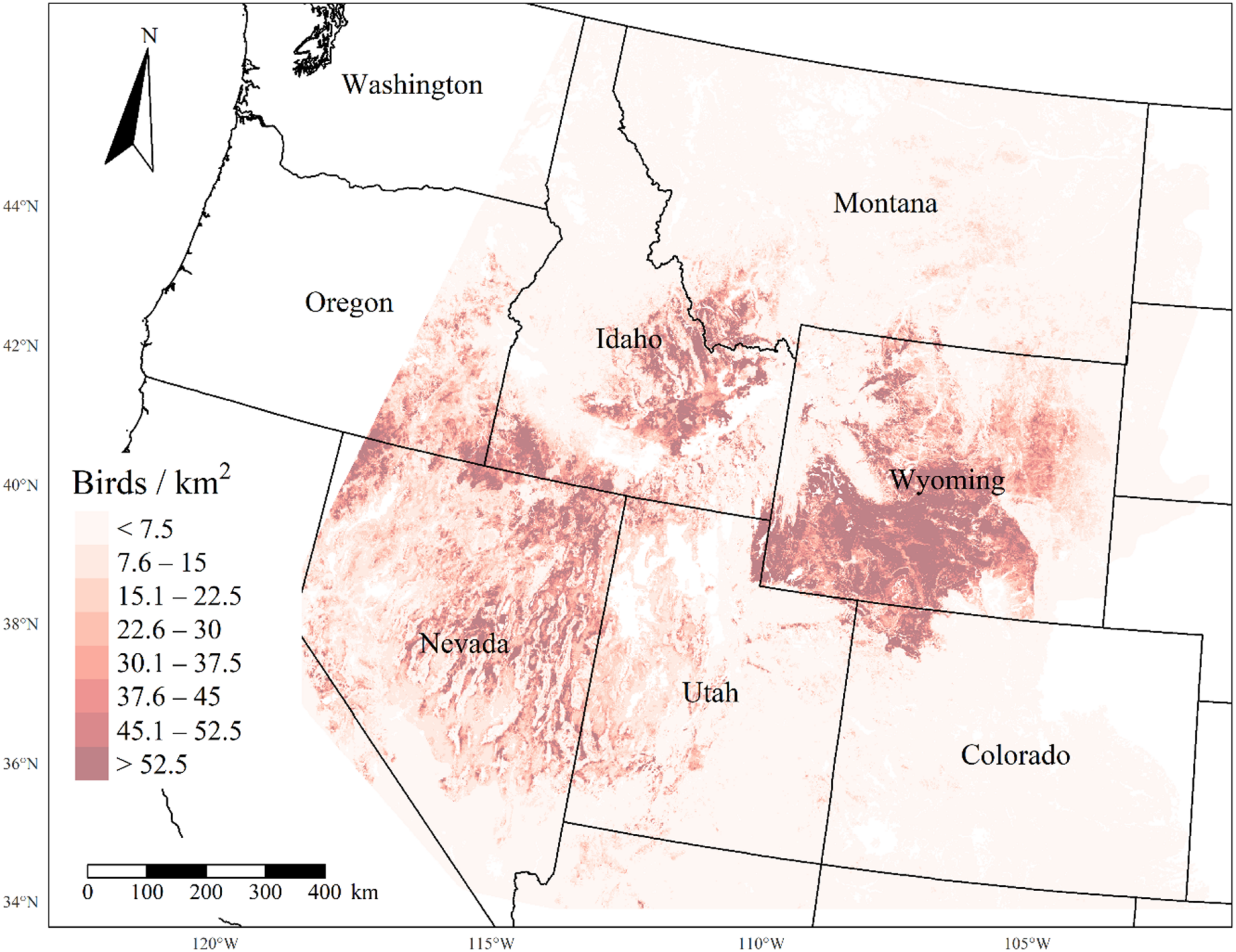
Predicted median density of Brewer's Sparrow (*Spizella breweri*) in May–July 2020, based upon hierarchical Bayesian density‐habitat relationships, throughout the InterMountain West region of the USA. Density was not predicted for regions in white. Bases modified from National Weather Service, 1:2,000,000, 1980.

**FIGURE 33:**
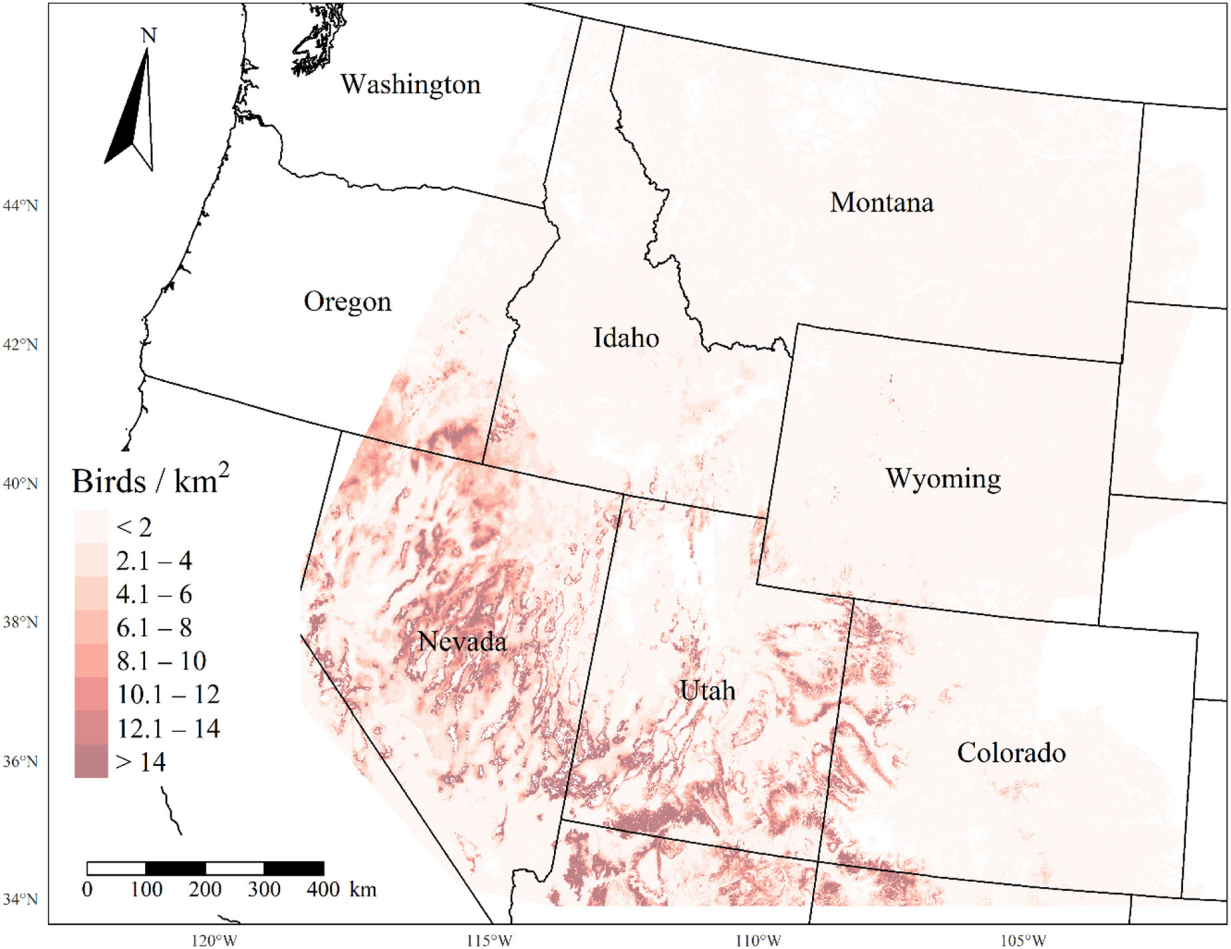
Predicted median density of Gray Flycatcher (*Empidonax wrightii*) in May–July 2020, based upon hierarchical Bayesian density‐habitat relationships, throughout the InterMountain West region of the USA. Density was not predicted for regions in white. Bases modified from National Weather Service, 1:2,000,000, 1980.

**FIGURE 34:**
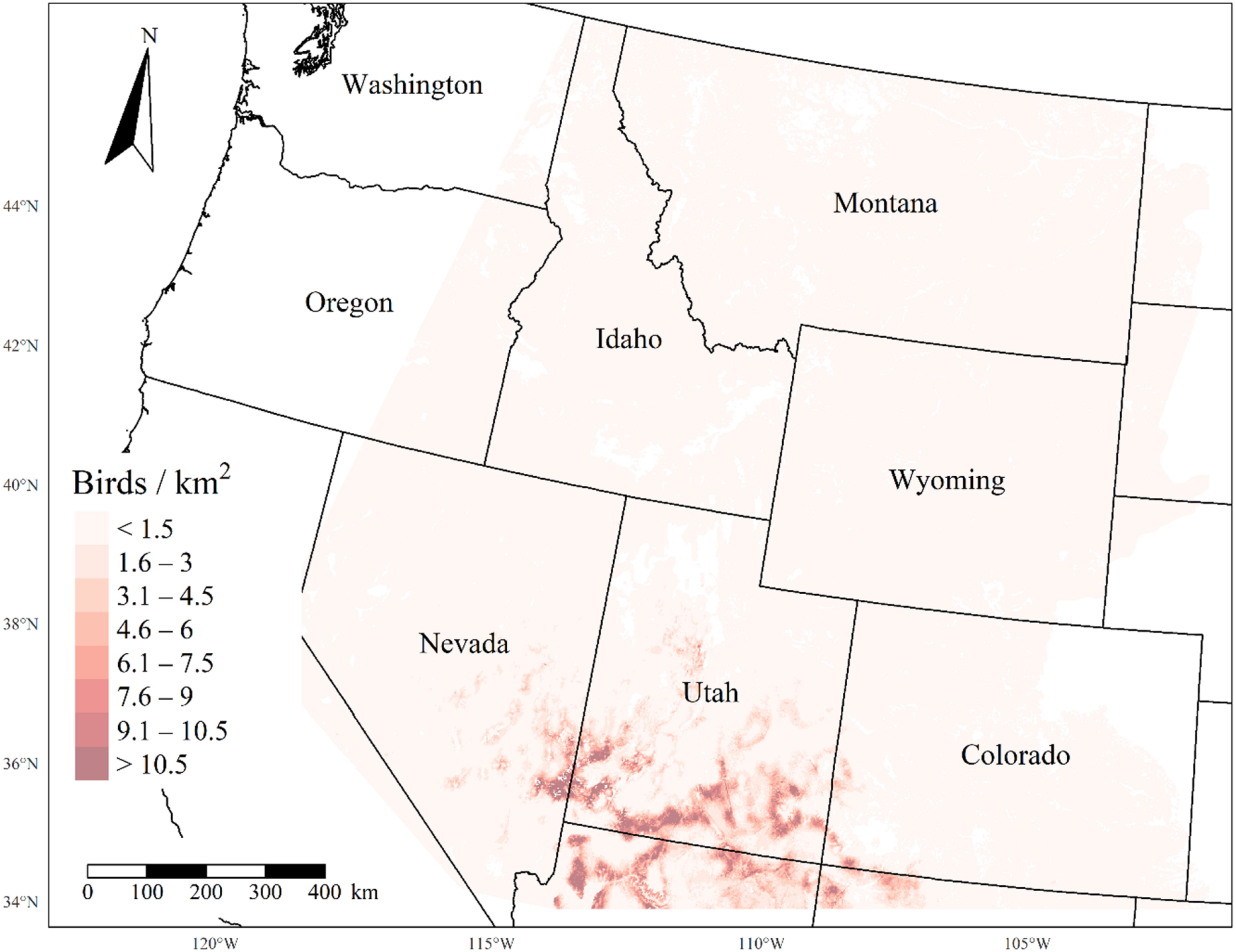
Predicted median density of Gray Vireo (*Vireo vicinior*) in May–July 2020, based upon hierarchical Bayesian density‐habitat relationships, throughout the InterMountain West region of the USA. Density was not predicted for regions in white. Bases modified from National Weather Service, 1:2,000,000, 1980.

**FIGURE 35:**
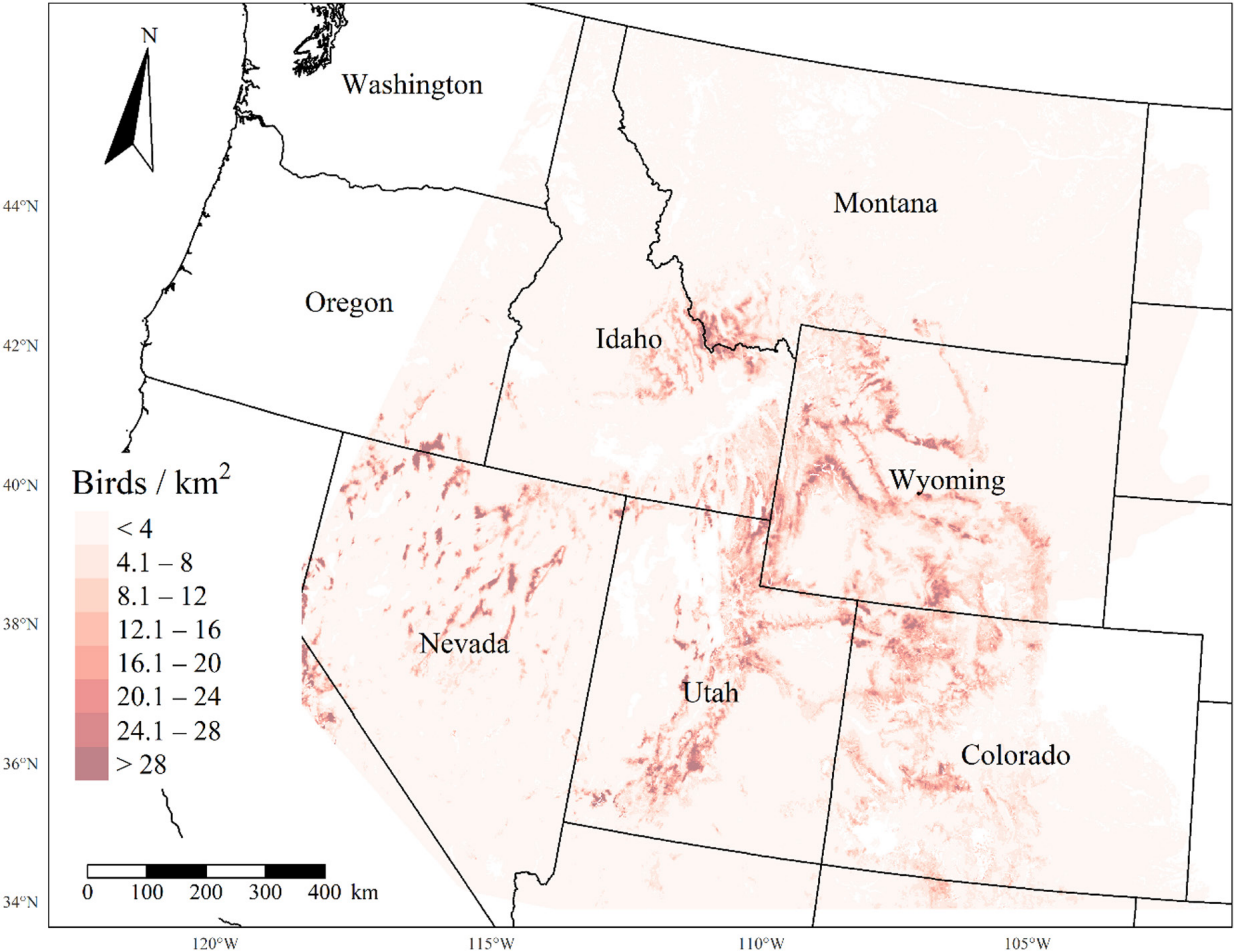
Predicted median density of Green‐tailed Towhee (*Pipilo chlorurus*) in May–July 2020, based upon hierarchical Bayesian density‐habitat relationships, throughout the InterMountain West region of the USA. Density was not predicted for regions in white. Bases modified from National Weather Service, 1:2,000,000, 1980.

**FIGURE 36:**
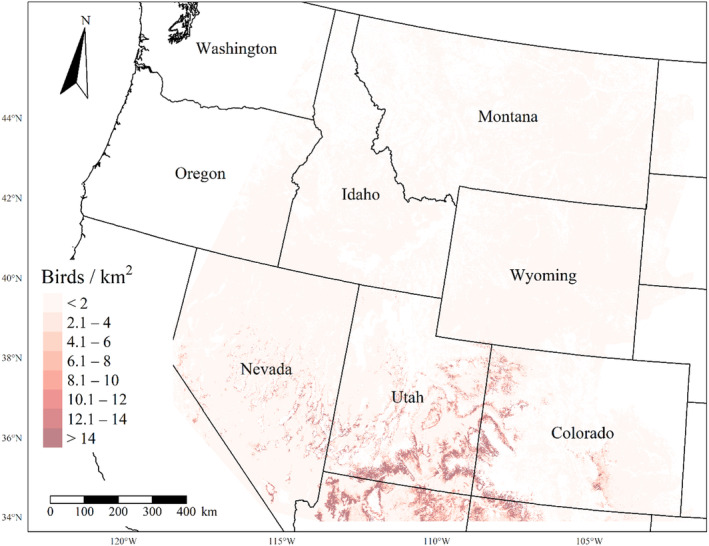
Predicted median density of Juniper Titmouse (*Baeolophus ridgwayi*) in May–July 2020, based upon hierarchical Bayesian density‐habitat relationships, throughout the InterMountain West region of the USA. Density was not predicted for regions in white. Bases modified from National Weather Service, 1:2,000,000, 1980.

**FIGURE 37:**
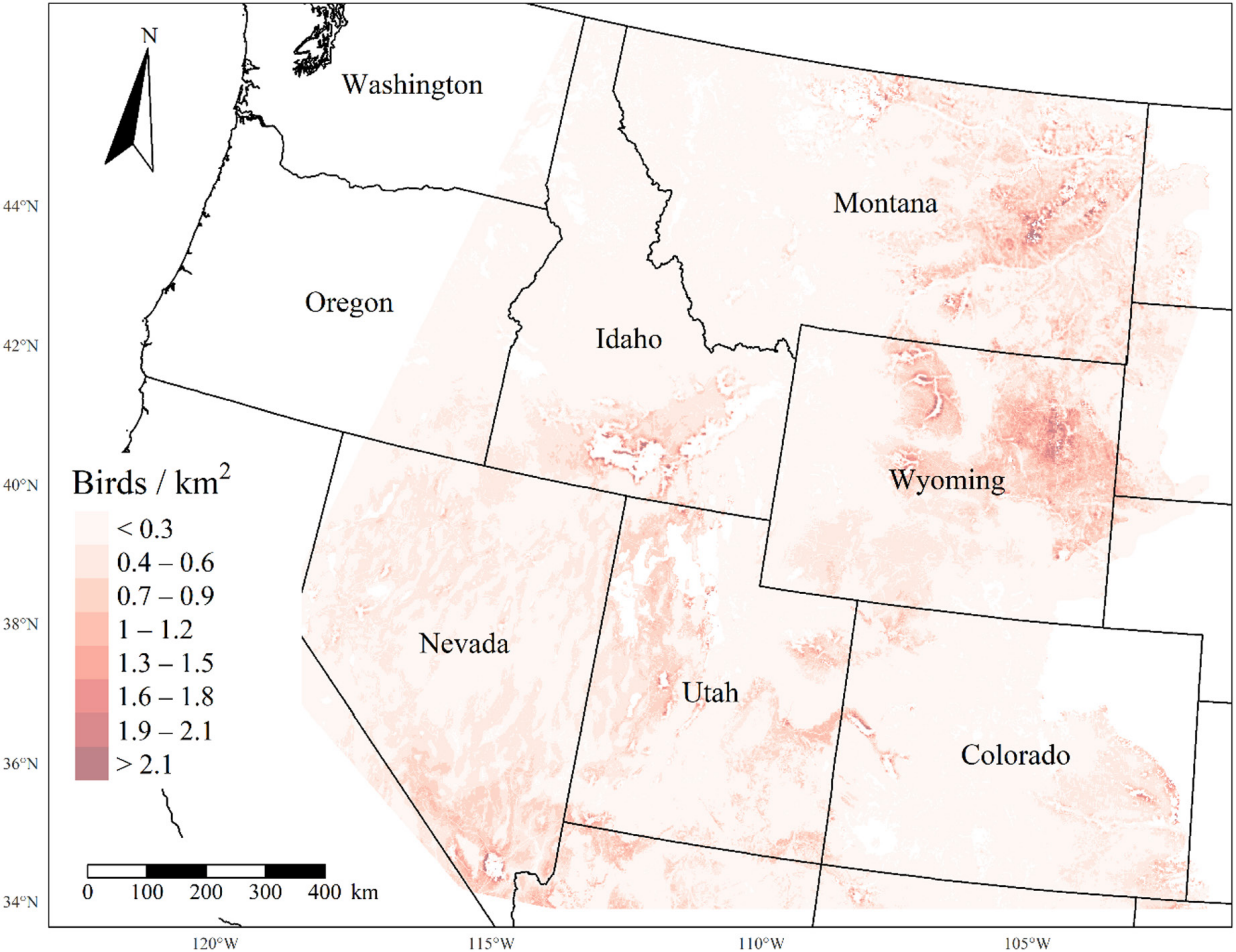
Predicted median density of Loggerhead Shrike (*Lanius ludovicianus*) in May–July 2020, based upon hierarchical Bayesian density‐habitat relationships, throughout the InterMountain West region of the USA. Density was not predicted for regions in white. Bases modified from National Weather Service, 1:2,000,000, 1980.

**FIGURE 38:**
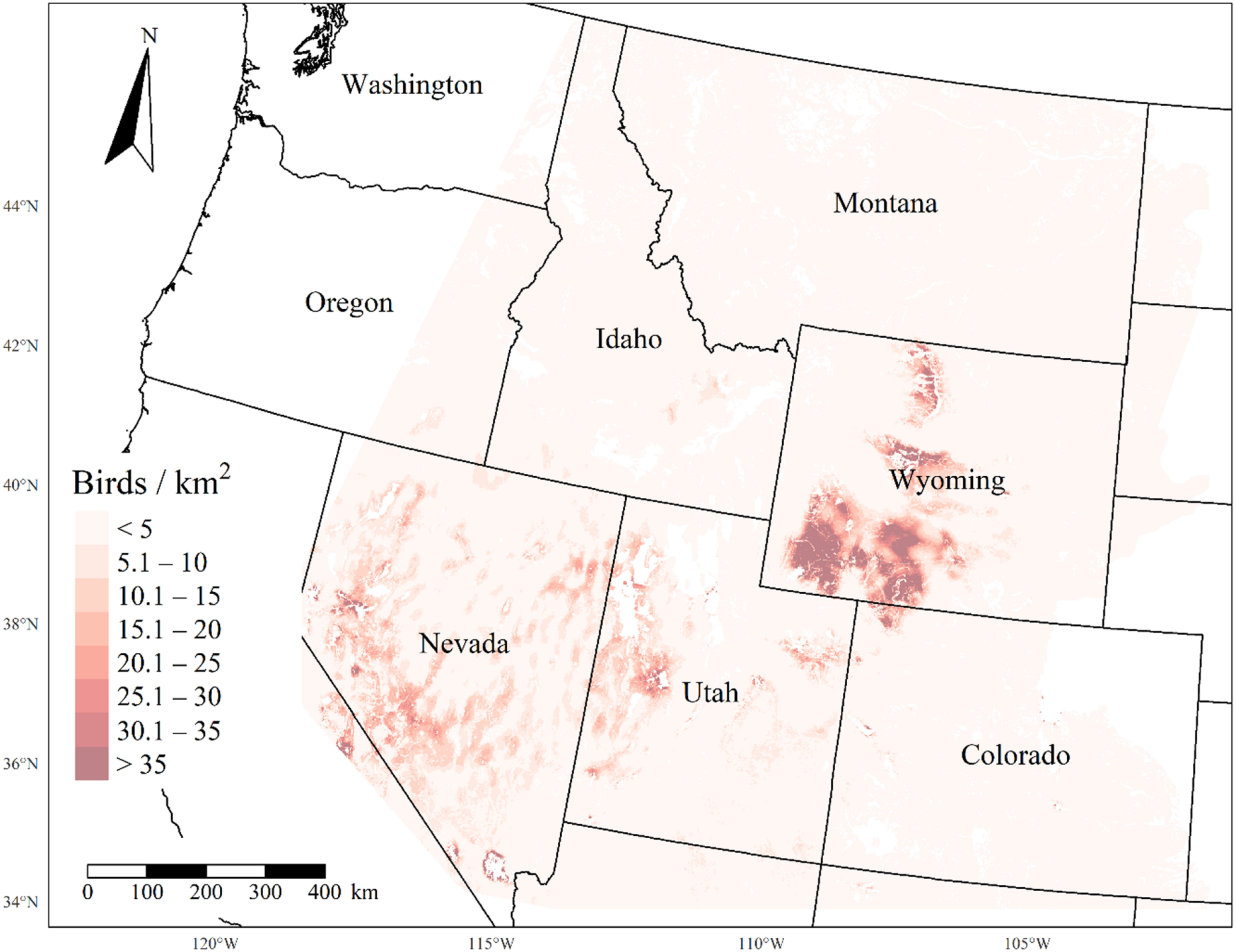
Predicted median density of Sagebrush Sparrow (*Artemisiospiza nevadensis*) in May–July 2020, based upon hierarchical Bayesian density‐habitat relationships, throughout the InterMountain West region of the USA. Density was not predicted for regions in white. Bases modified from National Weather Service, 1:2,000,000, 1980.

**FIGURE 39:**
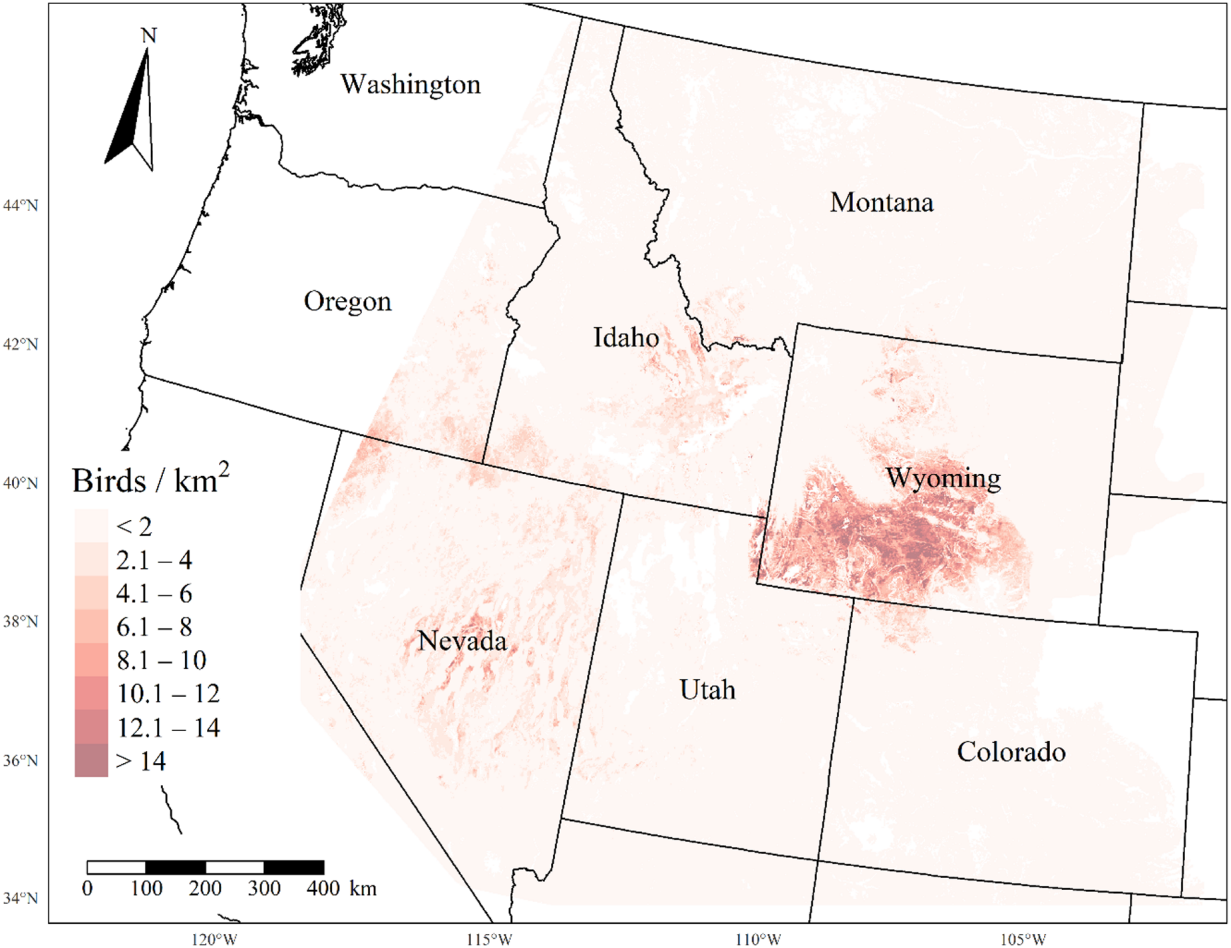
Predicted median density of Sage Thrasher (*Oreoscoptes montanus*) in May–July 2020, based upon hierarchical Bayesian density‐habitat relationships, throughout the InterMountain West region of the USA. Density was not predicted for regions in white. Bases modified from National Weather Service, 1:2,000,000, 1980.

**FIGURE 40:**
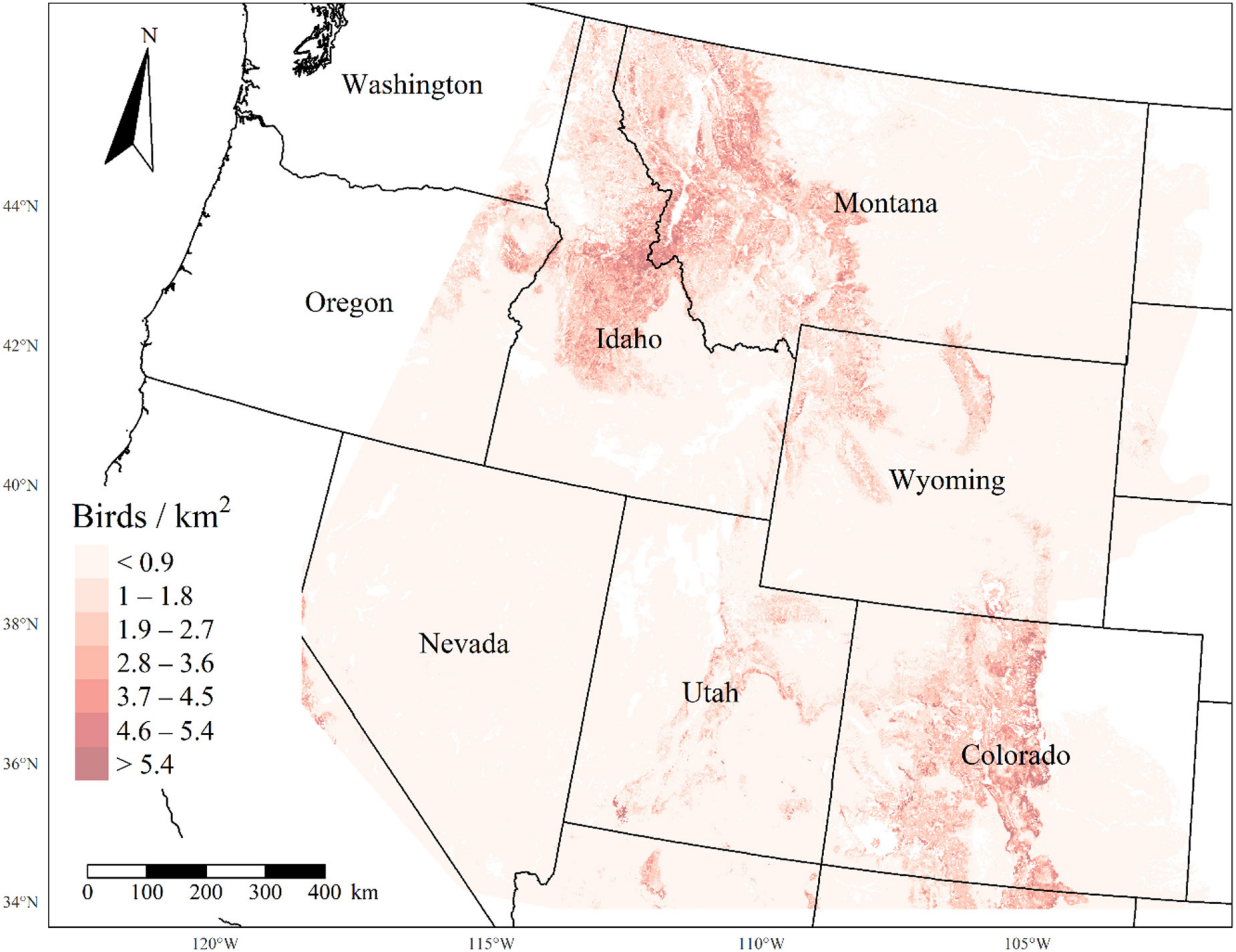
Predicted median density of Townsend's Solitaire (*Myadestes townsendi*) in May–July 2020, based upon hierarchical Bayesian density‐habitat relationships, throughout the InterMountain West region of the USA. Density was not predicted for regions in white. Bases modified from National Weather Service, 1:2,000,000, 1980.

We mapped median predicted densities throughout our study area for 11 species of interest, given 2020 conditions (Figures 30, 31, 32, 33, 34, 35, 36, 37, 38, 39, 40). We predicted highest Bewick's Wren densities south of Wyoming, particularly along the border between Utah and Colorado, in southwestern Utah, and in northern Arizona (Figure 30). We predicted greatest Black‐throated Gray Warbler densities in the southwestern portion of our study area, specifically in western Colorado, throughout much of Utah and Nevada, and in northern Arizona (Figure 31). We predicted high Brewer's Sparrow densities throughout much of Wyoming, along the northern border of Nevada, and in south‐central Idaho (Figure 32). Our model predicted highest Gray Flycatcher densities in southwestern Oregon, throughout much of Nevada, in the southeastern half of Utah, western Colorado, and in northern Arizona (Figure 33). Predicted Gray Vireo density was greatest in eastern Nevada, southern Utah, and northern Arizona (Figure 34). We predicted highest Green‐tailed Towhee densities along the edges of mountain ranges in southwestern Montana, western Wyoming and Colorado, central Utah, and northern Nevada (Figure 35). Predicted Juniper Titmouse densities were greatest in southern Utah, western Colorado, northern Arizona, and northwestern New Mexico (Figure 36). Our model predicted Loggerhead Shrike density was greatest in eastern Montana, northeastern Wyoming, southern Idaho, western Utah, and throughout Nevada (Figure 37). Our mapping of Sagebrush Sparrow density indicated density was greatest in west‐central Wyoming, western Utah, and dispersed throughout Nevada (Figure 38). We predicted highest Sage Thrasher density in central and southwestern Wyoming (Figure 39). Finally, we predict Townsend's Solitaire density to be highest along the Rocky Mountain range, in northern Idaho, western Montana, northwestern Wyoming, and western Colorado (Figure 40).

## 4 DISCUSSION

To our knowledge, our research represents the first effort to model and map density across much of the western United States for 11 songbird species occurring within sagebrush ecosystems, pinyon‐juniper woodlands, and their ecotones. Our modeling revealed that populations for most of our focal species were increasing or decreasing within at least one Bird Conservation Region during the timeframe of our study (Figure 13, Table 4), demonstrating the potential utility of continued monitoring and trend assessment to prioritize species for conservation action. We found evidence of declining populations for pinyon‐juniper‐associated species, highlighting the importance of considering these species when implementing conservation actions, despite an overall trend of increasing pinyon‐juniper woodland cover (Falkowski et al., 2017; Reinhardt et al., 2020). The density‐habitat relationships we developed provide correlational evidence that climate (*n* = 5 of 11 species), anthropogenic development (*n* = 8 species), and the proportion of pinyon‐juniper cover (*n* = 8 species) are associated with songbird densities throughout our study region (Figure 28, Table 6), and changes in environmental conditions may drive changes in community composition and species populations. Our maps of predicted bird density for 11 species provide novel resources to help inform conservation planning (Figures 30, 31, 32, 33, 34, 35, 36, 37, 38, 39, 40; Van Lanen et al., 2023b). We expect the mechanical removal of pinyon‐juniper to result in trade‐offs across the suite of species we evaluated within the sagebrush and pinyon‐juniper ecotones, with some species benefitting and others responding negatively.

### 4.1 Population trends

We found changes in regional populations among our study species occurred most frequently within the Northern Rockies (BCR10; *n* = 6), Southern Rockies/Colorado Plateau (BCR16; *n* = 4), and Shortgrass Prairie (BCR18; *n* = 6) BCRs. Interestingly, regional population trends were all increasing (*n* = 6) within the highest elevation BCR (BCR10) included in our study. Although we did not specifically evaluate the role of climate in these regional trends, there is growing evidence indicating a changing climate is affecting bird populations within the western United States (Betts et al., 2019; Illan et al., 2014). In response to a warming and drying climate, species may move upslope to match changing vegetation (Lenoir et al., 2008) or climatic conditions (Illan et al., 2014). Our findings support the possibility that immigration to the Northern Rockies may be occurring, as species seek refugia from a warming climate.

Despite widespread loss, fragmentation, and degradation of sagebrush habitat throughout the western United States (Knick et al., 2003; Remington et al., 2021; Schroeder et al., 2004), including degradation as a result of pinyon‐juniper woodland expansion (Falkowski et al., 2017), we found little evidence of population declines from 2008 to 2020 among the four sagebrush‐associated songbird species we investigated. We found evidence of three increasing populations within BCRs (Brewer's Sparrow and Sage Thrasher in BCR10 and Sage Thrasher in BCR17) and two decreasing populations (Brewer's Sparrow in BCR11 and Sage Thrasher in BCR18). Additionally, considerable evidence indicates the distribution of pinyon‐juniper woodlands is expanding and overall pinyon‐juniper cover may be increasing (i.e., infilling) (Miller et al., 2017, 2019; Reinhardt et al., 2020). We expected the expansion of pinyon‐juniper woodlands would result in increasing populations of pinyon‐juniper associated species as more habitat for these species becomes available. However, our trend estimates for these species revealed mixed results. We found evidence of both Gray Flycatcher and Gray Vireo populations increasing in at least one BCR while decreasing in one or more BCRs within our study area. Thus, expanding pinyon‐juniper habitat has not resulted in ubiquitous increases in pinyon‐juniper‐associated species. A closer investigation into potential mechanisms driving negative population trends may assist land managers in stabilizing declining populations. Additionally, although pinyon‐juniper woodlands are expanding at large spatial extents, there are regions experiencing woodland reductions due to drought, tree harvest, and management (Amme et al., 2020; Mueller et al., 2005; Reinhardt et al., 2020). Explicit mapping of pinyon‐juniper infilling, expansion, and retraction may help reveal links between changes in tree cover and regional population trends of pinyon‐juniper‐associated species.

The trends we developed in this study differed from BBS population trends for several species (Sauer et al., 2020). The disparate temporal extents examined (2008–2020 in our study; 1966–2019 for BBS trends) almost certainly contributed to these differences. Specifically, species exhibiting negative BBS trends but stable trends in our study may be stabilizing (e.g., Brewer's Sparrow and Sagebrush Sparrow) or even increasing (Green‐tailed Towhee) in the western United States over the last decade or more. In this case, the negative BBS trends may be driven largely by declines occurring between 1966 and 2008.

We also note differences in response variables (density versus abundance indices), survey methodology, sampling design, and analytical approaches between the BBS and IMBCR programs may contribute to a lack of congruence among trend estimates. For instance, surveys for the BBS program are conducted along roadsides, while IMBCR sample locations are selected irrespective of roadways (Pavlacky Jr. et al., 2017). This difference in sampling may result in biased population and trend estimates for species which preferentially select or avoid roadways (Fahrig & Rytwinski, 2009). The IMBCR program also employs field methods which allowed us to directly account for incomplete availability and detectability of individuals, whereas BBS trends are derived from indices of abundance with detection offsets (Sauer et al., 2020). In our analyses, we noted observer experience (*n* = 9 species) and minutes since sunrise (*n* = 11 species) influenced the detectability of species in our study. The BBS analyses do not model differences in these variables, however do model observer as a random variable (Sauer et al., 2020) and attempt to control for differences in daily detection rates and seasonal availability via sampling procedures. Specifically, BBS protocol targets daily and seasonal survey windows (often the month of June; Robbins et al., 1986, Sauer et al., 2017). However, our results suggest availability varied considerably throughout June (corresponding to ordinal days = 153–182) for Gray Flycatcher (Figure 5), Green‐tailed Towhee (Figure 7), Sagebrush Sparrow (Figure 10), and Sage Thrasher (Figure 11). We believe our results indicate the utility of accounting for seasonal and temporal variation in analyses and/or surveying over a short seasonal window during the summer sampling season.

### 4.2 Density‐habitat relationships

Our density‐habitat relationships for pinyon‐juniper and sagebrush‐associated species largely adhered to prior habitat descriptions (Partners in Flight, 2021), with the exception that Bewick's Wren (considered a generalist; Partners in Flight, 2021) was positively associated with the proportion of pinyon‐juniper and Sagebrush Sparrow (considered a sagebrush obligate; Partners in Flight, 2021) was negatively associated with sagebrush cover in our study. Classifying Bewick's Wren as a generalist throughout its entire range may be appropriate, yet they appear more strongly associated with pinyon‐juniper habitats within the eastern portion of their range. Indeed, an investigation into nest site selection in Oklahoma noted Bewick's Wren use of juniper habitat (Pogue & Schnell, 1994) as did a study on Bewick's Wren habitat use in southwestern Wyoming (Pavlacky & Anderson, 2001), which both agree with our findings. The negative association between Sagebrush Sparrow density and sagebrush cover we found may be an artifact of the zero‐inflation component included in our model. Our predicted Sagebrush Sparrow density map indicates this species is locally abundant but restricted to relatively confined areas. Due to the zero‐inflation component of our model, relationships between covariate values and species density were developed within suitable geographic areas for the species. Thus, Sagebrush Sparrow may be restricted to regions supporting sagebrush but within these regions are associated with lower sagebrush cover. Sagebrush Sparrow densities within sagebrush ecosystems in northwest Colorado and Wyoming were similarly associated with reduced sagebrush cover in prior work (Aldridge et al., 2011; Timmer et al., 2019; Williams et al., 2011). One recent study demonstrated Sagebrush Sparrow prefer ground foraging among canopy gaps, and walking to and from active nests (Martin & Carlson, 2020), which may be facilitated by lower sagebrush canopy cover within a matrix of sagebrush habitats.

Pinyon‐juniper removal to enhance Greater Sage‐grouse habitat often targets sites where pinyon‐juniper is expanding into existing sagebrush ecosystems (Natural Resource Conservation Service, 2015, Severson et al., 2017). These sites are often characterized as Phase I or Phase II pinyon‐juniper habitat (Miller et al., 2019) and possess both existing sagebrush understory and pinyon‐juniper vegetative cover (Miller et al., 2019; Roundy et al., 2014). Given removal of pinyon‐juniper from early successional pinyon‐juniper woodlands (Reinhardt et al., 2020), the species we predict to occur at highest densities within Phase I and Phase II pinyon‐juniper woodlands (Figure 29) will likely experience the largest negative effects from these treatments. Our model results and those associated with our companion study on Pinyon Jay density‐habitat relationships (Van Lanen et al., 2023a) indicate pinyon‐juniper removal may result in the largest reduction of Bewick's Wren, Black‐throated Gray Warbler, Juniper Titmouse, Gray Flycatcher, and Pinyon Jay densities, in regions where they occur. Conversely, we show that species such as Brewer's Sparrow, Sage Thrasher, and Green‐tailed Towhee all occur at highest densities within sagebrush ecosystems lacking pinyon‐juniper and will likely benefit from pinyon‐juniper removal treatments. Our inference regarding expected species' responses to pinyon‐juniper removal largely agree with prior experimental studies and modeling efforts (Holmes et al., 2017; Magee et al., 2019; Zeller et al., 2021). These previous studies and our findings highlight the utility in considering effects to non‐target species when conducting large‐scale habitat manipulations, particularly pinyon‐juniper removal, which we recognize is likely necessary to maintain adequate extents of sagebrush ecosystems in support of associated wildlife species. Targeting pinyon‐juniper removal within sites where we predict low densities of Bewick's Wren, Black‐throated Gray Warbler, Juniper Titmouse, Gray Flycatcher, and Pinyon Jay may therefore minimize unwanted effects of pinyon‐juniper removal to non‐target species. Future work evaluating where pinyon‐juniper and sagebrush systems may be compatible with future climates could facilitate proactive vegetation management which aligns with future abiotic conditions, thereby increasing the longevity of mechanical removal treatments and ensuring there is adequate early successional pinyon‐juniper woodlands for species occurring along the sagebrush and pinyon‐juniper ecotone.

We found point and/or linear anthropogenic disturbances likely contributed to lower population densities in the majority (*n* = 6 of 11) of our study species (Figures 14, 15, 16, Table 6), which agrees with prior studies demonstrating reduced density and occupancy rates among songbirds in regions of high anthropogenic development (Gilbert & Chalfoun, 2011; Mutter et al., 2015). Brewer's Sparrow demonstrated a particularly strong negative relationship with point and linear disturbance. Prior findings indicate Brewer's Sparrow habitat suitability is positively influenced by increasing patch size (Knick & Rotenberry, 1995), which linear disturbance reduces via fragmentation. Only two species were positively associated with linear disturbance (Juniper Titmouse and Sagebrush Sparrow) and no species were positively associated with point disturbance in this study. As indicated previously, Sagebrush Sparrow prefer patches with a more open understory (Williams et al., 2011) and less sagebrush cover (Aldridge et al., 2011; Timmer et al., 2019), both of which can be increased with increased density of unpaved roads. Juniper Titmouse may respond positively to high transmission line densities (Zeller et al., 2021), which agrees with our findings, since transmission lines were included in our linear disturbance feature layer. Additionally, the negative association between density and increasing linear and/or point disturbance we observed for many species indicates there could be less interspecific competition, and therefore competitive release, for Juniper Titmouse in roaded areas. We chose to include anthropogenic features in our analyses based upon prior findings that roads and well pads may influence songbird occupancy and abundance (Gilbert & Chalfoun, 2011; Johnson & Balda, 2020; Mutter et al., 2015); however, the large associated uncertainty of our estimates demonstrates the difficulty in such assessments. Since IMBCR data were not designed to specifically evaluate anthropogenic effects on wildlife, we had relatively few samples within heavily developed areas. Robust model‐based inference requires samples to appropriately represent the population of inference (Williams et al., 2019), which would include a larger number of data points within regions of high anthropogenic disturbance. Thus, we conclude that studies seeking to evaluate wildlife response to anthropogenic disturbance will likely benefit from implementing stratified sampling across disturbance intensities, to ensure a more uniform distribution of covariate values among sampled units.

We found cooler and wetter weather (higher PDSI values) was associated with slight to moderate increases in density for five of our study species, while no species increased with warmer and drier conditions. Our findings are consistent with recent research which found drought‐induced reductions in reproduction, survival, and abundance among songbirds (Albright et al., 2010; Martin & Mouton, 2020). The effect of climate on avian abundance we found indicates drought may alter the overall composition of avian communities in the western United States. Given these relationships, we expect less representation of species negatively affected by drought in the overall songbird community as the western United States continues to warm and dry (Kharin et al., 2007). We note similar patterns have been found elsewhere (Lindström et al., 2013). Interestingly, all five species positively associated with cooler and wetter weather in our study also demonstrated increasing population trends within the Northern Rockies (BCR10), the highest elevation region included in our study. Thus, high‐elevation sites may serve as refugia for species negatively affected by increased drying and/or warming. Additional vegetation modeling under climate change scenarios may guide forward‐thinking conservation planning and land management efforts.

### 4.3 Study limitations

Our inference regarding species' density‐habitat relationships and associated implications for management was limited in several ways. First, our predictions regarding use of various pinyon‐juniper woodland phases would be strengthened by modeling density as a function of pinyon‐juniper woodland phase explicitly. Unfortunately, to our knowledge, time‐stamped layers of pinyon‐juniper woodland phases across the western United States do not currently exist. Development of such products could improve our collective understanding of wildlife use along sagebrush and pinyon‐juniper ecotones (see Figure 29) and wildlife response to pinyon‐juniper treatments. Additionally, we used spatial layers in our modeling approach to predict densities of songbirds across our study area, including at unsampled locations. The layers we used unfortunately lacked information on vegetation height, stand age, mast production, and species composition; all variables which undoubtedly influence avian densities. Fine‐scale studies regarding the influence of these resource conditions could help advance our collective knowledge regarding the importance of pinyon‐juniper successional stages for wildlife and may prove useful in evaluating transferability of our modeled habitat relationships across broad spatial extents.

Our density‐habitat relationships were limited by a relative paucity of IMBCR data within Nevada, Arizona, and New Mexico. These regions, as well as regions of Oregon, likely represent important habitat for many of the pinyon‐juniper associated species we evaluated. Therefore, additional investigations into density‐habitat relationships for these species, informed by data collected in the southwestern United States, may prove important in evaluating the utility and transferability of our modeled relationships within these regions.

We recognize a changing climate and anthropogenic development are rapidly altering resource conditions in our study area. Climate change and development could result in new combinations of environmental variables compared to those which occurred historically (i.e., “novel ecosystems”; Hobbs et al., 2006). Novel ecosystems may lead individuals to select breeding sites using cues which today poorly relate to habitat quality (Battin, 2004). Although there has long been concern over the ability of density metrics to infer habitat quality (Van Horne, 1983), we suspect such concerns may be increasingly warranted given the rise of these novel ecosystems. Additional efforts evaluating demographic rates for wildlife species within sagebrush and pinyon‐juniper ecotones, including experimental settings with vegetation treatments, may provide important information regarding the role of pinyon‐juniper successional stages in maintaining viable wildlife populations and wildlife response to pinyon‐juniper removal.

Finally, our study did not assess the influence of cascading effects a changing climate and the spread of invasive annual grasses may have on wildfire risk. As the western United States is expected to warm and dry under a changing climate (Kharin et al., 2007), wildfires are expected to occur more frequently and burn larger geographic extents (Liu & Wimberly, 2016). Concurrently, invasive annual grasses, including cheatgrass, serve as both an ignition source (Bradley et al., 2017) and increasingly invade areas post fire (Peeler et al., 2018). Together, a changing climate and spreading invasive annual grasses may therefore facilitate a positive feedback loop of increasing invasive grasses and wildfire frequency and extent. Wildfires, in turn, are liable to reduce woody vegetation, including both the sagebrush percent cover and the proportion of pinyon‐juniper variables we assessed. Unfortunately, we were not able to address these cascading indirect effects of a changing climate in our study, despite strong evidence the interplay among climate change, cheatgrass, and wildfire poses a severe risk to both sagebrush and pinyon‐juniper ecosystems (Pilliod et al., 2021; Redmond et al., 2023).

### 4.4 Predictive maps

Our 11 predicted density maps could be used to identify important regions for each species, help managers identify regions where environmental perturbations may most affect species of conservation concern, and inform conservation planning (Van Lanen et al., 2023b). We note relatively high levels of uncertainty associated with our predicted densities, particularly within localized regions characterized by high point and/or linear disturbance. We initially sought to mask out predictions to all areas where spatial layer values exceeded the 95% quantile of covariate inputs used to fit each model; however, this resulted in the masking of a large proportion of the landscape due to the substantial number of covariates included in the models and significant covariate variation across the landscape. To address this, we restricted the masking of regions to those with extremely high point disturbance, as point disturbance features on the landscape exceeded 130 standard deviations above the mean values used to train the model, but also present a masking layer indicating all pixels where one or more covariates exceed the 95% quantile of data used to fit the model (Van Lanen et al., 2023b). Doing so enables potential users to decide which mapped predictions are appropriate for use in decision making.

## 5 CONCLUSIONS

Our investigations reveal expansion of pinyon‐juniper cover into sagebrush systems is likely to influence populations of sagebrush and pinyon‐juniper associated species, as are ongoing pinyon‐juniper removal projects. The numerous negative density relationships with drought severity and anthropogenic development (both point and linear) we found indicate negative effects of these changes may be widespread and additional habitat for many species may become degraded in the future. These environmental perturbations may result in the co‐occurrence of declining species, associated with pinyon‐juniper or sagebrush ecosystems, more frequently. Making place‐based decisions regarding what ecosystem and species to manage for within sagebrush and pinyon‐juniper ecotones is likely to become increasingly difficult. Our efforts here provide a foundation for identifying non‐target species which may be detrimentally affected by pinyon‐juniper removal activities and quantifying potential benefits for target species. We suggest our framework for trend estimation, density‐habitat relationship modeling, and predictive density mapping can help prioritize species and regions for conservation action, evaluate how changing environmental conditions may affect wildlife, infer effects of habitat restoration projects on both target and non‐target species, and balance species' habitat requirements across ecosystems. We believe information gleaned from this approach will be critical for balancing needs of declining species with disparate habitat requirements so biodiversity may be conserved for future generations.

## AUTHOR CONTRIBUTIONS

**Nicholas J. Van Lanen:** Conceptualization (lead); data curation (lead); formal analysis (lead); funding acquisition (supporting); investigation (lead); methodology (equal); project administration (supporting); resources (supporting); software (lead); validation (lead); visualization (lead); writing – original draft (lead); writing – review and editing (lead). **Adrian P. Monroe:** Formal analysis (supporting); investigation (supporting); methodology (supporting); software (supporting); validation (supporting); visualization (equal); writing – review and editing (supporting). **Cameron L. Aldridge:** Conceptualization (supporting); formal analysis (supporting); funding acquisition (lead); investigation (supporting); methodology (supporting); project administration (lead); resources (lead); supervision (lead); validation (supporting); visualization (supporting); writing – original draft (supporting); writing – review and editing (supporting).

## CONFLICT OF INTEREST STATEMENT

The authors certify they have no conflict of interest in the subject matter or materials discussed in this manuscript.

## Data Availability

Summarized data and model code to replicate analyses are available as a U.S. Geological Survey data release at https://doi.org/10.5066/P9KFCBLH (Van Lanen et al., 2023c). Raw bird data may be available from Bird Conservancy of the Rockies by completing the form located at ADC Query (rmbo.org) or by emailing the point of contact listed on the Integrated Monitoring in Bird Conservation Regions webpage (IMBCR Program – Connecting People, Birds and Land for a Healthy World (birdconservancy.org)). Restrictions apply to the availability of these data, which we acquired under a data sharing agreement for this study. At the time of publication, raw disturbance data used in this study are sensitive and cannot be provided publicly. These data may be made available to qualified researchers from the Bureau of Land Management by contacting the Wildlife Habitat Spatial Analysis Lab at the National Operations Center (National Operations Center | Bureau of Land Management (blm.gov)). LANDFIRE existing vegetation type data are published and publicly available for download at LANDFIRE Program: Data Product Mosaic Downloads. The RCMAP vegetation layers are published and publicly available for download at Rangeland Condition Monitoring Assessment and Projection (RCMAP) Fractional Component Time‐Series Across the Western U.S. 1985–2020 – ScienceBase‐Catalog. Specifically, we used the “Rangeland Condition Monitoring Assessment and Projection (RCMAP) Fractional Component Time‐Series Across the Western U.S. 1985–2020” layers associated with annual herbaceous, bare ground, herbaceous, litter, and sagebrush. The Palmer Drought Severity Index (PDSI) data, and accompanying climate division shapefiles, we used in analyses can be downloaded by visiting Index of/pub/data/cirs/climdiv (noaa.gov) and selecting “CONUS_CLIMATE_DIVISIONS.shp.zip” (climate division polygons) and the most recent PDSI link (e.g., “climdiv‐pdsidv‐v1.0.0–20,230,206” at the time of submission).
